# Characterization and functional analyses of wheat *TaPR1* genes in response to stripe rust fungal infection

**DOI:** 10.1038/s41598-023-30456-8

**Published:** 2023-02-27

**Authors:** Rong Liu, Jing Lu, Jiayi Xing, Lv Xue, Yu Wu, Lei Zhang

**Affiliations:** 1 Faculty of Agriculture, Forestry and Food Engineering of Yibin University, Yibin, 644000 China; 2 Chengdu Institute of Biology, Chinese Academy of Sciences, Chengdu, 610041 China; 3 Innovation Academy for Seed Design, Chinese Academy of Sciences, Beijing, 100101 China

**Keywords:** Plant breeding, Plant genetics

## Abstract

The pathogenesis-related protein-1 (PR1) gene is important for plants to respond to various biotic and abiotic stresses. Unlike those in model plants, PR1 genes in wheat have not been systematically studied. Herein, we identified 86 potential *TaPR1* wheat genes using bioinformatics tools and RNA sequencing. Kyoto Encyclopedia of Genes and Genomes analysis indicated that the *TaPR1* genes were involved in the salicylic acid signalling pathway, MAPK signalling pathway, and phenylalanine metabolism in response to Pst-CYR34 infection. Ten of the *TaPR1* genes were structurally characterized and validated by RT‒PCR. One particular gene, *TaPR1-7,* was found to be associated with resistance to *Puccinia striiformis* f. sp. *tritici* (Pst) in a biparental wheat population. Virus-induced gene silencing showed that *TaPR1-7* is important for Pst resistance in wheat. This study provides the first comprehensive study on wheat PR1 genes, improving our overall understanding of these genes in plant defenses, particularly against stripe rust.

## Introduction

Wheat (*Triticum aestivum* L., 2n = 6X = 42) is an important staple food for mankind that has a complicated origin and is widely planted throughout the world. The global annual output of wheat exceeded 700 million tons (https://crops.extension.iastate.edu/faostat) in 2018. However, the growth and yield of wheat is severely affected by various biotic and abiotic stresses, such as fungi, aphids, mosaic viruses, bacteria, mycosis, drought, high temperature, waterlogging, and saline-alkali stress. Stripe rust, stem rust, leaf rust, powdery mildew, and head blight are the three most common fungal diseases in wheat. To protect themselves against attacks from these pathogen, wheat has developed a series of complex response mechanisms.

Stripe rust, caused by *Puccinia striiformis* f. sp. *tritici* (Pst), is a parasitic fungal disease that occurs in wheat, barley, rye, and certain graminaceous hosts^1–3^. Wheat stripe rust fungus has become the largest biological limitation of global wheat production^4^ and can reduce wheat yield by 10%-70% and even decrease yield by 100% during a Pst outbreak^5^. It has become increasingly important to identify new resources for and mechanisms of resistance and new transgenic strategies to modify wheat varieties to limit damage caused by stripe rust. A further in-depth understanding of the molecular basis for controlling stripe rust infection in wheat and other gramineous plants will help implement these strategies.

Pathogenesis-related (PR) proteins, which include proteins such as glucanases, chitinases, peroxidases, thaumatin-like proteins, and proteases, are found throughout the wheat plant and include 17 protein families that perform a wide range of activities^6^. PR proteins have been reported to play an important role in plant defense against various biotic and abiotic stresses^7^. They can be activated in plants to respond to pathogen attacks. The expression level of PR gene transcripts is one of the best-characterized indicators for plant defense responses in many plant pathogen systems. The PR1 gene is the main member of the PR gene family and has been used as a marker for enhanced defense conferred by pathogen-induced systemic acquired resistance (SAR) in various plants^8^. Since the first PR1 was identified in tobacco^9^, the respective PR1 proteins or other PR gene family members have also been successively revealed in other plant species, such as *Arabidopsis thaliana*^10^, tomato^11,12^, rice^13^, and mulberry^14^. Studies have shown that PR1 is a salicylic acid (SA)-responsive gene that is a major player in the SA pathway^15^, and it can be activated by the application of SA in wheat^16^. PR1 was linked to both race-specific all-stage resistance and nonrace-specific high-temperature adult-plant (HTAP) resistance mediated by different Yr genes in wheat^17,18^. In addition, PR1 was reported to inhibit the process of programmed cell death and suppress bacterial pathogens in plant tissues^19^. However, only a few studies investigating the role of most PR1 genes in the wheat-Pst interaction have been performed. Previous studies have found that the expression of the PR1 gene, which is possibly modulated by WRKY6, ultimately leads to cell death to benefit the infection and growth of necrotrophic fungi^20,21^. The molecular mechanisms of the PR1 gene that underlie disease resistance need to be further investigated.

In recent decades, high-quality reference genome sequences and annotations for many plant species, such as wheat, have become available, which provides a resource for the systematic investigation of PR gene families. In this study, we took advantage of the common wheat reference genome sequence to mine all PR1 genes followed by genetic and functional analyses of some PR1 genes in the reaction to Pst. The results of this research will enrich the R gene database, provide a theoretical basis for further research on *TaPR* genes, and accelerate future work on disease resistance breeding in wheat.

## Results

### Identification of *TaPR1* genes in wheat

A total of 86 *TaPR1* gene members in the wheat genome (IWGSC RefSeq V1.1) were identified through local Basic Local Alignment Search Tool Protein (BLASTP) identification and subsequent conserved domain database (CDD) and Pfam domain searching processes. Then, we renamed these *TaPR1* members (*TaPR1-1* to *TaPR1-86*) following the general nomenclature rule for wheat genes. The details of the *TaPR1* genes/proteins are listed in Table S1. *TaPR1* members have 1–8 coding exons (74 *TaPR1* genes contain only 1 exon, 9 *TaPR1* members contain 2 exons, 1 *TaPR1* member contains 3 exons, and 2 members contain 8 exons). The lengths of the transcripts ranged from 474 to 2603 nucleotides; the isoelectric point (PI) values ranged from 4.26 to 11.62; the molecular weights (kDa) ranged from 16.88 to 80.84; and the amino acid lengths ranged from 157 to 747 amino acids. The subcellular localization of 86 TaPR1 proteins was submitted to the online website for prediction. The expression of all of the TaPR1 proteins was localized to the extracellular in wheat.

**Figure 1:**
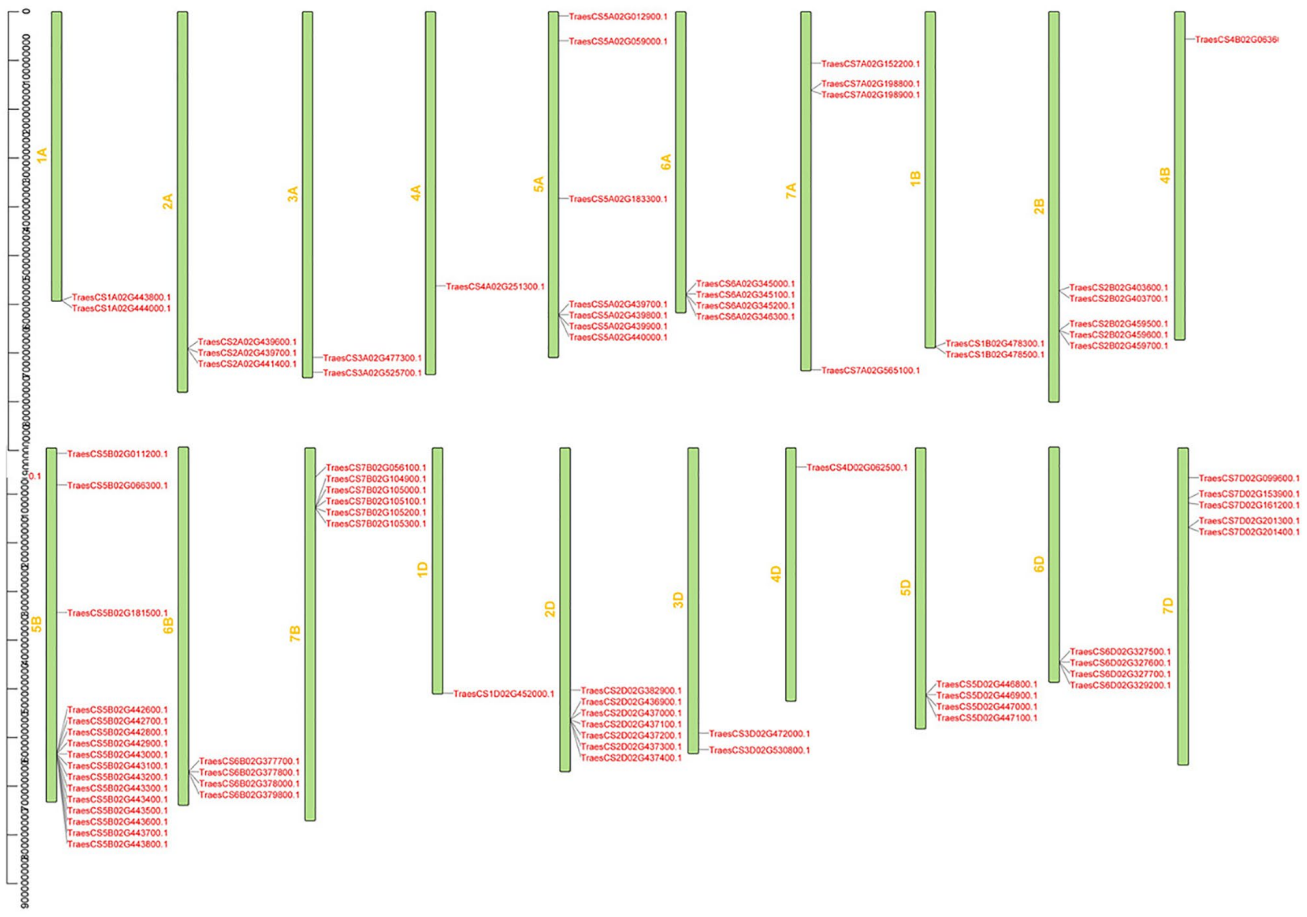
Chromosomal localization of the *TaPR1* genes in wheat. The light green column represents the chromosome. This figure was created by using TBtools.

The 86 *TaPR1* genes were located on chromosomes according to their positions, which were determined using the wheat genome database (IWGSC v1.1); the results are shown in Fig. 1. The predicted *TaPR1* genes were mapped to the A, B, and D genomes of wheat (except for chromosome 3B). Among them, 34 *Ta*PR1 genes were located in the B genome, 23 in the A genome, 24 in the D genome, and the remaining genes were located on unanchored scaffolds. Chromosome 5B contained the largest number of *TaPR1* genes (16), followed by 2A and 5A, which each had 7 *TaPR1* members. A chromosome region containing two or more PR1 genes within 200 kb is defined as a tandem repeat event. As shown in Fig. 1, most members of *TaPR1* are present as tandem repeats.

### Gene structure and conserved sequence analysis of the wheat PR1 gene

**Figure 2:**
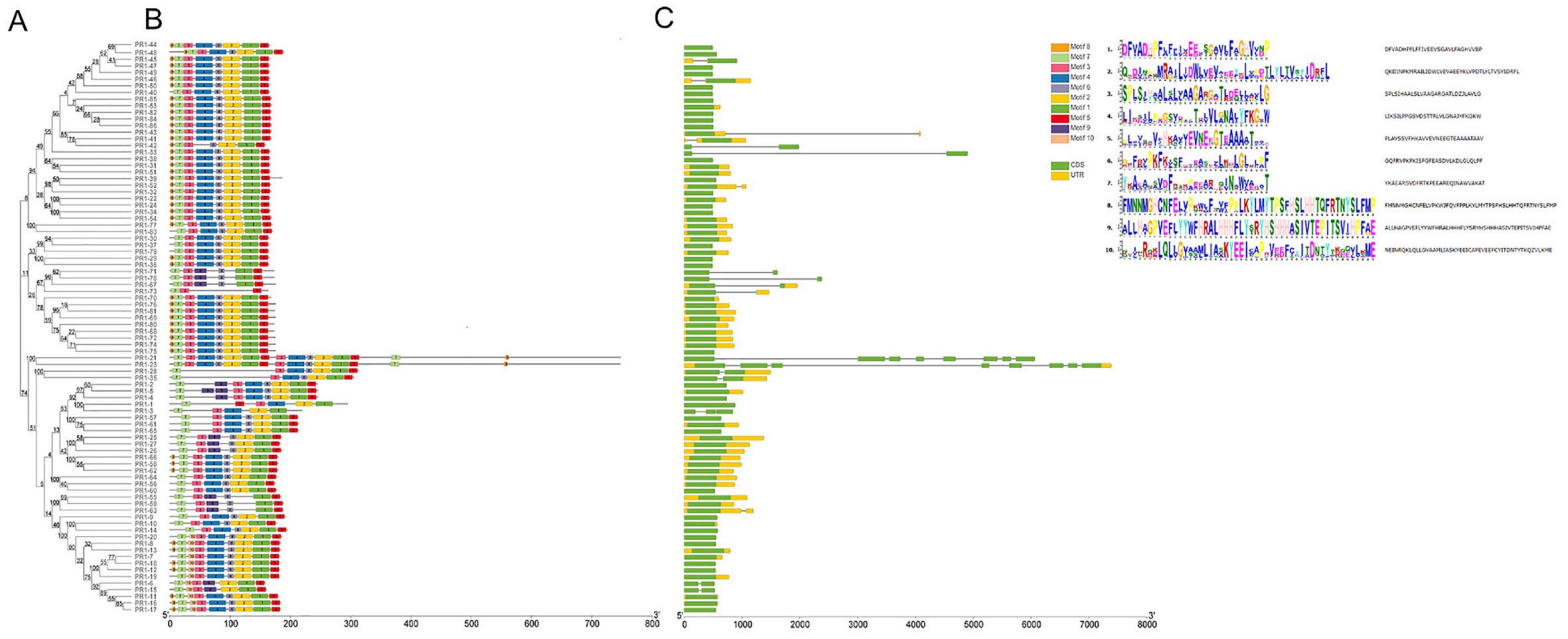
Conserved motif (**B**) and gene structure (**C**) analysis of *TaPR1* genes based on phylogenetic relationships (**A**). Each motif is represented by a coloured box with different numbers. The length of the box corresponds to the motif length. Green boxes represent exons, and yellow boxes represent upstream/downstream sequences (UTRs). The gene and protein lengths are indicated by the scale at the bottom. This figure was created by using TBtools.

Sixty-three (73.26%) *TaPR1* genes did not contain intron structures, 16 (6.98%) *TaPR1* genes contained 1 intron, 1 *TaPR1* gene contained 2 introns, and 2 *TaPR1* genes contained 7 introns. The domain analysis of wheat PR1 members showed that all 86 *TaPR1* genes contained a complete CAP-PR1 domain (Fig. S1, Additional file 3), certain PR1 members as well as containing a PRK14950 PRK10263 domain, and two STKt_IRAK domains, which might mediate the targeting of substrate proteins for proteasome degradation. This result illustrates that the *TaPR1* family has a highly conserved domain. We used the multi EM for motif elicitation (MEME) motif search tool to further prediction and verified the motif domains of the members of the *TaPR1* family. The conserved motif compositions of all TaPR1 proteins were similar (all contained 1, 3, 5, and 7 motifs) (Fig. 2A,B), indicating that these four motifs were important components of PR1 protein sequences. Most of the *TaPR1* genes contain 2, 4, 6, and 8 motifs, which might also have important effects on protein function. According to the results of the motif classification analysis of the *TaPR1* members, the 86 *TaPR1* genes can be divided into three categories according to Fig. 2A,B. Among the members of the *TaPR1* gene family, those in the same subfamily shared similar genetic structures and closer phylogenetic tree clusters (Fig. 2A,B,C). In our results, most *TaPR1* gene families had the same gene structure and conserved domains, which indicates that their functions and conservation are highly similar and their sequences are highly conserved.

### Expression profiles of *TaPR1* genes during stripe rust infection in wheat

**Figure 3:**
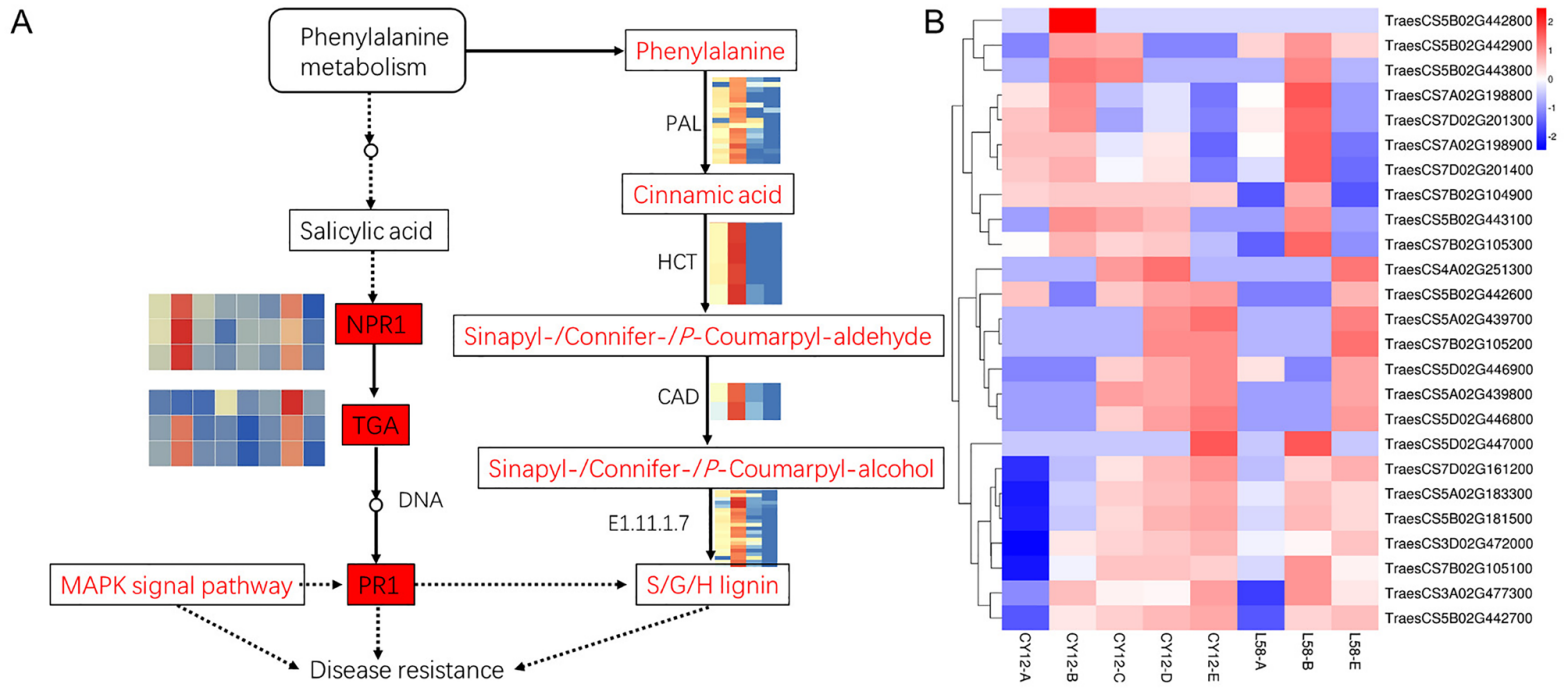
The metabolic pathway (**A**) and heatmap (**B**) of TaPR1 genes using their expression patterns in wheat. Log2 transformed FPKM values were used to create the heatmap. The small heatmap shown in the metabolic pathway represents the expression level of enriched genes related to this pathway. The red or blue colours represent the higher or lower relative abundance of each transcript in each sample. Results with P-values <  < 0.05 were regarded as statistically significant. This figure was created by using TBtools.

To explore the roles of the *TaPR1* genes in the response to stripe rust infection, we investigated their expression profiles in wheat by transcriptome sequencing (RNA-Seq) at 0 h (A), 24 h (B), and 7 days (C) after inoculation with Pst-CYR34. The results showed that phenylalanine metabolism, the SA pathway, and the MAPK signalling pathway were involved in the wheat response to stripe rust (Fig. 3A). In the present study, 24 h postinoculation (24 hpi) was found to be the key period for disease resistance in wheat. NPR1, TAG, and PR1 were all significantly up-regulated at 24 hpi (Fig. 3A, Additional file 2). Twenty-five *TaPR1* genes were identified in the RNA-Seq profile, most of which were up-regulated, including 17 genes at 24 hpi and 15 genes at 7 d postinoculation (dpi) in CY12 and L58 (Fig. 3B, Additional file 2).

The lignin synthesis branch of phenylalanine metabolism was involved in the resistance of wheat to Pst, which was studied by transcriptome analysis (Fig. 3A). The expression of genes encoding key enzymes involved in the lignin synthesis pathway were significantly up-regulated after Pst-CYR34 infection. In preliminary study results, we found that the accumulation of metabolites in lignin biosynthetic pathway as well as up-regulated by Pst infection in wheat seedlings. In addition, *TaPR1* is regulated by the MAPK signalling pathway. During the early stage, we also conducted an in-depth analysis of the MAPK signalling pathway and its involvement in wheat resistance to stripe rust.

### Phylogenetic and promoter analysis of PR1 genes in wheat

**Figure 4:**
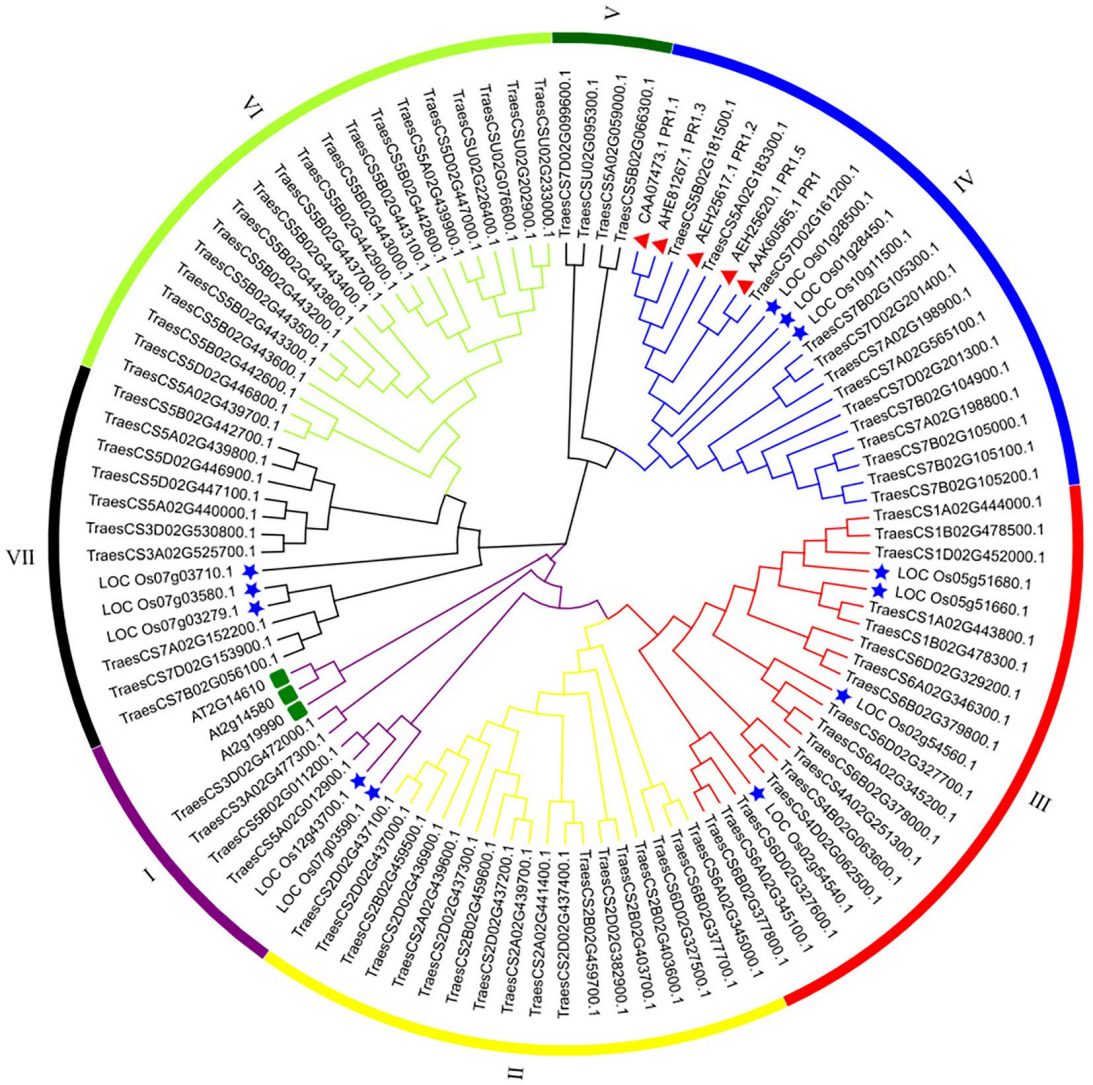
Phylogenetic tree of PR1 proteins in wheat, *Arabidopsis*, and rice. For PR1 proteins in wheat (prefixed by Traes-CS), *Arabidopsis* (prefixed by AT/At), and rice (prefixed by Os), both locus ID and subclass numbers are given. This figure was created by using TBtools.

All of the PR1 full-length amino acid sequences were used to construct a rootless phylogenetic tree to identify the evolutionary relationships of *TaPR1* genes in wheat, *Arabidopsis*, and rice. A total of 106 protein sequences from the above three species were included in the phylogenetic analyses, including 3 in *Arabidopsis*, 12 in rice, and 91 in wheat (86 *TaPR1* genes identified in this study, 5 *TaPR1* genes cloned in previous reports). Phylogenetic analysis showed that the predicted *TaPR1* regions in wheat can be divided into three major groups according to conserved motif domains and gene structure (Fig. 3A). The results in Fig. 4 show that PR1 proteins are monophyletic evolutionary branches, which can be further subdivided into 7 subcategories (I–VII). As shown in Fig. 4, TaPR1 has a corresponding sequence in each subcategory. Rice is mainly classified into four subcategories: I, III, IV, and VII, and *Arabidopsis* is classified into branch I. Among 86 TaPR1 proteins, 33 belong to the first group, 18 belong to the second group, and 35 belong to the third group. According to this analysis, the *TaPR1* genes in wheat were more closely related to those in rice than to those in *Arabidopsis*.

The potential cis-acting elements were predicted in the 2 kb sequence (noncoding region sequence) before the CDS (start codon) of the *PR1* gene family. A total of 11 main cis-acting elements were identified in the promoter regions of *PR1* family members, including abscisic acid-responsive, gibberellin-responsive, light-responsive, MeJA-responsive, MYBHv1 binding site, SA-responsive, AT-rich DNA binding protein (ATBP-1), auxin-responsive, defense and stress-responsive, zein metabolism regulation and auxin-responsive cis-acting elements (Fig. S2, Additional file 4). Among them, the greatest number of cis-acting elements were light-responsive, followed by those that were MeJA-responsive and gibberellin-responsive cis-acting elements, which were identified in TaPR1-10, 18, 19, 23, 25, 31, 33, 40, 45–50, 52–54, 61, 63, 68, and 72–75.

### Candidate *TaPR1* gene analysis by qRT‒PCR

**Figure 5:**
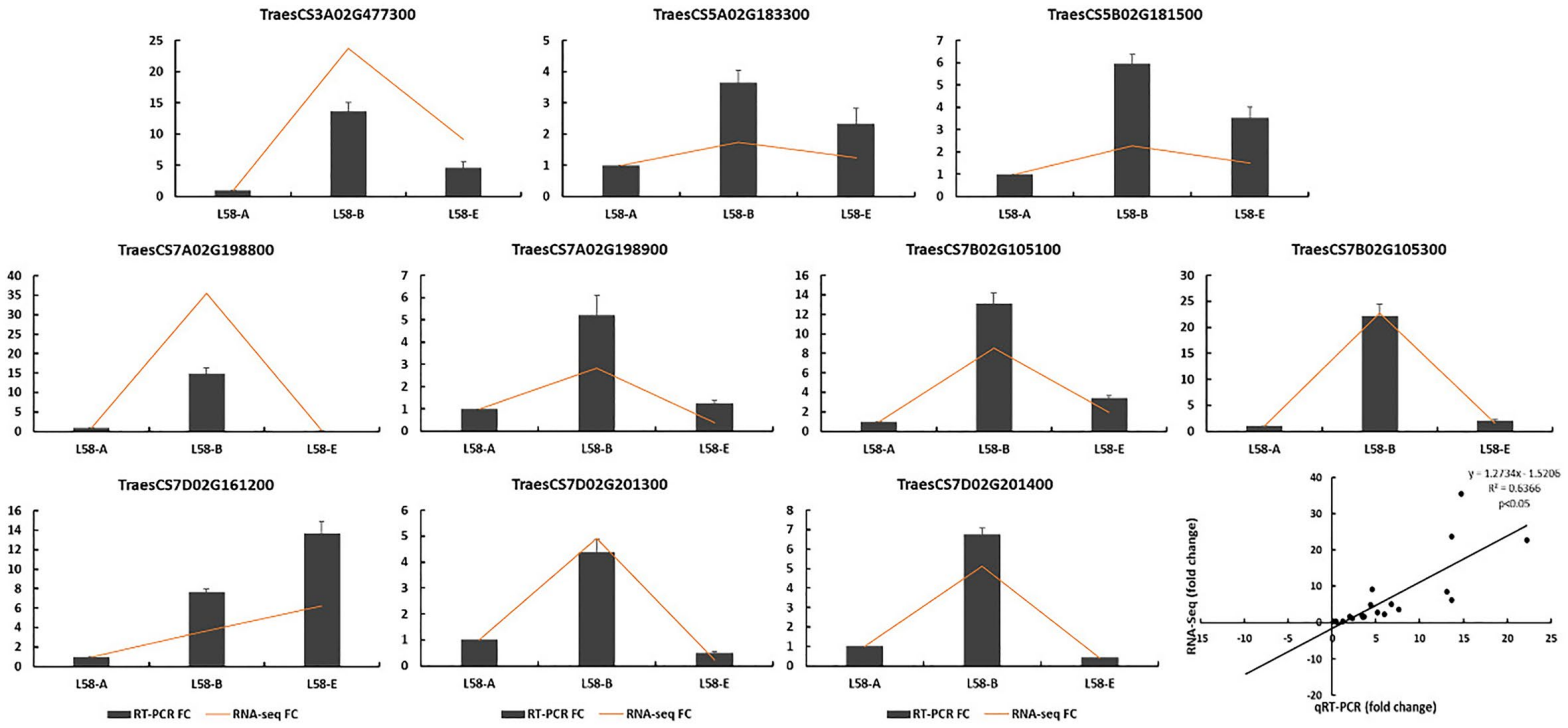
Verification of the expression level (fold change) of *TaPR1* by qRT‒PCR analysis. Relative expression levels of 10 *TaPR1* genes after pst-CYR34 infection. This figure was created by using TBtools.

To further verify these results, ten of the *TaPR1* genes were screened by qRT‒PCR to measure their expression levels in the leaves of wheat after Pst-CYR34 infection. All ten candidate *TaPR1* genes were up-regulated in L58 at 24 hpi (Fig. 5, Additional file 1). Six *TaPR1* genes, including *TraesCS3A02G477300*, *TraesCS5A02G183300*, *TraesCS5B02G181500*, *TraesCS7B02G105100*, *TraesCS7B02G105300* and *TraesCS7D02G161200*, were also significantly up-regulated at 7 days after Pst infection. The expression of the remaining four *TaPR1* genes was not significantly higher 7 days after Pst infection than in plants that were not inoculated. The expression of *TaPR1* genes measured by qRT‒PCR was highly consistent with the RNA-seq profile, which confirms that it is reasonable to use RNA-seq data to reliably evaluate the expression level of *TaPR1* genes in the wheat-Pst interaction.

### Cloning of candidate *TaPR1* genes and development of functional markers

Three homologous *TaPR1* genes in wheat, *TaPR1-7A*, *-7B*, and *-7D*, were selected and cloned according to their expression levels in the RNA-seq profile, and they were located on chromosomes 7A, 7B, and 7D, respectively. Phylogenetic analysis of the TaPR1-7A/7B/7D proteins from other plant species showed that the TaPR1-7A, TaPR1-7B, and TaPR1-7D proteins in wheat are closely related to PR1 proteins from *Arabidopsis*, *Zea mays,* and *Oryza sativa* (Fig. 4). The alignment similarity between the reference genome sequence and the sequences of *TaPR1-7A*, *TaPR1-7B*, and *TaPR1-7D* was more than 98% (Fig. S3). The sequences of the predicted TaPR1-7A/7B/7D proteins were determined to contain a conserved CAP domain, which could encode the PR1 protein in wheat.

**Figure 6:**
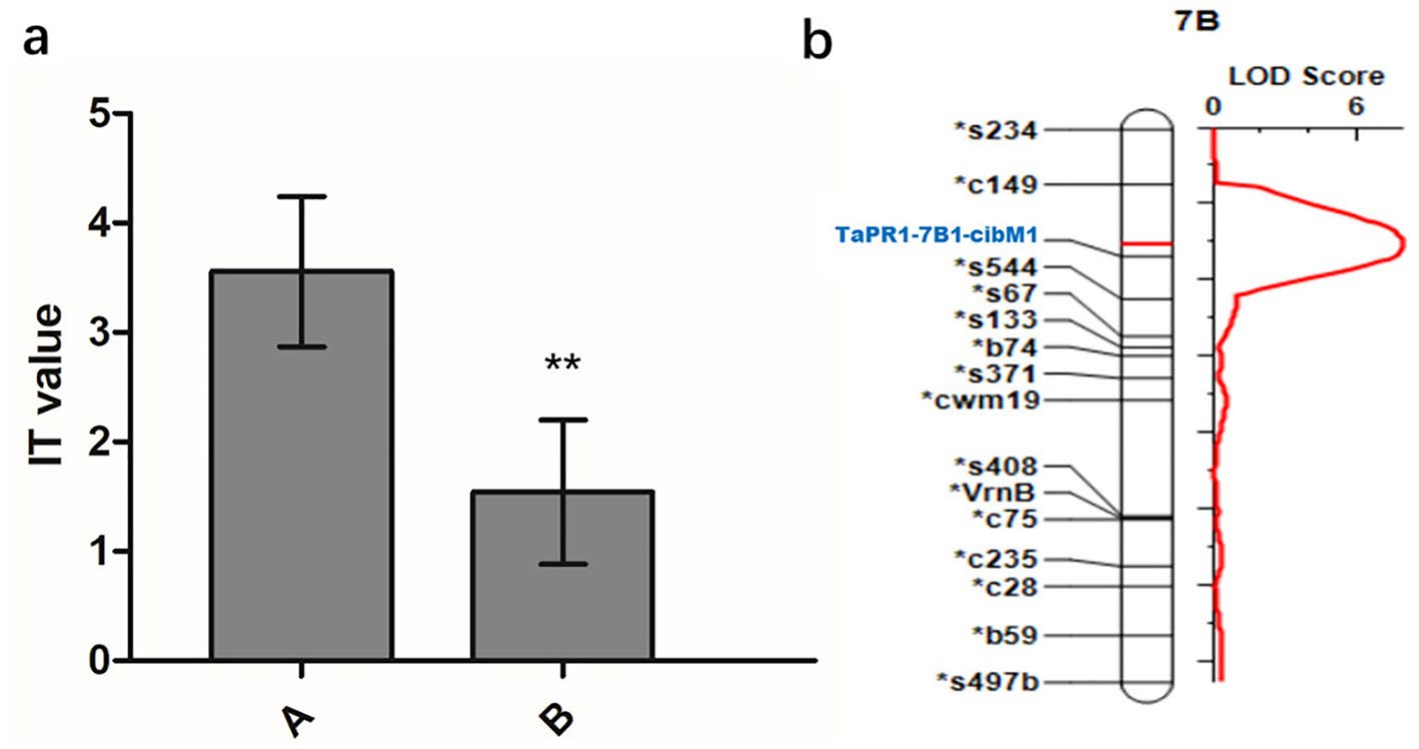
Analysis of the significance of *TAPR1-7B1-cibM1* in YZ1/NX188 RIL polymorphisms with the IT value of stripe rusts (t test, **P < 0.01) and the localized molecular linkage map on chromosome 7B in YZ1/NX188 RILs (b). This figure was created by using TBtools.

Based on the guidelines of the ‘Chinese Spring Reference Genome Sequence’, we designed primers (*TaPR1-7B1-cibM1*) with a 2 K region upstream of the coding sequence (CDS) of the *TaPR1-7B* gene to perform PCR amplification. The results showed that there was an insertion‒deletion of approximately 134 bp in susceptible wheat, unlike in resistant wheat (Fig. S4). Subsequently, we validated the RIL population constructed from resistant/susceptible wheat parents (YZ1/NX188 RILs). The results showed that approximately 89% of the phenotypes were consistent with this polymorphic marker (Fig. S4), indicating that this marker could be used as a breeding marker. Then, according to the polymorphism of molecular markers, the PCR product bands were divided into A and B bands (A represents susceptible wheat and B represents resistant wheat), and the gene was found to be located on chromosome 7BS by combining with the YZ1/NX188 (198 RILs) mapping population (Fig. 6). The results showed that molecular markers were significantly associated with stripe rust resistance. The LOD was 7.9267, with a PVE of 17.4573%. Therefore, these results indicated that *TaPR1-7B1-cibM1* was involved in the wheat stripe rust resistance response.

### Validation of candidate genes by VIGS

**Figure 7:**
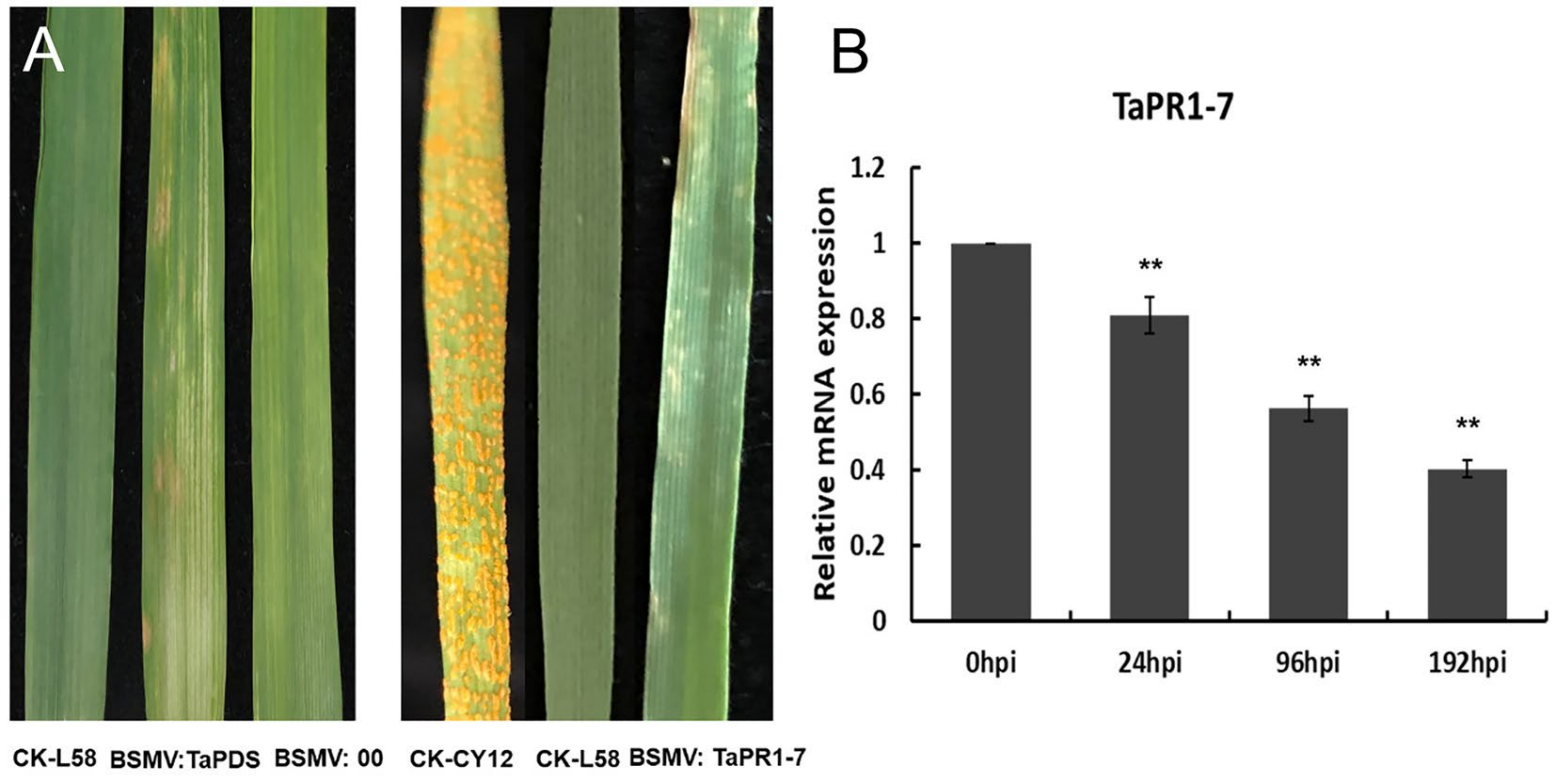
Phenotypes of the wheat leaves of BSMV-infected plants that were further inoculated with pst-CYR34 at 14 dpi and relative mRNA expression levels of *TaPR1-7* in *TaPR1-7*-silenced wheat plants inoculated with Pst-CYR34. The comparative threshold (2^−ΔΔCT^) approach was used to measure the relative mRNA expression of *TaPR1-7*. Data were normalized using the transcription level of *TaGAPDH* and visualized as the fold change compared to the control at 0 hpi. BSMV:00-infected wheat leaves were used as a negative control, and BSMV:TaPDS-infected wheat leaves were used as a positive control. Double asterisks (*P* < 0.01) denote significant differences.

To investigate whether the *TaPR1-7A/7B/7D* gene is involved in the regulation of wheat defense resistance against Pst, BSMV-VIGS was performed in this study. A specific gene fragment was designed to specifically silence all three copies of the endogenous *TaPR1-7* gene (*TaPR1-7A/7B/7D*) in wheat. Unlike in CK, wheat leaves inoculated with BSMV: 00 (negative control) and BSMV: TaPR1-7 displayed mild chlorotic mosaic symptoms at 14 dpi, while wheat leaves were bleached and faded after inoculation with BSMV: TaPDS (positive control) (Fig. 7). qRT‒PCR validated that the expression levels of *TaPR1-7* were significantly lower in the BSMV:TaPR1-7-silenced wheat lines than in the BSMV:00-treated line at 0, 24, 96, and 192 hpi (Fig. 7). The total transcription levels of three TaPR1 homologues in the leaf tissue of BSMV:TaPR1-7-inoculated wheat seedlings were significantly reduced by 59.8%, which suggests that *TaPR1-7A/7B/7D* was successfully silenced by BSMV-VIGS in wheat seedlings. Wheat leaves inoculated with CYR34 displayed necrosis symptoms in BSMV: TaPR1-7-silenced lines. However, the wheat leaves of the L58 (CK) line were immune to Pst-CYR34. These results indicated that *TaPR1-7A/7B/7D* was involved in the resistance of wheat to Pst-CYR34 and that it can positively regulate the resistance response in wheat.

## Discussion

### Characteristics of PR1 genes in wheat

PR1 is a gene family widely present in all plant species. The *PR1* gene is involved in an important function of plant stress resistance^22^ and plays critical roles in regulating plant responses to biotic and abiotic stresses^23,24^. *PR1* genes have been identified in many higher plants. To study the structure and function of wheat *PR1* genes, 86 *TaPR1* genes were identified in wheat. According to van Loon et al.^25^, PR proteins are generally low-molecular-weight (43 kDa), protease-resistant, thermostable, and soluble at low pH^3^. Only one or two exons were identified in wheat PR1 genes, and the protein lengths ranged from 157 to 747 amino acids, which is consistent with a previous study^12^. The findings are also consistent with other previous findings, namely, that PR1 proteins primarily accumulate and are secreted in the extracellular/apoplastic space, which is facilitated by their N-terminal secretion peptide^12^. Based on the comparison of the number of *TaPR1* genes and other monocot genome sequences, tandem and segment repetition events play an important role in the amplification of the *PR1* gene family. Genome-wide replication events are a common phenomenon in the evolution of angiosperms and usually lead to the expansion of gene families^26^. This finding may also explain why the number of *TaPR1* genes identified in wheat is greater than that in other monocots.

### Expression patterns of the PR1 genes in wheat in response to Pst

The transcription level of PR1 has been extensively used as a marker for disease resistance in plants^27^ without evidence of the presence of the protein or a direct effect on either the disease or pathogen dynamics^28^. Therefore, 86 *TaPR1* genes were identified to predict and verify their actual functions in this study. Approximately 2% of the total leaf proteins in pathogen-infected tobacco plants were PR1 proteins produced through the defense response^29^. *TaPR1* transcript accumulation was found to be high in wheat lines resistant to stripe rust, *Fusarium graminearum* Schw, *Mycosphaerella graminicola*, and powdery mildew^18,30–32^. The expression of the PR1 protein-like gene was up-regulated in both resistant and susceptible genotypes of tomato under *Alternaria solani* infection^33^. Similar to a previous report, approximately 25 *TaPR1* genes were up-regulated in both CY12 and L58 after Pst-CYR34 infection by RNA sequencing. Among them, *TraesCS5A02G183300*, *TraesCS5B02G181500*, *TraesCS7A02G198800*, *TraesCS7A02G198900*, *TraesCS7D02G201300* and *TraesCS7D02G201400* were highly expressed at 24 hpi in wheat and had different expression patterns during different stages of Pst infection. According to KEGG analysis, all *TaPR1* genes were involved in the SA signalling pathway (Fig. 2), which is known to induce wide-spectrum disease resistance in various plants and to regulate pathosystems^34^. Therefore, we inferred that the expression levels of certain *TaPR1* genes such as *TraesCS5A02G183300*, *TraesCS5B02G181500*, *TraesCS7A02G198800*, *TraesCS7A02G198900*, *TraesCS7D02G201300* and *TraesCS7D02G201400* are essential for the resistance of wheat to stripe rust.

In addition, the increase in the expression level of *TaPR1* genes is related to lignin accumulation during fungal infection (CYR34) in wheat, which is also involved in the transcriptional regulation of specific genes during pathogen invasion. Our results were consistent with those of a previous study. As lignin levels increase, the SA and JA pathways are activated to inhibit AUX/IAA, and plant resistance to pathogens is increased^35^. According to our study, *TaPR1* gene expression may activate plant lignin accumulation in response to CYR34 infection. The spatial expression pattern indicates that *TaPR1* regulates the accumulation of gene products that interact with regulatory genes that mediate cell wall modification in plants^18^.

### Conservation and evolution of the PR1 gene family in wheat

According to previous studies, changes in PR1 domain motifs may affect the interaction between PR1 genes and downstream target genes^23,24^. As a result, proteins with changes in sequence structure may be worthy of further investigation into their function and binding specific.ity^36–39^. In the present study, a complete CAP-PR1 domain and PRK14950, PRK10263, and STKt_IRAK domains were identified in 86 PR1 genes in wheat. The gain and loss of domains are components of the divergent powers of gene family expansion. The loss of any PR1 domain was relatively rare in wheat in this study, which indicates that the PR1 family is more conserved in the evolutionary process than other transcription factor families, such as the WRKY family^40^. The present results are consistent with a previous study of *PR1* genes in tomatoes^12^, *Arabidopsis*, and rice^41^, suggesting that the evolution of *PR1* genes is comparable with that in other plants. The number of chromosome segment duplication events was higher than the number of tandem duplication events. This result suggests that chromosome segment duplication contributed to *PR1* gene expansion.

### Functions of the *TaPR1* genes in wheat

In this study, *TaPR1-7A/7B/7D* was identified and isolated from wheat line (L58) leaves. The cluster in the phylogenetic tree might reflect the differentiation of TaPR1 gene function during evolution. *TaPR1-7A/7B/7D* is homologous to CAP (PR1) proteins from different plant species. The hyphal infection area (necrosis symptoms), which is an indicator of Pst fungal expansion ability, was strictly increased in *TaPR1-7A/7B/7D*-silenced wheat. Therefore, we speculate that upregulation of *TaPR1* genes might be an important factor for wheat resistance to Pst. Upstream of the SA pathway is the phenylalanine metabolism pathway (Fig. 3A). Based on these results, we hypothesize that the main role of *TaPR1* in wheat resistance is to enhance the cell walls by promoting the synthesis of lignin in plant cells and the activation of SA pathways to defend against invading foreign microorganisms. This result suggests that *TaPR1-7A/7B/7D* promotes the resistance of wheat to Pst.

The gene structure, conserved domains, and motifs of the upstream region of the transcription start site and the differences in control elements can provide valuable information for studying the gene and its roles under various stress conditions. In this study, eleven different types of cis-regulatory elements in the predicted binding sites of transcription factors (TFs) were identified for the *TaPR1* genes, and they are involved in different biological processes, including response to light, biological stress, defense, and plant hormone responses. Plant TFs play an important role in plant growth and development by binding to specific cis-elements to regulate target gene expression^42^. They control many biological processes, such as metabolism, cell cycle, growth, development, and response to external stimuli. The secondary structure of the *TaPR1* proteins were predicted in this study (Additional file 5). The variations in the protein structures might have effects on activity, stability, interactions, and other properties^43^, which are connected with the diverse functional roles of the PR1 protein in the cell metabolism of wheat. The verification of the secondary structure prediction results provides a preliminary basis for exploring the function of the wheat *TaPR1* gene.

## Methods

### Plant materials and fungal treatments

The wheat cv. Chuanyu 12 (CY12) and the resistant wheat line L58 were used in this study and were obtained from the Chengdu Institute of Biology, Chinese Academy of Sciences (CIB, CAS), which was formalized by Professor Yu Wu’s laboratory. Permission was obtained from the CIB and CAS, and the guidelines of the relevant legislation were followed. CY12 was developed by our research group. With the emergence of new Pst races, CY12 is highly susceptible to the currently predominant stripe rust race. In previous research, L58 [Chuanyu12/04G368/Chuanyu12/Yumai35 F5], an offspring of CY12, demonstrated high resistance to the new Pst-race CYR34 in Gansu and Sichuan. A recombinant inbred line (RIL) population, YZ1/NX188 (198 wheat lines), was used for validation analysis of the molecular markers developed in this study, which were obtained from the Chinese Academy of Agricultural Sciences (CAAS) for linkage analysis. Artificial inoculation was conducted under controlled greenhouse conditions at the Gansu Academy of Agricultural Sciences (GAAS) during the wheat seedling stage. Before inoculation, the waxy layer of wheat leaves (two leaves stage) was removed, and CYR-34 was mixed with 3‰-5‰ Twin-20. Each pot of wheat seedlings was inoculated with 1 mg of CYR34. The invasion temperature of stripe rust was 8–12 ℃, and the incubation temperature of stripe rust was 12–18 ℃ after inoculation. The wheat leaves of CY12 were collected at 0 h (A), 24 h (B), 48 h (C), 72 h (D), and 7 days (E) postinoculation. The wheat leaves of L58 were collected at 0 h (A), 24 h (B), and 7 days (C) postinoculation (each time point had 3 biological replicates). All leaf samples were frozen in liquid nitrogen and stored in a − 80 °C freezer for subsequent RNA isolation and transcriptome sequencing.

### RNA sequencing, Gene Ontology (GO) and KEGG analysis

Total RNA from wheat leaves was extracted using the mirVana miRNA Isolation Kit (Ambion). Purified fragmented mRNA was used to synthesize first-strand cDNA and second-strand cDNA with HiScriptII Q RT SuperMix (Vazyme, Nanjing, China) following the manufacturer’s instructions. The libraries were constructed using the TruSeq Stranded mRNA LT Sample Prep Kit (Illumina, San Diego, CA, USA). Then, 24 cDNA libraries were sequenced on the Illumina sequencing platform (Illumina HiSeq X Ten), and 125 bp/150 bp paired-end reads were generated. Transcriptome sequencing and analysis were conducted by OE Biotech (Shanghai, China). Raw reads containing poly-N and low-quality reads were removed to obtain clean reads. All clean reads were used for subsequent analyses and mapped to the reference genome (IWGSC RefSeq V1.1) using HISAT2^44^. The fragments per kilobase of exon per million mapped fragments (FPKM) value of each gene was calculated, and the read counts of each gene were obtained by htseq-count^45^. The DEGseq R package was used to analyse and identify differentially expressed genes (DEGs) with |log2 FC|> 1 and Q 0.005. GO enrichment and KEGG pathway enrichment analyses of DEGs were performed using R software based on the hypergeometric distribution during CYR34 infection.

### Identification of wheat PR1 genes

The whole PR1 protein sequences in wheat were downloaded from the Ensemble database (http://plants.ensembl.org/Triticum_aestivum/Info/Index). Subsequently, the PR1 protein sequences in *Arabidopsis* (*Arabidopsis thaliana*) and rice (*Oryza sativa* cv. *Nipponbare*)^41^ were used for BLASTP searches (E-value cut-off < 1e^−5^) in the wheat genome database to identify homologous sequences. In addition, predicted proteins from the wheat genome were scanned using HMMER (https://www.ebi.ac.uk/Tools/hmmer/) corresponding to Pfam (http://pfam.xfam.org/) to confirm the presence and integrity of the kinase domain. Furthermore, to verify the existence of the sequence obtained, a BLASTN similarity search was performed on wheat ESTs stored in the National Center for Biotechnology Information (NCBI) database (https://www.ncbi.nlm.nih.gov/). The transcript length, coding exons and genomic location of the putative PR1 gene from wheat were calculated using tools from Ensemble Plants. Using the tools on the ExPASy website (https://web.expasy.org/compute_pi/), the theoretical isoelectric point (PI) and molecular weight (Mw) of the identified PR1 protein were obtained. Subcellular localization of PR1 genes was performed on the UniProt website (https://www.uniprot.org/uniprot/).

### Conserved domain and phylogenetic analysis

Proteins are the basic unit of life activity, and the conserved domains, motifs, and gene structures determine their function. Conserved domains of the PR1 gene family were identified using the Pfam database. The results were confirmed using the NCBI Conservative Domain Database (https://www.ncbi.nlm.nih.gov/cdd/). All full-length amino acid sequences of the PR1 family members were used to identify domain motifs using Multiple EM for Motif Elicitation (MEME) software (Version 5.1.1, https://meme-suite.org/meme/), and the parameters were set as follows: maximum number of different motifs 30; minimum motif width 4; maximum motif width 50. According to the annotation information obtained from the wheat genome, the gene structure information of the PR1 family was obtained, which was visualized using TBtools software (https://github.com/CJ-Chen/TBtools-Manual) ^46^. The PR1 genes were mapped to chromosomal positions by using Phytozome (v12.1) according to the location information of the wheat genome (the wheat genome GFF3 gene annotation file obtained from the wheat database IWGSC v1.1). MCScanX (Multiple Collinearity Scan toolkit) software (https://github.com/wyp1125/MCScanX) was used to analyse PR1 gene duplication events within wheat species and homologous plants^46–48^. Duplicate gene pairs between wheat and rice (*Oryza sativa*) were analysed. The R package “circlize” was used for comprehensive interspecies analysis^49,50^.

The PR1 sequences in *Arabidopsis* and rice were used for phylogenetic analysis in addition to the 86 newly identified PR1 proteins in wheat. Multiple sequence alignment of the obtained PR1 protein sequence was performed using MEGA v7.0 (Molecular Evolutionary Genetics Analysis) software^46^. Based on the results of multiple sequence alignments, the neighbour-joining algorithm was used to construct a phylogenetic tree, and the number of bootstrap replications was set to 1,000^51^. According to the topological structure of the phylogenetic tree, PR1 in wheat, *Arabidopsis*, and rice was divided into different groups.

### Real-time quantitative PCR (qRT‒PCR) analysis

Ten PR1 genes were chosen for qRT‒PCR analysis, with GAPDH serving as the internal control gene. The primers used in the qRT‒PCR analyses were designed based on the sequence of genes identified using Primer 5.0, which are listed in Additional file 1. qRT‒PCR was carried out using the QuantiFast SYBR Green PCR kit (Qiagen, 204,054) according to the manufacturer’s instructions. The experimental conditions were set as follows: 45 cycles at 95 °C for 20 s, 55 °C for 20 s, and 72 °C for 20 s. The exponential expansion period of the amplification curve was used for quantification (Ct values). The levels of all genes were measured using three biological replicates in this study. The mRNA expression level of the genes was calculated with the 2^−ΔΔCt^ method [ΔΔCt = (CT target/Cd − CT actin/Cd − (CT target/control − CT GAPDH/control)]^52^. The correlation between the RNA-seq and qRT‒PCR results was analysed using these values in the R package version 3.1.3 (http://cran.r-project.org/). The normalized relative expression values and FPKM values were calculated using log2 (fold change) measurements.

### Cloning of *TaPR1-7A/7B/7D* and molecular marker development

The *TaPR1* genes (*TraesCS7A02G198800*, *TraesCS7B02G105100*, and *TraesCS7D02G201300*) were identified from the RNA-seq profile, and their expression peaked at 24 hpi after inoculation with Pst-CYR34. Based on the gene sequence from the wheat genome (IWGSC v1.1), homologous sequences of *TaPR1-7A/7B/7D* (*TaPR1-7*) were amplified from the cDNA of L58 by pairs of gene-specific primers for P7A1, P7B1 and P7D3 (Additional file 1), followed by cloning into the pEASY-T1 simple cloning vector (Transgene Biotech, Beijing, China) before sequencing. PCR was performed in a total volume of 25 μL containing 12.5 μL 2 × High-Fidelity Master Mix, 1 μL 10 μM primer F, 1 μL 10 μM primer R, 3 μL template cDNA, and 7.5 μL H_2_O. PCR was carried out as follows: initial denaturation at 98 °C for 2 min, followed by 35 cycles of 98 °C for 10 s, 52 °C for 30 s, and 72 °C for 1 min, followed by a final extension at 72 °C for 5 min and a 4 °C hold. The purified PCR product was sequenced by Tsingke Co., Ltd. (www.tsingke.net). Sequence alignment was used in cloned *TaPR1-7B* in resistant and sensitive wheat to design molecular markers (*TaPR1-7b cibM1*) using the deletion of noncoding regions in two plants. Then, QTL IciMapping was used to validate and map *TaPR1-7b cibM1* in the wheat RIL populations.

### Barley stripe mosaic virus (BSMV)‑mediated gene silencing

BSMV-induced gene silencing was conducted in the present study. BSMV is a positive-sense RNA virus with a tripartite genome consisting of RNAs α, β and γ. In this study, all BSMV construct vectors were obtained from Professor Maoqun Yu’s laboratory at the Chengdu Institute of Biology, Chinese Academy of Sciences. The 266 bp *TaPR1-7* gene fragment was amplified with NheI restriction sites using the primer pair V-TaPR1-7F/R (Additional file 1) and inserted into the original BSMV: γ vector to produce the recombinant plasmid BSMV: TaPR1-7. Using the same method, we constructed the BSMV: TaPDS (185 bp phytoene desaturase gene fragment) expression vector as the positive control. Plasmids BSMV: TaPR1-7, BSMV: TaPDS, and BSMV: γ were linearized followed by transcription and capping in vitro using the RiboMA Large Scale RNA Production System-T7 (Promega, USA) according to the manufacturer’s protocol. BSMV:α, BSMV:β, BSMV:γ, BSMV:TaPDS, and BSMV:TaPR1 were separately mixed at a 1:1:1 ratio and then added to FES buffer for mechanical rubbing inoculation by hand. BSMV: γ was used as the negative control. The secondary leaves of two-week-old wheat seedlings were inoculated separately with equal quantities of BSMV: TaPDS, BSMV: γ and BSMV: TaPR1-7, which were incubated in dark and humid conditions (85.0%) for 24 h. Wheat leaves infected with BSMV TaPR1-7 were sampled at 0, 24, 96 and 192 hpi to measure the transcript levels of *TaPR1-7*. Ten days after BSMV inoculation, the fourth leaves of wheat were infected with Pst-CYR34, and necrotic areas of wheat were measured. The symptoms present on the wheat leaves were photographed at 14 days after inoculation with Pst-CYR34. These experiments were repeated at least three times. All primers used for gene cloning, vector construction, and qRT‒PCR assays are listed in Additional file 1.

### Ethical approval and consent to participate

The materials used in this article did not involve disputes. The collection of wheat materials (Chuanyu 12 and L58) used in the experiment described in this article was carried out with the permission of Chengdu Institute of Biology, Chinese Academy of Sciences (CIB, CAS), and Professor Yu Wu from the Agricultural Center authenticated the plant. We confirm that all experiments were carried out in accordance with the relevant guidelines and regulations, and the datasets used and/or analysed during the current study are available from the corresponding author on reasonable request.

## Conclusions

A comprehensive analysis of the *TaPR1* gene family of wheat was conducted in this study. A total of 86 *TaPR1* genes were identified based on the wheat genome. We identified differences related to gene structure, conserved domains, motifs, and cis-regulatory elements in the identified *TaPR1* genes. The gene expression profiles and phylogenetic analysis of *TaPR1* genes in several different plants and under different stress conditions might provide valuable clues for studying the evolutionary characteristics of wheat *TaPR1* genes. The *TaPR1* gene plays an important role in wheat disease resistance. During the process of pst-CYR34 infection in wheat, most of the *TaPR1* genes (containing the CAP domain) were highly expressed after 24 hpi. The *TaPR1* gene expression pattern due to the biotic stress induced by rust disease will allow us to understand the interaction of plant pathogens more widely at the molecular level. In addition, the function of *TaPR1-1A/7B/7D* was further analysed by BSMV-VIGS and polymorphism typing by molecular markers. The results of phylogenetic analysis and gene expression analyses will also provide the basis for gene function analyses. Subsequent mining and identification of the *TaPR1* gene may provide new insights into the mode of action that promotes the response of wheat to auxin during rust infection. In conclusion, our results provide valuable data for exploring the function of the *TaPR1* gene in response to stripe rust in wheat.

## Supplementary Information

Supplementary Information 1.

Supplementary Information 2.

Supplementary Information 3.

Supplementary Information 4.

Supplementary Information 5.

Supplementary Information 6.

Supplementary Information 7.

Supplementary Information 8.

## Data Availability

The datasets analysed during the current study are available in the NCBI BioProject repository [PRJNA737275].

## Abbreviations

PR1: Pathogenesis-related protein-1
Pst: *Puccinia striiformis* F. sp. *Tritici*
SAR: Systemic acquired resistance
SA: Salicylic acid
CY12: Chuanyu 12
DEGs: Differentially expressed genes
PI: Theoretical isoelectric point
Mw: Molecular weight
MEME: Multiple EM for motif elicitation
BSMV: Barley stripe mosaic virus
ATBP-1: AT-rich DNA binding protein
CAP: Cysteine-rich secretory proteins
TF: Transcription factors
