# Supplementary material for: Characterization and functional analyses of wheat TaPR1 genes in response to stripe rust fungal infection
Source: Sci Rep. 2023 Feb 27;13:3362. doi: 10.1038/s41598-023-30456-8 (PMC9971213; doi:10.1038/s41598-023-30456-8)
Supplement: Supplementary file 1 — Supplementary Information 1. [file 41598_2023_30456_MOESM1_ESM.pdf]

Additional file 1. List of primer sequences used by qRT-PCR and PCR

Gene_ID	Forward primer 5'-3'
TraesCS3A02G477300	ATATGGGTGACCACCACCAC
TraesCS5A02G183300	GAGTAATGGAGACGCCCAAG
TraesCS5B02G181500	GCTACGGCCTACACATACGC
TraesCS7A02G198800	GGCATCTTCCAAGAGCAGTC
TraesCS7A02G198900	GAGCAGTCTTGCAATGTTCG
TraesCS7B02G105100	GTCTTGCAATGTTCGCACTG
TraesCS7B02G105300	ATACCGGTCAAATTCCAGCA
TraesCS7D02G161200	GCGGGAATATCATTGGACAG
TraesCS7D02G201300	TCCATCTCGAGCCACCTACT
TraesCS7D02G201400	CGACTGCATGAGGAAGGAAG
P7A1	ATCGATCTCGAGCCACC
P7B1	CGATCTCGAGTCACCTAC
P7D3	ATCCATCTCGAGCCACCTAC
TaPR1-7	TACGCTAGCCGTGGCCAGGTTCGCGCAGGAC
TaPDS	TACGCTAGCCTGGATGAAAAAGCAGGGTGTTCC
TaPR1-7B1-cibM1	TAAGCAGCACGATCACGAAC

in this study.

Reverse primer 5'-3'	Product (bp)	TM (℃)
AGGGCTGACCAAGACTGCTA	103	60
GAGGCGAGAGGTAGTCCTGA	108	59.5
TTGGGCGTCTCCATTACTTC	91	60
GAAGTCCTGCGGTGTGTTCT	94	60
TTGTGCAGGTTGACGAAGTC	96	60
GTGCAGGTTGACGAAGTCCT	94	60
CAGTGCGAACATTGCAAGAC	94	60
TGCGATTAGGGACGAAAGAC	97	60
ACCACGGCCATGACTATGAC	87	60
TGCTTGGAGTCAAGACAACG	99	60
CTCATTACATTCACACGC	~800	55
TTCAAATTTCGTTTGAAAGTT	~800	55
CACGCTCCACAGAGCCGCATGG	~800	55
TACGCTAGCGGTGATGAAGACGCCCCGGTTCC	266	60
TACGCTAGCCTACTTTCAGGAGGATTACCATCC	184	60
TTGTGCAGGTTGACGAAGTC	1650	52
