# Supplementary material for: Characterization and functional analyses of wheat TaPR1 genes in response to stripe rust fungal infection
Source: Sci Rep. 2023 Feb 27;13:3362. doi: 10.1038/s41598-023-30456-8 (PMC9971213; doi:10.1038/s41598-023-30456-8)
Supplement: Supplementary file 2 — Supplementary Information 2. [file 41598_2023_30456_MOESM2_ESM.pdf]

Additional file 2. Expression analysis of enrichment genes in wheat PR1 gene family and its

Gene_ID	CY12-A1	CY12-A2	CY12-A3	CY12-B1	CY12-B2	CY12-B3	CY12-C1
TraesCS1A02G443800	0	0	0	0	0	0	0
TraesCS1A02G444000	0	0	0	0	0	0	0
TraesCS1B02G478300	0	0	0	0	0	0	0
TraesCS1B02G478500	0	0	0	0	0	0	0
TraesCS1D02G452000	0	0	0	0	0	0	0
TraesCS2A02G439600	0	0	0	0	0	0	0
TraesCS2A02G439700	0	0	0	0	0	0	0
TraesCS2A02G441400	0	0	0	0	0	0	0
TraesCS2B02G403600	0	0	0	0	0	0	0
TraesCS2B02G403700	0	0	0	0	0	0	0
TraesCS2B02G459500	0	0	0	0	0	0	0
TraesCS2B02G459600	0	0	0	0	0	0	0
TraesCS2B02G459700	0	0	0	0	0	0	0
TraesCS2D02G382900	0	0	0	0	0	0	0
TraesCS2D02G436900	0	0	0	0	0	0	0
TraesCS2D02G437000	0	0	0	0	0	0	0
TraesCS2D02G437100	0	0	0	0	0	0	0
TraesCS2D02G437200	0	0	0	0	0	0	0
TraesCS2D02G437300	0	0	0	0	0	0	0
TraesCS2D02G437400	0	0	0	0	0	0	0
TraesCS3A02G477300	0.23703	0.70263	0.1917	3.4432	2.26129	3.31347	1.01125
TraesCS3A02G525700	0	0	0	0	0	0	0
TraesCS3D02G472000	0	0	0	0.09224	0.08135	0	0.09375
TraesCS3D02G530800	0	0	0	0	0	0	0
TraesCS4A02G251300	0	0	0	0	0	0	0.05282
TraesCS4B02G063600	0	0	0	0	0	0	0
TraesCS4D02G062500	0	0	0	0	0	0	0
TraesCS5A02G012900	0	0	0	0	0	0	0
TraesCS5A02G059000	0	0	0	0	0	0	0
TraesCS5A02G183300	1.83915	2.87867	4.60624	23.5313	51.4697	28.0276	132.123
TraesCS5A02G439700	0	0	0	0	0	0	0
TraesCS5A02G439800	0	0	0	0	0	0	2.84868
TraesCS5A02G439900	0	0	0	0	0	0	0
TraesCS5A02G440000	0	0	0	0	0	0	0
TraesCS5B02G011200	0	0	0	0	0	0	0
TraesCS5B02G066300	0	0	0	0	0	0	0
TraesCS5B02G181500	0	0.13717	2.99289	14.383	9.45464	10.0923	43.8186
TraesCS5B02G442600	0	0.84139	0	0	0	0	0.17051
TraesCS5B02G442700	0	0	0	0	0.45756	0	1.31434
TraesCS5B02G442800	0	0	0	0.17184	0	0	0
TraesCS5B02G442900	0	0	0	0.47524	0.47796	0.63872	0.38786
TraesCS5B02G443000	0	0	0	0	0	0	0
TraesCS5B02G443100	0	0	0	0.9717	0.09658	0	0.09377
TraesCS5B02G443200	0	0	0	0	0	0	0
TraesCS5B02G443300	0	0	0	0	0	0	0
TraesCS5B02G443400	0	0	0	0	0	0	0
TraesCS5B02G443500	0	0	0	0	0	0	0
TraesCS5B02G443600	0	0	0	0	0	0	0
TraesCS5B02G443700	0	0	0	0	0	0	0
TraesCS5B02G443800	0	0	0	0	0.35376	0	0
TraesCS5D02G446800	0	0	0	0	0	0	0.08748
TraesCS5D02G446900	0	0	0	0	0	0	0.22639
TraesCS5D02G447000	0	0	0	0	0	0	0
TraesCS5D02G447100	0	0	0	0	0	0	0
TraesCS6A02G345000	0	0	0	0	0	0	0
TraesCS6A02G345100	0	0	0	0	0	0	0

TraesCS6A02G345200	0	0	0	0	0	0	0
TraesCS6A02G346300	0	0	0	0	0	0	0
TraesCS6B02G377700	0	0	0	0	0	0	0
TraesCS6B02G377800	0	0	0	0	0	0	0
TraesCS6B02G378000	0	0	0	0	0	0	0
TraesCS6B02G379800	0	0	0	0	0	0	0
TraesCS6D02G327500	0	0	0	0	0	0	0
TraesCS6D02G327600	0	0	0	0	0	0	0
TraesCS6D02G327700	0	0	0	0	0	0	0
TraesCS6D02G329200	0	0	0	0	0	0	0
TraesCS7A02G152200	0	0	0	0	0	0	0
TraesCS7A02G198800	2.35082	1.9164	1.08257	4.71205	0.98057	17.9426	0.57092
TraesCS7A02G198900	224.567	190.205	225.38	207.246	182.724	258.109	119.107
TraesCS7A02G565100	0	0	0	0	0	0	0
TraesCS7B02G056100	0	0	0	0	0	0	0
TraesCS7B02G104900	0.05524	0.29524	0	0.06455	0.33386	0.32761	0
TraesCS7B02G105000	0	0	0	0	0	0	0
TraesCS7B02G105100	0	0	0.05796	0.5535	0.76171	0.93631	3.1605
TraesCS7B02G105200	0	0	0	0	0	0	0
TraesCS7B02G105300	59.1308	33.7649	31.4052	95.3132	53.9073	103.471	66.1834
TraesCS7D02G099600	0	0	0	0	0	0	0
TraesCS7D02G153900	0	0	0	0	0	0	0
TraesCS7D02G161200	0	0.8541	2.34836	5.53843	27.3021	4.5091	63.1369
TraesCS7D02G201300	52.9231	46.0111	58.4321	86.8574	71.3152	142.549	11.5203
TraesCS7D02G201400	266.765	258.285	339.525	323.825	253.122	523.744	194.103
TraesCSU02G076600	0	0	0	0	0	0	0
TraesCSU02G095300	0	0	0	0	0	0	0
TraesCSU02G202900	0	0	0	0	0	0	0
TraesCSU02G226400	0	0	0	0	0	0	0
TraesCSU02G233000	0	0	0	0	0	0	0

NPR1

**Gene-ID**	**CY12-0h**	**CY12-24l**	**CY12-48l**	**CY12-72l**	**CY12-7D**	**L58-0h**	**L58-24h**
TraesCS3A02G105400	2.56976	7.84072	2.13405	1.4873	1.51598	1.11381	6.03464
TraesCS3B02G123800	5.46675	24.8848	3.64298	1.60438	3.49301	3.47512	9.27498
TraesCS3D02G107500	2.3931	18.2646	1.35473	0.87856	1.58266	1.04687	8.54454

TGA

**Gene-ID**	**CY12-0h**	**CY12-24l**	**CY12-48l**	**CY12-72l**	**CY12-7D**	**L58-0h**	**L58-24h**
TraesCS3D02G327600	0.00665	0	0	0.04692	0.01267	0.02083	0.11228
TraesCS4A02G126300	5.48591	18.0852	3.97354	4.48205	3.04559	4.65627	17.3257
TraesCS4B02G178600	7.35177	21.1007	6.09393	6.02176	4.23216	5.72837	18.5743

pathway							
**Gene_ID**	**CY12-C2**	**CY12-C3**	**CY12-D1**	**CY12-D2**	**CY12-D3**	**CY12-E1**	**CY12-E2**
TraesCS1A02G443800	0	0	0	0	0	0	0
TraesCS1A02G444000	0	0	0	0	0	0	0
TraesCS1B02G478300	0	0	0	0	0	0	0
TraesCS1B02G478500	0	0	0	0	0	0	0
TraesCS1D02G452000	0	0	0	0	0	0	0
TraesCS2A02G439600	0	0	0	0	0	0	0
TraesCS2A02G439700	0	0	0	0	0	0	0
TraesCS2A02G441400	0	0	0	0	0	0	0
TraesCS2B02G403600	0	0	0	0	0	0	0
TraesCS2B02G403700	0	0	0	0	0	0	0
TraesCS2B02G459500	0	0	0	0	0	0	0
TraesCS2B02G459600	0	0	0	0	0	0	0
TraesCS2B02G459700	0	0	0	0	0	0	0
TraesCS2D02G382900	0	0	0	0	0	0	0
TraesCS2D02G436900	0	0	0	0	0	0	0
TraesCS2D02G437000	0	0	0	0	0	0	0
TraesCS2D02G437100	0	0	0	0	0	0	0
TraesCS2D02G437200	0	0	0	0	0	0	0
TraesCS2D02G437300	0	0	0	0	0	0	0
TraesCS2D02G437400	0	0	0	0	0	0	0
TraesCS3A02G477300	2.59087	1.27385	1.48767	2.17627	1.18869	7.36465	5.35799
TraesCS3A02G525700	0	0	0	0	0	0	0
TraesCS3D02G472000	0.28761	0.04795	0.21819	0.18589	0.22076	0.81732	0.86364
TraesCS3D02G530800	0	0	0	0	0	0	0
TraesCS4A02G251300	0	0	0	0	0.45487	0	0
TraesCS4B02G063600	0	0	0	0	0	0	0
TraesCS4D02G062500	0	0	0.08012	0	0	0	0
TraesCS5A02G012900	0	0	0	0	0	0	0
TraesCS5A02G059000	0	0	0	0	0	0	0
TraesCS5A02G183300	199.836	42.8972	154.354	150.595	165.393	252.965	333.061
TraesCS5A02G439700	0	0	0.55514	0.55031	0.09329	2.27875	6.97774
TraesCS5A02G439800	1.552	0.51087	0.33867	0.85647	0.51575	8.65636	10.6152
TraesCS5A02G439900	0	0	0	0	0	0	0
TraesCS5A02G440000	0	0	0	0	0	0	0
TraesCS5B02G011200	0	0	0	0	0	0	0
TraesCS5B02G066300	0	0	0	0	0	0	0
TraesCS5B02G181500	64.8637	21.2335	85.1961	51.6147	81.4764	105.583	165.929
TraesCS5B02G442600	0	0.51741	1.37265	3.48388	2.09336	5.43264	9.89247
TraesCS5B02G442700	1.03268	0.28609	5.29594	0.87284	4.04016	13.6035	18.455
TraesCS5B02G442800	0	0	0	0	0	0	0
TraesCS5B02G442900	0	0.45937	0	0	0	0	0
TraesCS5B02G443000	0	0	0	0	0	0	0
TraesCS5B02G443100	0.18777	0	0	0.09269	0	0	0
TraesCS5B02G443200	0	0	0	0	0	0	0
TraesCS5B02G443300	0	0	0	0	0	0	0
TraesCS5B02G443400	0	0	0	0	0	0	0
TraesCS5B02G443500	0	0	0	0	0	0	0
TraesCS5B02G443600	0	0	0	0	0	0	0
TraesCS5B02G443700	0	0	0	0	0	0	0
TraesCS5B02G443800	0	0.16883	0	0	0	0	0
TraesCS5D02G446800	0	0	1.57932	0.26694	0.44817	10.5675	16.0352
TraesCS5D02G446900	0	0	1.13042	1.2077	0.22749	6.48389	14.7456
TraesCS5D02G447000	0	0	0	0	0	0	0.11289
TraesCS5D02G447100	0	0	0	0	0	0	0
TraesCS6A02G345000	0	0	0	0	0	0	0
TraesCS6A02G345100	0	0	0	0	0	0	0

TraesCS6A02G345200	0	0	0	0	0	0	0
TraesCS6A02G346300	0	0	0	0	0	0	0
TraesCS6B02G377700	0	0	0	0	0	0	0
TraesCS6B02G377800	0	0	0	0	0	0	0
TraesCS6B02G378000	0	0	0	0	0	0	0
TraesCS6B02G379800	0	0	0	0	0	0	0
TraesCS6D02G327500	0	0	0	0	0	0	0
TraesCS6D02G327600	0	0	0	0	0	0	0
TraesCS6D02G327700	0	0	0	0	0	0	0
TraesCS6D02G329200	0	0	0	0	0	0	0
TraesCS7A02G152200	0	0	0	0	0	0	0
TraesCS7A02G198800	0.3891	0.19917	0.39661	1.14026	0.5263	0.32662	0
TraesCS7A02G198900	108.242	133.555	144.029	139.935	204.515	64.3894	59.7547
TraesCS7A02G565100	0	0	0	0	0	0	0
TraesCS7B02G056100	0	0	0	0	0	0	0
TraesCS7B02G104900	0	0.63615	0.3169	0.18088	0.12641	0.37284	0.17599
TraesCS7B02G105000	0	0	0	0	0	0	0
TraesCS7B02G105100	2.49264	1.62818	2.58805	1.95734	2.87373	3.79151	2.18204
TraesCS7B02G105200	0	0	0	0.14796	0	0	0
TraesCS7B02G105300	53.0486	70.0732	60.798	55.3872	91.4111	44.5476	10.748
TraesCS7D02G099600	0	0	0	0	0	0	0
TraesCS7D02G153900	0	0	0	0	0	0	0
TraesCS7D02G161200	105.627	12.0874	136.146	109.02	112.83	226.75	423.659
TraesCS7D02G201300	6.53579	9.08785	15.3599	17.0928	23.5832	7.83694	7.3397
TraesCS7D02G201400	159.975	175.256	205.876	175.512	321.672	134.346	40.7026
TraesCSU02G076600	0	0	0	0	0	0	0
TraesCSU02G095300	0	0	0	0	0	0	0
TraesCSU02G202900	0	0	0	0	0	0	0
TraesCSU02G226400	0	0	0	0	0	0	0
TraesCSU02G233000	0	0	0	0	0	0	0

NPR1

**Gene-ID**	**L58-7D**
TraesCS3A02G105400	0.45397
TraesCS3B02G123800	1.62075
TraesCS3D02G107500	0.61575

TGA

**Gene-ID**	**L58-7D**
TraesCS3D02G327600	0.02062
TraesCS4A02G126300	4.2443
TraesCS4B02G178600	4.32234

Gene_ID	CY12-E3	L58-A1	L58-A2	L58-A3	L58-B1	L58-B2	L58-B3
TraesCS1A02G443800	0	0	0	0	0	0	0
TraesCS1A02G444000	0	0	0	0	0	0	0
TraesCS1B02G478300	0	0	0	0	0	0	0
TraesCS1B02G478500	0	0	0	0	0	0	0
TraesCS1D02G452000	0	0	0	0	0	0	0
TraesCS2A02G439600	0	0	0	0	0	0	0
TraesCS2A02G439700	0	0	0	0	0	0	0
TraesCS2A02G441400	0	0	0	0	0	0	0
TraesCS2B02G403600	0	0	0	0	0	0	0
TraesCS2B02G403700	0	0	0	0	0	0	0
TraesCS2B02G459500	0	0	0	0	0	0	0
TraesCS2B02G459600	0	0	0	0	0	0	0
TraesCS2B02G459700	0	0	0	0	0	0	0
TraesCS2D02G382900	0	0	0	0	0	0	0
TraesCS2D02G436900	0	0	0	0	0	0	0
TraesCS2D02G437000	0	0	0	0	0	0	0
TraesCS2D02G437100	0	0	0	0	0	0	0
TraesCS2D02G437200	0	0	0	0	0	0	0
TraesCS2D02G437300	0	0	0	0	0	0	0
TraesCS2D02G437400	0	0	0	0	0	0	0
TraesCS3A02G477300	0.57396	0.20785	0.17567	0.05901	4.608	5.21105	4.97074
TraesCS3A02G525700	0	0	0	0	0	0	0
TraesCS3D02G472000	0	0	0	0.04905	0.07939	0.03097	0
TraesCS3D02G530800	0	0	0	0	0	0	0
TraesCS4A02G251300	0	0	0	0	0	0	0
TraesCS4B02G063600	0	0	0	0	0	0	0
TraesCS4D02G062500	0	0	0	0	0	0	0
TraesCS5A02G012900	0	0	0	0	0	0	0
TraesCS5A02G059000	0	0	0	0	0	0	0
TraesCS5A02G183300	13.0112	86.0993	18.6975	36.5499	163.984	179.161	104.914
TraesCS5A02G439700	0	0	0	0	0	0	0
TraesCS5A02G439800	0	0	0	0	0	0	0
TraesCS5A02G439900	0	0	0	0	0	0	0
TraesCS5A02G440000	0	0	0	0	0	0	0
TraesCS5B02G011200	0	0	0	0	0	0	0
TraesCS5B02G066300	0	0	0	0	0	0	0
TraesCS5B02G181500	1.7964	29.8102	4.0435	9.37502	88.2195	81.022	33.5012
TraesCS5B02G442600	0	0	0	0	0	0	0
TraesCS5B02G442700	0	0	0	0	0.29863	1.42181	0
TraesCS5B02G442800	0	0	0	0	0	0	0
TraesCS5B02G442900	0	0	0	0.07185	1.38567	0.14946	1.30595
TraesCS5B02G443000	0	0	0	0	0	0	0
TraesCS5B02G443100	0	0	0	0	0.18689	0.08966	0.92453
TraesCS5B02G443200	0	0	0	0	0	0	0
TraesCS5B02G443300	0	0	0	0	0	0	0
TraesCS5B02G443400	0	0	0	0	0	0	0
TraesCS5B02G443500	0	0	0	0	0	0	0
TraesCS5B02G443600	0	0	0	0	0	0	0
TraesCS5B02G443700	0	0	0	0	0	0	0
TraesCS5B02G443800	0	0	0	0	0.1748	0	0
TraesCS5D02G446800	0	0	0	0	0	0	0
TraesCS5D02G446900	0.44018	0.07438	0	0	0	0	0
TraesCS5D02G447000	0	0	0	0	0.11801	0	0
TraesCS5D02G447100	0	0	0	0	0	0	0
TraesCS6A02G345000	0	0	0	0	0	0	0
TraesCS6A02G345100	0	0	0	0	0	0	0

TraesCS6A02G345200	0	0	0	0	0	0	0
TraesCS6A02G346300	0	0	0	0	0	0	0
TraesCS6B02G377700	0	0	0	0	0	0	0
TraesCS6B02G377800	0	0	0	0	0	0	0
TraesCS6B02G378000	0	0	0	0	0	0	0
TraesCS6B02G379800	0	0	0	0	0	0	0
TraesCS6D02G327500	0	0	0	0	0	0	0
TraesCS6D02G327600	0	0	0	0	0	0	0
TraesCS6D02G327700	0	0	0	0	0	0	0
TraesCS6D02G329200	0	0	0	0	0	0	0
TraesCS7A02G152200	0	0	0	0	0	0	0
TraesCS7A02G198800	0	0.53109	1.00752	1.94885	11.9182	2.48422	42.1356
TraesCS7A02G198900	24.3963	142.71	129.226	145.424	312.008	275.189	625.01
TraesCS7A02G565100	0	0	0	0	0	0	0
TraesCS7B02G056100	0	0	0	0	0	0	0
TraesCS7B02G104900	0	0	0	0	0.31715	0.91424	3.09918
TraesCS7B02G105000	0	0	0	0	0.05963	0	0
TraesCS7B02G105100	0.23516	0.59892	0.36422	0.51339	1.51232	5.973	7.91472
TraesCS7B02G105200	0.13664	0	0	0	0	0	0
TraesCS7B02G105300	8.168	8.03925	9.36118	6.76099	176.168	73.0324	299.396
TraesCS7D02G099600	0	0	0	0	0	0	0
TraesCS7D02G153900	0	0	0	0	0	0	0
TraesCS7D02G161200	7.41903	21.4363	2.51496	12.7995	53.0087	154.3	28.8274
TraesCS7D02G201300	1.74215	33.2249	33.0302	33.4504	155.23	81.314	253.826
TraesCS7D02G201400	9.14096	137.202	104.042	170.64	662.005	348.581	1101.69
TraesCSU02G076600	0	0	0	0	0	0	0
TraesCSU02G095300	0	0	0	0	0	0	0
TraesCSU02G202900	0	0	0	0	0	0	0
TraesCSU02G226400	0	0	0	0	0	0	0.68087
TraesCSU02G233000	0	0	0	0	0	0	0

Gene_ID	L58-E1	L58-E2	L58-E3	
TraesCS1A02G443800	0	0	0	hypothetical protein BRADI_2g14255, partial [Brachypodium
TraesCS1A02G444000	0	0	0	Pathogenesis-related protein 1A [Aegilops tauschii]
TraesCS1B02G478300	0	0	0	hypothetical protein BRADI_2g14255, partial [Brachypodium
TraesCS1B02G478500	0	0	0	Pathogenesis-related protein 1A [Aegilops tauschii]
TraesCS1D02G452000	0	0	0	Pathogenesis-related protein 1A [Aegilops tauschii]
TraesCS2A02G439600	0	0	0	STS14 protein [Triticum urartu]
TraesCS2A02G439700	0	0	0	STS14 protein [Triticum urartu]
TraesCS2A02G441400	0	0	0	Pathogenesis-related protein 1C [Aegilops tauschii]
TraesCS2B02G403600	0	0	0	Pathogenesis-related protein 1A [Triticum urartu]
TraesCS2B02G403700	0	0	0	Pathogenesis-related protein 1C [Aegilops tauschii]
TraesCS2B02G459500	0	0	0	Pathogenesis-related protein PRMS [Aegilops tauschii]
TraesCS2B02G459600	0	0	0	STS14 protein [Triticum urartu]
TraesCS2B02G459700	0	0	0	Pathogenesis-related protein 1C [Aegilops tauschii]
TraesCS2D02G382900	0	0	0	Pathogenesis-related protein 1C [Aegilops tauschii]
TraesCS2D02G436900	0	0	0	Pathogenesis-related protein PRMS [Aegilops tauschii]
TraesCS2D02G437000	0	0	0	Pathogenesis-related protein PRMS [Aegilops tauschii]
TraesCS2D02G437100	0	0	0	Pathogenesis-related protein PRMS [Aegilops tauschii]
TraesCS2D02G437200	0	0	0	STS14 protein [Triticum urartu]
TraesCS2D02G437300	0	0	0	Pathogenesis-related protein PRMS [Aegilops tauschii]
TraesCS2D02G437400	0	0	0	Pathogenesis-related protein 1C [Aegilops tauschii]
TraesCS3A02G477300	1.45606	1.55801	2.70813	Cysteine-rich receptor-like protein kinase 29 [Aegilops taus
TraesCS3A02G525700	0	0	0	pathogenesis-related protein 1 [Triticum aestivum]
TraesCS3D02G472000	0.20145	0.39135	0.17797	Cysteine-rich receptor-like protein kinase 29 [Aegilops taus
TraesCS3D02G530800	0	0	0	pathogenesis-related protein 1-12 [Triticum aestivum]
TraesCS4A02G251300	0	0.33012	0	predicted protein [Hordeum vulgare subsp. vulgare]
TraesCS4B02G063600	0	0	0	predicted protein [Hordeum vulgare subsp. vulgare]
TraesCS4D02G062500	0	0	0	predicted protein [Hordeum vulgare subsp. vulgare]
TraesCS5A02G012900	0	0	0	Pathogenesis-related protein PR-1 [Triticum urartu]
TraesCS5A02G059000	0	0	0	pathogenesis-related protein 1-13 [Triticum aestivum]
TraesCS5A02G183300	101.9	69.615	151.154	pathogenesis-related protein 1-2 [Triticum aestivum]
TraesCS5A02G439700	1.1263	0.8118	0.93452	pathogenesis-related protein 1-9 [Triticum aestivum]
TraesCS5A02G439800	0.34023	0.51296	1.96778	pathogenesis-related protein 1-19 [Triticum aestivum]
TraesCS5A02G439900	0	0	0	Pathogenesis-related protein PRMS [Triticum urartu]
TraesCS5A02G440000	0	0	0	Pathogenesis-related protein 1 [Triticum urartu]
TraesCS5B02G011200	0	0	0	Pathogenesis-related protein PR-1 [Triticum urartu]
TraesCS5B02G066300	0	0	0	pathogenesis-related protein 1-22 [Triticum aestivum]
TraesCS5B02G181500	33.9463	34.8211	65.0259	pathogenisis-related protein 1.1 [Triticum aestivum]
TraesCS5B02G442600	0.17361	0	2.1236	Pathogenesis-related protein PRMS [Aegilops tauschii]
TraesCS5B02G442700	1.58435	2.2564	4.01988	pathogenesis-related protein 1-16 [Triticum aestivum]
TraesCS5B02G442800	0	0	0	pathogenesis-related protein 1-8 [Triticum aestivum]
TraesCS5B02G442900	0	0.07488	0	pathogenesis-related protein 1-6 [Triticum aestivum]
TraesCS5B02G443000	0	0	0	pathogenesis-related protein 1-6 [Triticum aestivum]
TraesCS5B02G443100	0	0	0	pathogenesis-related protein 1-6 [Triticum aestivum]
TraesCS5B02G443200	0	0	0	pathogenesis-related protein 1-7 [Triticum aestivum]
TraesCS5B02G443300	0	0	0	pathogenesis-related protein 1-7 [Triticum aestivum]
TraesCS5B02G443400	0	0	0	pathogenesis-related protein 1-7 [Triticum aestivum]
TraesCS5B02G443500	0	0	0	pathogenesis-related protein 1-7 [Triticum aestivum]
TraesCS5B02G443600	0	0	0	pathogenesis-related protein 1-7 [Triticum aestivum]
TraesCS5B02G443700	0	0	0	pathogenesis-related protein 1-7 [Triticum aestivum]
TraesCS5B02G443800	0	0	0	pathogenesis-related protein 1-7 [Triticum aestivum]
TraesCS5D02G446800	1.35979	0.69378	1.5324	Pathogenesis-related protein PRMS [Aegilops tauschii]
TraesCS5D02G446900	0.65803	0.73591	2.35067	pathogenesis-related protein 1-16 [Triticum aestivum]
TraesCS5D02G447000	0	0	0	pathogenesis-related protein 1-8 [Triticum aestivum]
TraesCS5D02G447100	0	0	0	Pathogenesis-related protein 1 [Triticum urartu]
TraesCS6A02G345000	0	0	0	hypothetical protein BRADI_3g53637 [Brachypodium distach
TraesCS6A02G345100	0	0	0	Pathogenesis-related protein PR-1 [Aegilops tauschii]

TraesCS6A02G345200	0	0	1.06961	PREDICTED: pathogenesis-related protein PRB1-3 [Oryza sa
TraesCS6A02G346300	0	0	0	predicted protein [Hordeum vulgare subsp. vulgare]
TraesCS6B02G377700	0	0	0	hypothetical protein BRADI_3g53637 [Brachypodium distach
TraesCS6B02G377800	0	0	0	Pathogenesis-related protein PR-1 [Aegilops tauschii]
TraesCS6B02G378000	0	0	0	PREDICTED: pathogenesis-related protein PRB1-3 [Oryza sa
TraesCS6B02G379800	0	0	0	predicted protein [Hordeum vulgare subsp. vulgare]
TraesCS6D02G327500	0	0	0	hypothetical protein BRADI_3g53637 [Brachypodium distach
TraesCS6D02G327600	0	0	0	Pathogenesis-related protein PR-1 [Aegilops tauschii]
TraesCS6D02G327700	0	0	0	PREDICTED: pathogenesis-related protein PRB1-3 [Oryza sa
TraesCS6D02G329200	0	0	0	Pathogenesis-related protein PR-1 [Aegilops tauschii]
TraesCS7A02G152200	0	0	0	Pathogenesis-related protein 1 [Aegilops tauschii]
TraesCS7A02G198800	0.25109	0	0.3591	pathogenesis-related protein 1-14 [Triticum aestivum]
TraesCS7A02G198900	52.529	55.1282	59.1347	pathogenesis-related protein 1-17 [Triticum aestivum]
TraesCS7A02G565100	0	0	0	pathogenesis-related protein 1-15 [Triticum aestivum]
TraesCS7B02G056100	0	0	0	Pathogenesis-related protein 1 [Aegilops tauschii]
TraesCS7B02G104900	0	0	0	pathogenesis-related protein 1-21 [Triticum aestivum]
TraesCS7B02G105000	0	0	0	pathogenesis-related protein 1-14 [Triticum aestivum]
TraesCS7B02G105100	1.49111	0.69063	1.34981	pathogenesis-related protein 1-18 [Triticum aestivum]
TraesCS7B02G105200	0.28561	0.3494	0	pathogenesis-related protein 1-18 [Triticum aestivum]
TraesCS7B02G105300	17.8909	8.91926	12.145	pathogenesis-related protein 1-17 [Triticum aestivum]
TraesCS7D02G099600	0	0	0	pathogenesis-related protein 1-11 [Triticum aestivum]
TraesCS7D02G153900	0	0	0	Pathogenesis-related protein 1 [Aegilops tauschii]
TraesCS7D02G161200	114.747	100.058	186.129	pathogenesis-related protein 1 [Triticum aestivum]
TraesCS7D02G201300	8.07378	4.46697	10.8424	pathogenesis related protein-1.2 [Triticum aestivum]
TraesCS7D02G201400	53.5796	44.951	61.9541	pathogenisis-related protein 1.2 [Triticum aestivum]
TraesCSU02G076600	0	0	0	pathogenesis-related protein 1-8 [Triticum aestivum]
TraesCSU02G095300	0	0	0	pathogenesis-related protein 1-10 [Triticum aestivum]
TraesCSU02G202900	0	0	0	pathogenesis-related protein 1-8 [Triticum aestivum]
TraesCSU02G226400	0	0	0	pathogenesis-related protein 1-8 [Triticum aestivum]
TraesCSU02G233000	0	0	0	pathogenesis-related protein 1-8 [Triticum aestivum]
