# Supplementary material for: Characterization and functional analyses of wheat TaPR1 genes in response to stripe rust fungal infection
Source: Sci Rep. 2023 Feb 27;13:3362. doi: 10.1038/s41598-023-30456-8 (PMC9971213; doi:10.1038/s41598-023-30456-8)
Supplement: Supplementary file 3 — Supplementary Information 3. [file 41598_2023_30456_MOESM3_ESM.pdf]

Additional file 3. Domain prediction information of PR1 genes in wheat.

#Batch CD-search tool	NIH/NLM/NCBI				
#cdsid	QM3-qcdsearch-733E1523AE8359E-37960943D4222AD5				
#datatype	hitsConcise Results				
#status	0				
#Start time	2020-06-20T01:3	Run time	0:00:01:23		
#status	success				

Query	Hit type	PSSM-ID	From	To	E-Value
Q#1 - >TraesCS1A02G443800.1	superfamily	381818	146	278	2.07E-23
Q#2 - >TraesCS1A02G444000.1	specific	349400	110	242	9.81E-64
Q#3 - >TraesCS1B02G478300.1	superfamily	381818	74	202	3.42E-26
Q#4 - >TraesCS1B02G478500.1	specific	349400	111	243	1.07E-63
Q#5 - >TraesCS1D02G452000.1	specific	349400	111	243	8.07E-64
Q#6 - >TraesCS2A02G439600.1	superfamily	381818	42	157	1.04E-33
Q#7 - >TraesCS2A02G439700.1	superfamily	381818	47	181	1.91E-40
Q#8 - >TraesCS2A02G441400.1	superfamily	381818	47	183	2.64E-50
Q#9 - >TraesCS2B02G403600.1	superfamily	381818	54	191	2.26E-54
Q#10 - >TraesCS2B02G403700.1	superfamily	381818	39	176	1.13E-55
Q#11 - >TraesCS2B02G459500.1	superfamily	381818	46	179	3.81E-39
Q#12 - >TraesCS2B02G459600.1	superfamily	381818	47	181	1.83E-40
Q#13 - >TraesCS2B02G459700.1	superfamily	381818	47	183	3.23E-53
Q#14 - >TraesCS2D02G382900.1	superfamily	381818	58	194	8.78E-57
Q#15 - >TraesCS2D02G436900.1	superfamily	381818	44	159	2.47E-33
Q#16 - >TraesCS2D02G437000.1	superfamily	381818	50	183	4.53E-40
Q#17 - >TraesCS2D02G437100.1	superfamily	381818	50	183	1.74E-40
Q#18 - >TraesCS2D02G437200.1	superfamily	381818	47	181	1.34E-39
Q#19 - >TraesCS2D02G437300.1	superfamily	381818	47	181	1.81E-40
Q#20 - >TraesCS2D02G437400.1	superfamily	381818	49	185	3.38E-55
Q#21 - >TraesCS3A02G477300.1	specific	270968	428	693	1.27E-87
Q#21 - >TraesCS3A02G477300.1	specific	349400	30	166	8.1E-77
Q#21 - >TraesCS3A02G477300.1	specific	349400	180	315	4.89E-73
Q#22 - >TraesCS3A02G525700.1	specific	349400	27	165	4.84E-63
Q#23 - >TraesCS3D02G472000.1	specific	270968	426	691	7.14E-89
Q#23 - >TraesCS3D02G472000.1	specific	349400	28	164	6.99E-79
Q#23 - >TraesCS3D02G472000.1	specific	349400	178	313	2.67E-71
Q#24 - >TraesCS3D02G530800.1	specific	349400	28	165	8.65E-63
Q#25 - >TraesCS4A02G251300.1	superfamily	381818	50	185	1.09E-56
Q#26 - >TraesCS4B02G063600.1	superfamily	381818	50	185	1.03E-55
Q#27 - >TraesCS4D02G062500.1	superfamily	381818	48	183	2E-56
Q#28 - >TraesCS5A02G012900.1	specific	349400	182	312	2.23E-74
Q#28 - >TraesCS5A02G012900.1	superfamily	237864	58	163	0.000139
Q#29 - >TraesCS5A02G059000.1	specific	349400	28	164	8.45E-75
Q#30 - >TraesCS5A02G183300.1	specific	349400	28	164	1.43E-79
Q#31 - >TraesCS5A02G439700.1	specific	349400	27	166	3.69E-85
Q#32 - >TraesCS5A02G439800.1	specific	349400	28	167	6.43E-84
Q#33 - >TraesCS5A02G439900.1	specific	349400	28	166	9.48E-80
Q#34 - >TraesCS5A02G440000.1	specific	349400	27	166	3.79E-67
Q#35 - >TraesCS5B02G011200.1	specific	349400	174	304	3.72E-72
Q#35 - >TraesCS5B02G011200.1	superfamily	236669	32	169	0.000426
Q#36 - >TraesCS5B02G066300.1	specific	349400	28	164	1.51E-72
Q#37 - >TraesCS5B02G181500.1	specific	349400	28	164	4.68E-87
Q#38 - >TraesCS5B02G442600.1	specific	349400	27	166	2.04E-86
Q#39 - >TraesCS5B02G442700.1	specific	349400	28	166	5.66E-84
Q#40 - >TraesCS5B02G442800.1	specific	349400	25	165	4.66E-78
Q#41 - >TraesCS5B02G442900.1	specific	349400	28	168	1.09E-77
Q#42 - >TraesCS5B02G443000.1	specific	349400	28	158	2.27E-69
Q#43 - >TraesCS5B02G443100.1	specific	349400	28	168	7.36E-77

Q#44 - >TraesCS5B02G443200.1	specific	349400	27	165	5.94E-84
Q#45 - >TraesCS5B02G443300.1	specific	349400	27	165	3.44E-82
Q#46 - >TraesCS5B02G443400.1	specific	349400	27	165	8.53E-84
Q#47 - >TraesCS5B02G443500.1	specific	349400	27	165	3.44E-82
Q#48 - >TraesCS5B02G443600.1	specific	349400	50	188	3.12E-84
Q#49 - >TraesCS5B02G443700.1	specific	349400	27	165	8.53E-84
Q#50 - >TraesCS5B02G443800.1	specific	349400	27	165	6.42E-84
Q#51 - >TraesCS5D02G446800.1	specific	349400	27	166	1.25E-85
Q#52 - >TraesCS5D02G446900.1	specific	349400	28	167	9.76E-84
Q#53 - >TraesCS5D02G447000.1	specific	349400	28	168	2.04E-78
Q#54 - >TraesCS5D02G447100.1	specific	349400	27	168	1.02E-63
Q#55 - >TraesCS6A02G345000.1	superfamily	381818	45	184	1.6E-48
Q#56 - >TraesCS6A02G345100.1	specific	349400	41	174	2.73E-78
Q#57 - >TraesCS6A02G345200.1	specific	349400	74	213	3.02E-62
Q#58 - >TraesCS6A02G346300.1	superfamily	381818	42	179	1.38E-36
Q#59 - >TraesCS6B02G377700.1	superfamily	381818	49	188	2.12E-48
Q#60 - >TraesCS6B02G377800.1	specific	349400	44	177	1.7E-78
Q#61 - >TraesCS6B02G378000.1	specific	349400	76	213	5.64E-61
Q#62 - >TraesCS6B02G379800.1	superfamily	381818	42	179	3.16E-36
Q#63 - >TraesCS6D02G327500.1	superfamily	381818	49	188	3.11E-47
Q#64 - >TraesCS6D02G327600.1	specific	349400	44	177	1.96E-78
Q#65 - >TraesCS6D02G327700.1	specific	349400	74	213	1.17E-62
Q#66 - >TraesCS6D02G329200.1	superfamily	381818	42	179	3.48E-35
Q#67 - >TraesCS7A02G152200.1	superfamily	381818	26	149	1.87E-26
Q#68 - >TraesCS7A02G198800.1	specific	349400	29	164	2.24E-89
Q#69 - >TraesCS7A02G198900.1	specific	349400	29	164	7.43E-90
Q#70 - >TraesCS7A02G565100.1	specific	349400	29	164	2.2E-83
Q#71 - >TraesCS7B02G056100.1	superfamily	381818	26	147	1.98E-28
Q#72 - >TraesCS7B02G104900.1	specific	349400	29	164	7.35E-88
Q#73 - >TraesCS7B02G105000.1	superfamily	381818	21	153	8.3E-08
Q#74 - >TraesCS7B02G105100.1	specific	349400	29	164	6.52E-89
Q#75 - >TraesCS7B02G105200.1	specific	349400	29	164	6.03E-89
Q#76 - >TraesCS7B02G105300.1	specific	349400	29	164	3.09E-89
Q#77 - >TraesCS7D02G099600.1	specific	349400	33	169	7.19E-81
Q#78 - >TraesCS7D02G153900.1	superfamily	381818	26	147	7.36E-25
Q#79 - >TraesCS7D02G161200.1	specific	349400	28	164	2.25E-78
Q#80 - >TraesCS7D02G201300.1	specific	349400	29	164	1.43E-89
Q#81 - >TraesCS7D02G201400.1	specific	349400	29	164	1.52E-90
Q#82 - >TraesCSU02G076600.1	specific	349400	28	168	1.5E-78
Q#83 - >TraesCSU02G095300.1	specific	349400	34	170	2.55E-82
Q#84 - >TraesCSU02G202900.1	specific	349400	28	168	1.5E-78
Q#85 - >TraesCSU02G226400.1	specific	349400	28	168	2.66E-78
Q#86 - >TraesCSU02G233000.1	specific	349400	28	168	1.5E-78

Query	Bitscore	Accession	Short name
Q#1 - >TraesCS1A02G443800.1	92.6968	cl00133	CAP_PR-1
Q#2 - >TraesCS1A02G444000.1	195.16	cd05381	CAP_PR-1
Q#3 - >TraesCS1B02G478300.1	98.0896	cl00133	CAP_PR-1
Q#4 - >TraesCS1B02G478500.1	194.775	cd05381	CAP_PR-1
Q#5 - >TraesCS1D02G452000.1	195.16	cd05381	CAP_PR-1
Q#6 - >TraesCS2A02G439600.1	115.424	cl00133	CAP_PR-1
Q#7 - >TraesCS2A02G439700.1	133.528	cl00133	CAP_PR-1
Q#8 - >TraesCS2A02G441400.1	158.566	cl00133	CAP_PR-1
Q#9 - >TraesCS2B02G403600.1	168.966	cl00133	CAP_PR-1
Q#10 - >TraesCS2B02G403700.1	171.663	cl00133	CAP_PR-1
Q#11 - >TraesCS2B02G459500.1	130.061	cl00133	CAP_PR-1
Q#12 - >TraesCS2B02G459600.1	133.528	cl00133	CAP_PR-1
Q#13 - >TraesCS2B02G459700.1	165.885	cl00133	CAP_PR-1
Q#14 - >TraesCS2D02G382900.1	175.515	cl00133	CAP_PR-1
Q#15 - >TraesCS2D02G436900.1	114.268	cl00133	CAP_PR-1
Q#16 - >TraesCS2D02G437000.1	132.372	cl00133	CAP_PR-1
Q#17 - >TraesCS2D02G437100.1	133.528	cl00133	CAP_PR-1
Q#18 - >TraesCS2D02G437200.1	131.217	cl00133	CAP_PR-1
Q#19 - >TraesCS2D02G437300.1	133.528	cl00133	CAP_PR-1
Q#20 - >TraesCS2D02G437400.1	170.892	cl00133	CAP_PR-1
Q#21 - >TraesCS3A02G477300.1	276.846	cd14066	STKc_IRAK
Q#21 - >TraesCS3A02G477300.1	243.31	cd05381	CAP_PR-1
Q#21 - >TraesCS3A02G477300.1	233.295	cd05381	CAP_PR-1
Q#22 - >TraesCS3A02G525700.1	190.152	cd05381	CAP_PR-1
Q#23 - >TraesCS3D02G472000.1	279.928	cd14066	STKc_IRAK
Q#23 - >TraesCS3D02G472000.1	248.703	cd05381	CAP_PR-1
Q#23 - >TraesCS3D02G472000.1	228.672	cd05381	CAP_PR-1
Q#24 - >TraesCS3D02G530800.1	189.382	cd05381	CAP_PR-1
Q#25 - >TraesCS4A02G251300.1	174.744	cl00133	CAP_PR-1
Q#26 - >TraesCS4B02G063600.1	172.433	cl00133	CAP_PR-1
Q#27 - >TraesCS4D02G062500.1	173.974	cl00133	CAP_PR-1
Q#28 - >TraesCS5A02G012900.1	224.435	cd05381	CAP_PR-1
Q#28 - >TraesCS5A02G012900.1	43.2596	cl36446	PRK14950
Q#29 - >TraesCS5A02G059000.1	219.813	cd05381	CAP_PR-1
Q#30 - >TraesCS5A02G183300.1	231.754	cd05381	CAP_PR-1
Q#31 - >TraesCS5A02G439700.1	246.006	cd05381	CAP_PR-1
Q#32 - >TraesCS5A02G439800.1	242.925	cd05381	CAP_PR-1
Q#33 - >TraesCS5A02G439900.1	232.524	cd05381	CAP_PR-1
Q#34 - >TraesCS5A02G440000.1	200.553	cd05381	CAP_PR-1
Q#35 - >TraesCS5B02G011200.1	218.657	cd05381	CAP_PR-1
Q#35 - >TraesCS5B02G011200.1	41.9943	cl35903	PRK10263
Q#36 - >TraesCS5B02G066300.1	214.035	cd05381	CAP_PR-1
Q#37 - >TraesCS5B02G181500.1	250.629	cd05381	CAP_PR-1
Q#38 - >TraesCS5B02G442600.1	249.088	cd05381	CAP_PR-1
Q#39 - >TraesCS5B02G442700.1	243.695	cd05381	CAP_PR-1
Q#40 - >TraesCS5B02G442800.1	227.902	cd05381	CAP_PR-1
Q#41 - >TraesCS5B02G442900.1	227.131	cd05381	CAP_PR-1
Q#42 - >TraesCS5B02G443000.1	205.946	cd05381	CAP_PR-1
Q#43 - >TraesCS5B02G443100.1	225.205	cd05381	CAP_PR-1

Q#44 - >TraesCS5B02G443200.1	242.925	cd05381	CAP_PR-1
Q#45 - >TraesCS5B02G443300.1	238.687	cd05381	CAP_PR-1
Q#46 - >TraesCS5B02G443400.1	242.539	cd05381	CAP_PR-1
Q#47 - >TraesCS5B02G443500.1	238.687	cd05381	CAP_PR-1
Q#48 - >TraesCS5B02G443600.1	244.465	cd05381	CAP_PR-1
Q#49 - >TraesCS5B02G443700.1	242.539	cd05381	CAP_PR-1
Q#50 - >TraesCS5B02G443800.1	242.925	cd05381	CAP_PR-1
Q#51 - >TraesCS5D02G446800.1	247.162	cd05381	CAP_PR-1
Q#52 - >TraesCS5D02G446900.1	242.539	cd05381	CAP_PR-1
Q#53 - >TraesCS5D02G447000.1	229.057	cd05381	CAP_PR-1
Q#54 - >TraesCS5D02G447100.1	192.078	cd05381	CAP_PR-1
Q#55 - >TraesCS6A02G345000.1	153.944	cl00133	CAP_PR-1
Q#56 - >TraesCS6A02G345100.1	229.057	cd05381	CAP_PR-1
Q#57 - >TraesCS6A02G345200.1	190.152	cd05381	CAP_PR-1
Q#58 - >TraesCS6A02G346300.1	123.513	cl00133	CAP_PR-1
Q#59 - >TraesCS6B02G377700.1	153.944	cl00133	CAP_PR-1
Q#60 - >TraesCS6B02G377800.1	229.828	cd05381	CAP_PR-1
Q#61 - >TraesCS6B02G378000.1	186.686	cd05381	CAP_PR-1
Q#62 - >TraesCS6B02G379800.1	122.357	cl00133	CAP_PR-1
Q#63 - >TraesCS6D02G327500.1	150.862	cl00133	CAP_PR-1
Q#64 - >TraesCS6D02G327600.1	229.443	cd05381	CAP_PR-1
Q#65 - >TraesCS6D02G327700.1	190.923	cd05381	CAP_PR-1
Q#66 - >TraesCS6D02G329200.1	120.046	cl00133	CAP_PR-1
Q#67 - >TraesCS7A02G152200.1	97.3192	cl00133	CAP_PR-1
Q#68 - >TraesCS7A02G198800.1	256.792	cd05381	CAP_PR-1
Q#69 - >TraesCS7A02G198900.1	258.333	cd05381	CAP_PR-1
Q#70 - >TraesCS7A02G565100.1	241.769	cd05381	CAP_PR-1
Q#71 - >TraesCS7B02G056100.1	102.327	cl00133	CAP_PR-1
Q#72 - >TraesCS7B02G104900.1	253.325	cd05381	CAP_PR-1
Q#73 - >TraesCS7B02G105000.1	48.3989	cl00133	CAP_PR-1
Q#74 - >TraesCS7B02G105100.1	256.021	cd05381	CAP_PR-1
Q#75 - >TraesCS7B02G105200.1	256.021	cd05381	CAP_PR-1
Q#76 - >TraesCS7B02G105300.1	256.792	cd05381	CAP_PR-1
Q#77 - >TraesCS7D02G099600.1	235.221	cd05381	CAP_PR-1
Q#78 - >TraesCS7D02G153900.1	93.082	cl00133	CAP_PR-1
Q#79 - >TraesCS7D02G161200.1	228.672	cd05381	CAP_PR-1
Q#80 - >TraesCS7D02G201300.1	257.562	cd05381	CAP_PR-1
Q#81 - >TraesCS7D02G201400.1	259.873	cd05381	CAP_PR-1
Q#82 - >TraesCSU02G076600.1	229.443	cd05381	CAP_PR-1
Q#83 - >TraesCSU02G095300.1	239.073	cd05381	CAP_PR-1
Q#84 - >TraesCSU02G202900.1	229.443	cd05381	CAP_PR-1
Q#85 - >TraesCSU02G226400.1	228.672	cd05381	CAP_PR-1
Q#86 - >TraesCSU02G233000.1	229.443	cd05381	CAP_PR-1
