# Supplementary material for: Characterization and functional analyses of wheat TaPR1 genes in response to stripe rust fungal infection
Source: Sci Rep. 2023 Feb 27;13:3362. doi: 10.1038/s41598-023-30456-8 (PMC9971213; doi:10.1038/s41598-023-30456-8)
Supplement: Supplementary file 4 — Supplementary Information 4. [file 41598_2023_30456_MOESM4_ESM.pdf]

Additional file 4. Cis-acting element prediction of the

Gene-ID	Start	End
TraesCS1A02G443800.1	214	260
TraesCS1A02G443800.1	239	288
TraesCS1A02G443800.1	263	312
TraesCS1A02G443800.1	267	313
TraesCS1A02G443800.1	464	510
TraesCS1A02G443800.1	618	666
TraesCS1A02G443800.1	881	927
TraesCS1A02G443800.1	1042	1090
TraesCS1A02G443800.1	1227	1277
TraesCS1A02G443800.1	1389	1434
TraesCS1A02G443800.1	1660	1708
TraesCS1A02G443800.1	1711	1757
TraesCS1A02G443800.1	1819	1864
TraesCS1A02G443800.1	1821	1866
TraesCS1A02G443800.1	1925	1971
TraesCS1A02G443800.1	1935	1980
TraesCS1A02G444000.1	32	78
TraesCS1A02G444000.1	113	159
TraesCS1A02G444000.1	507	552
TraesCS1A02G444000.1	565	610
TraesCS1A02G444000.1	644	689
TraesCS1A02G444000.1	826	875
TraesCS1A02G444000.1	854	901
TraesCS1A02G444000.1	1017	1064
TraesCS1A02G444000.1	1096	1142
TraesCS1A02G444000.1	1202	1248
TraesCS1A02G444000.1	1223	1268
TraesCS1A02G444000.1	1288	1335
TraesCS1A02G444000.1	1320	1366
TraesCS1A02G444000.1	1355	1401
TraesCS1A02G444000.1	1567	1612
TraesCS1A02G444000.1	1590	1638
TraesCS1A02G444000.1	1641	1687
TraesCS1A02G444000.1	1834	1881
TraesCS1A02G444000.1	1967	2015.5
TraesCS1B02G478300.1	10	57
TraesCS1B02G478300.1	111	158
TraesCS1B02G478300.1	204	253
TraesCS1B02G478300.1	425	471
TraesCS1B02G478300.1	541	589
TraesCS1B02G478300.1	670	716
TraesCS1B02G478300.1	674	720
TraesCS1B02G478300.1	804	851
TraesCS1B02G478300.1	805	851
TraesCS1B02G478300.1	854	900
TraesCS1B02G478300.1	903	948
TraesCS1B02G478300.1	980	1028
TraesCS1B02G478300.1	1045	1090
TraesCS1B02G478300.1	1073	1120
TraesCS1B02G478300.1	1198	1248
TraesCS1B02G478300.1	1412	1459
TraesCS1B02G478300.1	1441	1488
TraesCS1B02G478300.1	1700	1746
TraesCS1B02G478300.1	1812	1857
TraesCS1B02G478300.1	1814	1859
TraesCS1B02G478300.1	1914	1960

TraesCS1B02G478300.1	1924	1969
TraesCS1B02G478500.1	19	66
TraesCS1B02G478500.1	22	68
TraesCS1B02G478500.1	82	128
TraesCS1B02G478500.1	166	212
TraesCS1B02G478500.1	167	212
TraesCS1B02G478500.1	184	230
TraesCS1B02G478500.1	271	317
TraesCS1B02G478500.1	285	331
TraesCS1B02G478500.1	302	351
TraesCS1B02G478500.1	303	348
TraesCS1B02G478500.1	305	350
TraesCS1B02G478500.1	311	357
TraesCS1B02G478500.1	962	1008
TraesCS1B02G478500.1	1001	1047
TraesCS1B02G478500.1	1148	1197
TraesCS1B02G478500.1	1285	1331
TraesCS1B02G478500.1	1289	1335
TraesCS1B02G478500.1	1316	1362
TraesCS1B02G478500.1	1326	1375
TraesCS1B02G478500.1	1326	1375
TraesCS1B02G478500.1	1477	1522
TraesCS1B02G478500.1	1633	1679
TraesCS1B02G478500.1	1633	1679
TraesCS1B02G478500.1	1634	1679
TraesCS1B02G478500.1	1839	1886
TraesCS1D02G452000.1	53	98
TraesCS1D02G452000.1	97	145
TraesCS1D02G452000.1	504	550
TraesCS1D02G452000.1	642	691
TraesCS1D02G452000.1	851	897
TraesCS1D02G452000.1	1306	1352
TraesCS1D02G452000.1	1335	1381
TraesCS1D02G452000.1	1363	1409
TraesCS1D02G452000.1	1375	1421
TraesCS1D02G452000.1	1375	1421
TraesCS1D02G452000.1	1376	1421
TraesCS1D02G452000.1	1388	1434
TraesCS1D02G452000.1	1408	1456
TraesCS1D02G452000.1	1509	1555
TraesCS1D02G452000.1	1511	1558
TraesCS1D02G452000.1	1558	1605
TraesCS1D02G452000.1	1718	1767
TraesCS1D02G452000.1	1856	1903
TraesCS1D02G452000.1	1880	1927
TraesCS2A02G439600.1	117	163
TraesCS2A02G439600.1	259	308
TraesCS2A02G439600.1	260	305
TraesCS2A02G439600.1	655	705
TraesCS2A02G439600.1	782	828
TraesCS2A02G439600.1	858	903
TraesCS2A02G439600.1	885	933
TraesCS2A02G439600.1	1067	1113
TraesCS2A02G439600.1	1161	1210
TraesCS2A02G439600.1	1235	1281
TraesCS2A02G439600.1	1296	1342
TraesCS2A02G439600.1	1563	1609
TraesCS2A02G439600.1	1564	1609

TraesCS2A02G439600.1	1642	1688
TraesCS2A02G439600.1	1685	1734
TraesCS2A02G439600.1	1731	1777
TraesCS2A02G439600.1	1803	1849
TraesCS2A02G439600.1	1856	1901
TraesCS2A02G439600.1	1914	1963
TraesCS2A02G439600.1	1915	1960
TraesCS2A02G439600.1	1917	1962
TraesCS2A02G439600.1	1945	1991
TraesCS2A02G439600.1	1969	2016
TraesCS2A02G439700.1	450	499
TraesCS2A02G439700.1	488	537
TraesCS2A02G439700.1	498	544
TraesCS2A02G439700.1	532	578
TraesCS2A02G439700.1	642	692
TraesCS2A02G439700.1	670	718
TraesCS2A02G439700.1	714	759
TraesCS2A02G439700.1	999	1044
TraesCS2A02G439700.1	1005	1055
TraesCS2A02G439700.1	1337	1382
TraesCS2A02G439700.1	1337	1383
TraesCS2A02G439700.1	1475	1521
TraesCS2A02G439700.1	1573	1620
TraesCS2A02G439700.1	1594	1641
TraesCS2A02G439700.1	1667	1713
TraesCS2A02G439700.1	1717	1762
TraesCS2A02G439700.1	1738	1784
TraesCS2A02G439700.1	1769	1816
TraesCS2A02G439700.1	1862	1907
TraesCS2A02G439700.1	1929	1978
TraesCS2A02G439700.1	1930	1975
TraesCS2A02G439700.1	1930	1976
TraesCS2A02G439700.1	1932	1977
TraesCS2A02G441400.1	126	175
TraesCS2A02G441400.1	184	231
TraesCS2A02G441400.1	213	262
TraesCS2A02G441400.1	312	361
TraesCS2A02G441400.1	528	574
TraesCS2A02G441400.1	663	709
TraesCS2A02G441400.1	787	833
TraesCS2A02G441400.1	797	843
TraesCS2A02G441400.1	905	951
TraesCS2A02G441400.1	1091	1138
TraesCS2A02G441400.1	1158	1207
TraesCS2A02G441400.1	1198	1247
TraesCS2A02G441400.1	1228	1273
TraesCS2A02G441400.1	1241	1287
TraesCS2A02G441400.1	1445	1490
TraesCS2A02G441400.1	1575	1620
TraesCS2A02G441400.1	1667	1713
TraesCS2A02G441400.1	1831	1877
TraesCS2A02G441400.1	1881	1926
TraesCS2A02G441400.1	1902	1948
TraesCS2A02G441400.1	1909	1955
TraesCS2A02G441400.1	1932	1977
TraesCS2A02G441400.1	1960	2006
TraesCS2B02G403600.1	33	79
TraesCS2B02G403600.1	100	146

TraesCS2B02G403600.1	162	208
TraesCS2B02G403600.1	219	266
TraesCS2B02G403600.1	251	296
TraesCS2B02G403600.1	457	503
TraesCS2B02G403600.1	726	771
TraesCS2B02G403600.1	775	824
TraesCS2B02G403600.1	777	826
TraesCS2B02G403600.1	822	867
TraesCS2B02G403600.1	1250	1296
TraesCS2B02G403600.1	1251	1296
TraesCS2B02G403600.1	1325	1371
TraesCS2B02G403600.1	1496	1545
TraesCS2B02G403600.1	1498	1543
TraesCS2B02G403600.1	1499	1545
TraesCS2B02G403600.1	1500	1545
TraesCS2B02G403600.1	1568	1614
TraesCS2B02G403600.1	1610	1660
TraesCS2B02G403600.1	1649	1694
TraesCS2B02G403600.1	1771	1820
TraesCS2B02G403600.1	1908	1955
TraesCS2B02G403700.1	316	362
TraesCS2B02G403700.1	317	362
TraesCS2B02G403700.1	391	437
TraesCS2B02G403700.1	562	611
TraesCS2B02G403700.1	564	609
TraesCS2B02G403700.1	565	611
TraesCS2B02G403700.1	566	611
TraesCS2B02G403700.1	634	680
TraesCS2B02G403700.1	676	726
TraesCS2B02G403700.1	715	760
TraesCS2B02G403700.1	837	886
TraesCS2B02G403700.1	974	1021
TraesCS2B02G403700.1	1061	1110
TraesCS2B02G403700.1	1130	1175
TraesCS2B02G403700.1	1142	1187
TraesCS2B02G403700.1	1261	1306
TraesCS2B02G403700.1	1262	1308
TraesCS2B02G403700.1	1263	1308
TraesCS2B02G403700.1	1428	1474
TraesCS2B02G403700.1	1503	1549
TraesCS2B02G403700.1	1517	1563
TraesCS2B02G403700.1	1537	1583
TraesCS2B02G403700.1	1558	1608
TraesCS2B02G403700.1	1560	1609
TraesCS2B02G403700.1	1587	1633
TraesCS2B02G403700.1	1630	1676
TraesCS2B02G403700.1	1672	1722
TraesCS2B02G403700.1	1673	1722
TraesCS2B02G403700.1	1674	1720
TraesCS2B02G403700.1	1675	1720
TraesCS2B02G403700.1	1904	1950
TraesCS2B02G403700.1	1908	1955
TraesCS2B02G403700.1	1909	1955
TraesCS2B02G459500.1	-13	32
TraesCS2B02G459500.1	9	55
TraesCS2B02G459500.1	56	103
TraesCS2B02G459500.1	81	131
TraesCS2B02G459500.1	85	132

TraesCS2B02G459500.1	102	149
TraesCS2B02G459500.1	295	341
TraesCS2B02G459500.1	350	400
TraesCS2B02G459500.1	568	614
TraesCS2B02G459500.1	610	656
TraesCS2B02G459500.1	715	765
TraesCS2B02G459500.1	844	890
TraesCS2B02G459500.1	947	995
TraesCS2B02G459500.1	970	1017
TraesCS2B02G459500.1	1128	1174
TraesCS2B02G459500.1	1223	1272
TraesCS2B02G459500.1	1357	1403
TraesCS2B02G459500.1	1619	1664
TraesCS2B02G459500.1	1770	1816
TraesCS2B02G459500.1	1816	1865
TraesCS2B02G459500.1	1818	1864
TraesCS2B02G459500.1	1819	1864
TraesCS2B02G459500.1	1872	1917
TraesCS2B02G459500.1	1893	1939
TraesCS2B02G459500.1	1920	1965
TraesCS2B02G459500.1	1922	1967
TraesCS2B02G459500.1	1951	1997
TraesCS2B02G459600.1	-6	41
TraesCS2B02G459600.1	39	85
TraesCS2B02G459600.1	185	232
TraesCS2B02G459600.1	263	310
TraesCS2B02G459600.1	278	328
TraesCS2B02G459600.1	399	449
TraesCS2B02G459600.1	696	745
TraesCS2B02G459600.1	772	822
TraesCS2B02G459600.1	823	869
TraesCS2B02G459600.1	953	998
TraesCS2B02G459600.1	1063	1110
TraesCS2B02G459600.1	1218	1265
TraesCS2B02G459600.1	1438	1484
TraesCS2B02G459600.1	1573	1618
TraesCS2B02G459600.1	1573	1619
TraesCS2B02G459600.1	1744	1791
TraesCS2B02G459600.1	1779	1828
TraesCS2B02G459600.1	1783	1832
TraesCS2B02G459600.1	1832	1878
TraesCS2B02G459600.1	1882	1927
TraesCS2B02G459600.1	1903	1949
TraesCS2B02G459600.1	1930	1975
TraesCS2B02G459600.1	1932	1977
TraesCS2B02G459600.1	1960	2006
TraesCS2B02G459700.1	1000	1045
TraesCS2B02G459700.1	1930	1975
TraesCS2B02G459700.1	1485	1532
TraesCS2B02G459700.1	1750	1796
TraesCS2B02G459700.1	1960	2006
TraesCS2B02G459700.1	1152	1199
TraesCS2B02G459700.1	999	1045
TraesCS2B02G459700.1	1554	1600
TraesCS2B02G459700.1	1930	1976
TraesCS2B02G459700.1	397	443
TraesCS2B02G459700.1	504	550
TraesCS2B02G459700.1	409	456

TraesCS2B02G459700.1	1111	1158
TraesCS2B02G459700.1	1112	1158
TraesCS2B02G459700.1	1903	1949
TraesCS2B02G459700.1	1861	1906
TraesCS2B02G459700.1	1932	1977
TraesCS2B02G459700.1	1861	1906
TraesCS2B02G459700.1	1932	1977
TraesCS2B02G459700.1	605	651
TraesCS2B02G459700.1	1631	1677
TraesCS2B02G459700.1	251	300
TraesCS2D02G382900.1	16	62
TraesCS2D02G382900.1	17	62
TraesCS2D02G382900.1	178	223
TraesCS2D02G382900.1	277	322
TraesCS2D02G382900.1	284	329
TraesCS2D02G382900.1	602	647
TraesCS2D02G382900.1	766	811
TraesCS2D02G382900.1	1304	1349
TraesCS2D02G382900.1	1601	1650
TraesCS2D02G382900.1	1715	1761
TraesCS2D02G382900.1	1716	1761
TraesCS2D02G382900.1	1024	1071
TraesCS2D02G382900.1	1069	1115
TraesCS2D02G382900.1	16	62
TraesCS2D02G382900.1	178	224
TraesCS2D02G382900.1	1715	1761
TraesCS2D02G382900.1	16	62
TraesCS2D02G382900.1	276	322
TraesCS2D02G382900.1	284	330
TraesCS2D02G382900.1	601	647
TraesCS2D02G382900.1	766	812
TraesCS2D02G382900.1	1303	1349
TraesCS2D02G382900.1	1544	1590
TraesCS2D02G382900.1	1558	1604
TraesCS2D02G382900.1	1713	1762
TraesCS2D02G382900.1	1715	1761
TraesCS2D02G382900.1	1959	2005
TraesCS2D02G382900.1	719	769
TraesCS2D02G382900.1	336	383
TraesCS2D02G382900.1	1599	1649
TraesCS2D02G382900.1	1963	2010
TraesCS2D02G382900.1	1964	2010
TraesCS2D02G382900.1	677	723
TraesCS2D02G382900.1	1469	1515
TraesCS2D02G382900.1	600	645
TraesCS2D02G382900.1	1171	1216
TraesCS2D02G382900.1	1183	1228
TraesCS2D02G382900.1	1302	1347
TraesCS2D02G382900.1	600	645
TraesCS2D02G382900.1	1171	1216
TraesCS2D02G382900.1	1183	1228
TraesCS2D02G382900.1	1302	1347
TraesCS2D02G382900.1	229	275
TraesCS2D02G382900.1	1671	1717
TraesCS2D02G382900.1	598	647
TraesCS2D02G436900.1	1305	1350
TraesCS2D02G436900.1	1420	1465
TraesCS2D02G436900.1	1551	1596

TraesCS2D02G436900.1	1915	1960
TraesCS2D02G436900.1	1718	1764
TraesCS2D02G436900.1	1945	1991
TraesCS2D02G436900.1	1969	2016
TraesCS2D02G436900.1	1764	1813
TraesCS2D02G436900.1	1304	1350
TraesCS2D02G436900.1	1238	1284
TraesCS2D02G436900.1	1419	1465
TraesCS2D02G436900.1	1550	1596
TraesCS2D02G436900.1	1915	1961
TraesCS2D02G436900.1	1771	1817
TraesCS2D02G436900.1	1793	1839
TraesCS2D02G436900.1	954	1003
TraesCS2D02G436900.1	963	1010
TraesCS2D02G436900.1	1254	1301
TraesCS2D02G436900.1	1672	1721
TraesCS2D02G436900.1	1888	1934
TraesCS2D02G436900.1	191	236
TraesCS2D02G436900.1	645	690
TraesCS2D02G436900.1	718	763
TraesCS2D02G436900.1	1226	1271
TraesCS2D02G436900.1	1245	1290
TraesCS2D02G436900.1	1291	1336
TraesCS2D02G436900.1	1843	1888
TraesCS2D02G436900.1	1917	1962
TraesCS2D02G436900.1	191	236
TraesCS2D02G436900.1	645	690
TraesCS2D02G436900.1	718	763
TraesCS2D02G436900.1	1226	1271
TraesCS2D02G436900.1	1245	1290
TraesCS2D02G436900.1	1291	1336
TraesCS2D02G436900.1	1843	1888
TraesCS2D02G436900.1	1917	1962
TraesCS2D02G437000.1	-11	38
TraesCS2D02G437000.1	69	114
TraesCS2D02G437000.1	1534	1579
TraesCS2D02G437000.1	1930	1975
TraesCS2D02G437000.1	1960	2006
TraesCS2D02G437000.1	1730	1777
TraesCS2D02G437000.1	68	114
TraesCS2D02G437000.1	181	227
TraesCS2D02G437000.1	1534	1580
TraesCS2D02G437000.1	1930	1976
TraesCS2D02G437000.1	1814	1860
TraesCS2D02G437000.1	1533	1582
TraesCS2D02G437000.1	1747	1794
TraesCS2D02G437000.1	1639	1685
TraesCS2D02G437000.1	399	445
TraesCS2D02G437000.1	479	525
TraesCS2D02G437000.1	1291	1337
TraesCS2D02G437000.1	1440	1486
TraesCS2D02G437000.1	1488	1534
TraesCS2D02G437000.1	1903	1949
TraesCS2D02G437000.1	637	682
TraesCS2D02G437000.1	1867	1912
TraesCS2D02G437000.1	1932	1977
TraesCS2D02G437000.1	637	682
TraesCS2D02G437000.1	1867	1912

TraesCS2D02G437000.1	1932	1977
TraesCS2D02G437000.1	1342	1391
TraesCS2D02G437100.1	120	169
TraesCS2D02G437100.1	1465	1510
TraesCS2D02G437100.1	1930	1975
TraesCS2D02G437100.1	1960	2006
TraesCS2D02G437100.1	426	473
TraesCS2D02G437100.1	492	538
TraesCS2D02G437100.1	1465	1511
TraesCS2D02G437100.1	1930	1976
TraesCS2D02G437100.1	828	874
TraesCS2D02G437100.1	1835	1881
TraesCS2D02G437100.1	1655	1702
TraesCS2D02G437100.1	678	725
TraesCS2D02G437100.1	1720	1767
TraesCS2D02G437100.1	1	47
TraesCS2D02G437100.1	1902	1949
TraesCS2D02G437100.1	1903	1949
TraesCS2D02G437100.1	815	860
TraesCS2D02G437100.1	1561	1606
TraesCS2D02G437100.1	1932	1977
TraesCS2D02G437100.1	815	860
TraesCS2D02G437100.1	1561	1606
TraesCS2D02G437100.1	1932	1977
TraesCS2D02G437100.1	384	430
TraesCS2D02G437100.1	745	794
TraesCS2D02G437100.1	785	834
TraesCS2D02G437200.1	859	904
TraesCS2D02G437200.1	1102	1147
TraesCS2D02G437200.1	1484	1529
TraesCS2D02G437200.1	461	508
TraesCS2D02G437200.1	1960	2006
TraesCS2D02G437200.1	1122	1169
TraesCS2D02G437200.1	1737	1784
TraesCS2D02G437200.1	497	544
TraesCS2D02G437200.1	858	904
TraesCS2D02G437200.1	1101	1147
TraesCS2D02G437200.1	1484	1530
TraesCS2D02G437200.1	345	395
TraesCS2D02G437200.1	270	319
TraesCS2D02G437200.1	1619	1665
TraesCS2D02G437200.1	968	1015
TraesCS2D02G437200.1	1758	1805
TraesCS2D02G437200.1	395	442
TraesCS2D02G437200.1	396	442
TraesCS2D02G437200.1	1903	1949
TraesCS2D02G437200.1	1882	1927
TraesCS2D02G437200.1	1932	1977
TraesCS2D02G437200.1	1882	1927
TraesCS2D02G437200.1	1932	1977
TraesCS2D02G437200.1	1421	1470
TraesCS2D02G437300.1	86	135
TraesCS2D02G437300.1	338	384
TraesCS2D02G437300.1	339	384
TraesCS2D02G437300.1	1504	1549
TraesCS2D02G437300.1	1930	1975
TraesCS2D02G437300.1	1193	1239
TraesCS2D02G437300.1	1960	2006

TraesCS2D02G437300.1	36	85
TraesCS2D02G437300.1	338	384
TraesCS2D02G437300.1	456	502
TraesCS2D02G437300.1	1504	1550
TraesCS2D02G437300.1	1930	1976
TraesCS2D02G437300.1	338	384
TraesCS2D02G437300.1	791	837
TraesCS2D02G437300.1	1832	1878
TraesCS2D02G437300.1	1693	1740
TraesCS2D02G437300.1	61	107
TraesCS2D02G437300.1	134	180
TraesCS2D02G437300.1	198	244
TraesCS2D02G437300.1	1903	1949
TraesCS2D02G437300.1	778	823
TraesCS2D02G437300.1	1121	1166
TraesCS2D02G437300.1	1882	1927
TraesCS2D02G437300.1	1932	1977
TraesCS2D02G437300.1	778	823
TraesCS2D02G437300.1	1121	1166
TraesCS2D02G437300.1	1882	1927
TraesCS2D02G437300.1	1932	1977
TraesCS2D02G437300.1	348	394
TraesCS2D02G437300.1	1213	1259
TraesCS2D02G437300.1	1077	1126
TraesCS2D02G437300.1	708	757
TraesCS2D02G437300.1	748	797
TraesCS2D02G437400.1	166	211
TraesCS2D02G437400.1	327	376
TraesCS2D02G437400.1	330	375
TraesCS2D02G437400.1	1238	1284
TraesCS2D02G437400.1	1239	1284
TraesCS2D02G437400.1	1929	1974
TraesCS2D02G437400.1	1364	1413
TraesCS2D02G437400.1	991	1038
TraesCS2D02G437400.1	1651	1698
TraesCS2D02G437400.1	329	375
TraesCS2D02G437400.1	1238	1284
TraesCS2D02G437400.1	165	211
TraesCS2D02G437400.1	1236	1285
TraesCS2D02G437400.1	1238	1284
TraesCS2D02G437400.1	1929	1975
TraesCS2D02G437400.1	1434	1481
TraesCS2D02G437400.1	1642	1689
TraesCS2D02G437400.1	1330	1376
TraesCS2D02G437400.1	1484	1530
TraesCS2D02G437400.1	1833	1879
TraesCS2D02G437400.1	1266	1312
TraesCS2D02G437400.1	1586	1632
TraesCS2D02G437400.1	186	234
TraesCS2D02G437400.1	1904	1950
TraesCS2D02G437400.1	1214	1260
TraesCS2D02G437400.1	1883	1928
TraesCS2D02G437400.1	1931	1976
TraesCS2D02G437400.1	1883	1928
TraesCS2D02G437400.1	1931	1976
TraesCS3A02G477300.1	24	69
TraesCS3A02G477300.1	82	127
TraesCS3A02G477300.1	94	140

TraesCS3A02G477300.1	140	189
TraesCS3A02G477300.1	154	199
TraesCS3A02G477300.1	167	213
TraesCS3A02G477300.1	168	213
TraesCS3A02G477300.1	599	645
TraesCS3A02G477300.1	700	746
TraesCS3A02G477300.1	1235	1284
TraesCS3A02G477300.1	1327	1373
TraesCS3A02G477300.1	1384	1429
TraesCS3A02G477300.1	1488	1533
TraesCS3A02G477300.1	1496	1545
TraesCS3A02G477300.1	1552	1601
TraesCS3A02G477300.1	1570	1615
TraesCS3A02G477300.1	1606	1651
TraesCS3A02G477300.1	1713	1758
TraesCS3A02G477300.1	1865	1910
TraesCS3A02G477300.1	1876	1921
TraesCS3A02G525700.1	127	172
TraesCS3A02G525700.1	1361	1407
TraesCS3A02G525700.1	126	172
TraesCS3A02G525700.1	398	444
TraesCS3A02G525700.1	1494	1540
TraesCS3A02G525700.1	546	595
TraesCS3A02G525700.1	890	939
TraesCS3A02G525700.1	1075	1124
TraesCS3A02G525700.1	1336	1385
TraesCS3A02G525700.1	1920	1969
TraesCS3A02G525700.1	323	370
TraesCS3A02G525700.1	984	1030
TraesCS3A02G525700.1	1684	1733
TraesCS3A02G525700.1	573	623
TraesCS3A02G525700.1	687	734
TraesCS3A02G525700.1	1633	1679
TraesCS3A02G525700.1	656	701
TraesCS3A02G525700.1	873	918
TraesCS3A02G525700.1	876	921
TraesCS3A02G525700.1	1120	1165
TraesCS3A02G525700.1	1448	1493
TraesCS3A02G525700.1	656	701
TraesCS3A02G525700.1	873	918
TraesCS3A02G525700.1	876	921
TraesCS3A02G525700.1	1120	1165
TraesCS3A02G525700.1	1448	1493
TraesCS3D02G472000.1	220	266
TraesCS3D02G472000.1	390	436
TraesCS3D02G472000.1	1190	1236
TraesCS3D02G472000.1	1285	1331
TraesCS3D02G472000.1	1352	1398
TraesCS3D02G472000.1	1365	1410
TraesCS3D02G472000.1	1376	1422
TraesCS3D02G472000.1	1531	1577
TraesCS3D02G472000.1	1703	1752
TraesCS3D02G472000.1	1706	1751
TraesCS3D02G472000.1	1864	1909
TraesCS3D02G472000.1	1939	1988
TraesCS3D02G530800.1	209	258
TraesCS3D02G530800.1	753	799
TraesCS3D02G530800.1	1343	1389

TraesCS3D02G530800.1	201	247
TraesCS3D02G530800.1	1066	1115
TraesCS3D02G530800.1	1318	1367
TraesCS3D02G530800.1	1920	1969
TraesCS3D02G530800.1	625	671
TraesCS3D02G530800.1	1215	1261
TraesCS3D02G530800.1	1474	1520
TraesCS3D02G530800.1	946	996
TraesCS3D02G530800.1	1844	1893
TraesCS3D02G530800.1	1111	1156
TraesCS3D02G530800.1	1428	1473
TraesCS3D02G530800.1	1111	1156
TraesCS3D02G530800.1	1428	1473
TraesCS3D02G530800.1	210	256
TraesCS4A02G251300.1	1423	1468
TraesCS4A02G251300.1	1684	1733
TraesCS4A02G251300.1	1815	1864
TraesCS4A02G251300.1	1157	1204
TraesCS4A02G251300.1	1422	1468
TraesCS4A02G251300.1	968	1018
TraesCS4A02G251300.1	1690	1736
TraesCS4A02G251300.1	211	256
TraesCS4A02G251300.1	1624	1669
TraesCS4A02G251300.1	211	256
TraesCS4A02G251300.1	1624	1669
TraesCS4A02G251300.1	199	248
TraesCS4A02G251300.1	931	979.5
TraesCS4B02G063600.1	791	836
TraesCS4B02G063600.1	970	1015
TraesCS4B02G063600.1	1443	1488
TraesCS4B02G063600.1	1591	1636
TraesCS4B02G063600.1	1815	1864
TraesCS4B02G063600.1	99	145
TraesCS4B02G063600.1	127	173
TraesCS4B02G063600.1	88	134
TraesCS4B02G063600.1	791	837
TraesCS4B02G063600.1	969	1015
TraesCS4B02G063600.1	1442	1488
TraesCS4B02G063600.1	1590	1636
TraesCS4B02G063600.1	1371	1417
TraesCS4B02G063600.1	693	739
TraesCS4B02G063600.1	1132	1178
TraesCS4B02G063600.1	45	90
TraesCS4B02G063600.1	671	716
TraesCS4B02G063600.1	1335	1380
TraesCS4B02G063600.1	1594	1639
TraesCS4B02G063600.1	1652	1697
TraesCS4B02G063600.1	1664	1709
TraesCS4B02G063600.1	45	90
TraesCS4B02G063600.1	671	716
TraesCS4B02G063600.1	1335	1380
TraesCS4B02G063600.1	1594	1639
TraesCS4B02G063600.1	1652	1697
TraesCS4B02G063600.1	1664	1709
TraesCS4D02G062500.1	91	140
TraesCS4D02G062500.1	692	739
TraesCS4D02G062500.1	1271	1316
TraesCS4D02G062500.1	1437	1482

TraesCS4D02G062500.1	1820	1869
TraesCS4D02G062500.1	1030	1077
TraesCS4D02G062500.1	1271	1317
TraesCS4D02G062500.1	1436	1482
TraesCS4D02G062500.1	894	940
TraesCS4D02G062500.1	335	382
TraesCS4D02G062500.1	1604	1650
TraesCS4D02G062500.1	1106	1152
TraesCS4D02G062500.1	903	948
TraesCS4D02G062500.1	1632	1677
TraesCS4D02G062500.1	903	948
TraesCS4D02G062500.1	1632	1677
TraesCS4D02G062500.1	1069	1115
TraesCS5A02G012900.1	786	833
TraesCS5A02G012900.1	931	977
TraesCS5A02G012900.1	1052	1097
TraesCS5A02G012900.1	1064	1110
TraesCS5A02G012900.1	1150	1195
TraesCS5A02G012900.1	1152	1197
TraesCS5A02G012900.1	1181	1226
TraesCS5A02G012900.1	1183	1228
TraesCS5A02G012900.1	1218	1265
TraesCS5A02G012900.1	1619	1670
TraesCS5A02G012900.1	1911	1956
TraesCS5A02G012900.1	1954	2004
TraesCS5A02G012900.1	1971	2017
TraesCS5A02G059000.1	716	761
TraesCS5A02G059000.1	1734	1783
TraesCS5A02G059000.1	366	415
TraesCS5A02G059000.1	716	762
TraesCS5A02G059000.1	1639	1685
TraesCS5A02G059000.1	1731	1777
TraesCS5A02G059000.1	1403	1450
TraesCS5A02G059000.1	1870	1919
TraesCS5A02G059000.1	1452	1498
TraesCS5A02G059000.1	954	999
TraesCS5A02G059000.1	954	999
TraesCS5A02G059000.1	323	371.5
TraesCS5A02G059000.1	1120	1169
TraesCS5A02G183300.1	1611	1656
TraesCS5A02G183300.1	1774	1823
TraesCS5A02G183300.1	812	858
TraesCS5A02G183300.1	868	914
TraesCS5A02G183300.1	1302	1348
TraesCS5A02G183300.1	1499	1545
TraesCS5A02G183300.1	179	226
TraesCS5A02G183300.1	1610	1656
TraesCS5A02G183300.1	518	564
TraesCS5A02G183300.1	986	1036
TraesCS5A02G183300.1	1648	1695
TraesCS5A02G183300.1	88	136
TraesCS5A02G183300.1	1662	1709
TraesCS5A02G183300.1	1663	1709
TraesCS5A02G183300.1	-1	44
TraesCS5A02G183300.1	2	47
TraesCS5A02G183300.1	1458	1503
TraesCS5A02G183300.1	1461	1506
TraesCS5A02G183300.1	-1	44

TraesCS5A02G183300.1	2	47
TraesCS5A02G183300.1	1458	1503
TraesCS5A02G183300.1	1461	1506
TraesCS5A02G183300.1	120	169
TraesCS5A02G183300.1	594	643
TraesCS5A02G183300.1	1458	1507
TraesCS5A02G439700.1	1500	1545
TraesCS5A02G439700.1	865	912
TraesCS5A02G439700.1	888	936
TraesCS5A02G439700.1	541	587
TraesCS5A02G439700.1	834	880
TraesCS5A02G439700.1	896	942
TraesCS5A02G439700.1	1500	1546
TraesCS5A02G439700.1	1002	1049
TraesCS5A02G439700.1	1376	1424
TraesCS5A02G439700.1	789	839
TraesCS5A02G439700.1	1877	1924
TraesCS5A02G439700.1	1222	1268
TraesCS5A02G439700.1	796	841
TraesCS5A02G439700.1	891	936
TraesCS5A02G439700.1	1148	1193
TraesCS5A02G439700.1	1430	1475
TraesCS5A02G439700.1	1558	1603
TraesCS5A02G439700.1	796	841
TraesCS5A02G439700.1	891	936
TraesCS5A02G439700.1	1148	1193
TraesCS5A02G439700.1	1430	1475
TraesCS5A02G439700.1	1558	1603
TraesCS5A02G439700.1	77	123
TraesCS5A02G439700.1	1939	1988
TraesCS5A02G439700.1	1415	1464
TraesCS5A02G439800.1	258	305
TraesCS5A02G439800.1	1464	1510
TraesCS5A02G439800.1	92	138
TraesCS5A02G439800.1	1333	1379
TraesCS5A02G439800.1	372	421
TraesCS5A02G439800.1	108	155
TraesCS5A02G439800.1	1554	1601
TraesCS5A02G439800.1	1630	1676
TraesCS5A02G439800.1	591	639
TraesCS5A02G439800.1	380	426
TraesCS5A02G439800.1	80	125
TraesCS5A02G439800.1	99	144
TraesCS5A02G439800.1	146	191
TraesCS5A02G439800.1	393	438
TraesCS5A02G439800.1	617	662
TraesCS5A02G439800.1	1072	1117
TraesCS5A02G439800.1	1189	1234
TraesCS5A02G439800.1	80	125
TraesCS5A02G439800.1	99	144
TraesCS5A02G439800.1	146	191
TraesCS5A02G439800.1	393	438
TraesCS5A02G439800.1	617	662
TraesCS5A02G439800.1	1072	1117
TraesCS5A02G439800.1	1189	1234
TraesCS5A02G439800.1	68	117
TraesCS5A02G439800.1	829	878
TraesCS5A02G439900.1	112	157

TraesCS5A02G439900.1	138	183
TraesCS5A02G439900.1	212	257
TraesCS5A02G439900.1	1036	1081
TraesCS5A02G439900.1	1090	1135
TraesCS5A02G439900.1	1480	1526
TraesCS5A02G439900.1	1481	1526
TraesCS5A02G439900.1	1565	1610
TraesCS5A02G439900.1	1606	1651
TraesCS5A02G439900.1	103	149
TraesCS5A02G439900.1	1450	1496
TraesCS5A02G439900.1	612	658
TraesCS5A02G439900.1	687	734
TraesCS5A02G439900.1	112	158
TraesCS5A02G439900.1	137	183
TraesCS5A02G439900.1	212	258
TraesCS5A02G439900.1	1480	1526
TraesCS5A02G439900.1	1605	1651
TraesCS5A02G439900.1	1036	1082
TraesCS5A02G439900.1	1090	1136
TraesCS5A02G439900.1	1480	1526
TraesCS5A02G439900.1	1565	1611
TraesCS5A02G439900.1	245	293
TraesCS5A02G439900.1	291	339
TraesCS5A02G439900.1	321	369
TraesCS5A02G439900.1	765	817
TraesCS5A02G439900.1	1097	1147
TraesCS5A02G439900.1	1751	1797
TraesCS5A02G439900.1	1125	1171
TraesCS5A02G439900.1	91	136
TraesCS5A02G439900.1	114	159
TraesCS5A02G439900.1	1697	1742
TraesCS5A02G439900.1	91	136
TraesCS5A02G439900.1	114	159
TraesCS5A02G439900.1	1697	1742
TraesCS5A02G439900.1	1405	1451
TraesCS5A02G439900.1	1280	1329
TraesCS5A02G439900.1	179	228
TraesCS5A02G440000.1	1021	1066
TraesCS5A02G440000.1	1074	1119
TraesCS5A02G440000.1	1226	1271
TraesCS5A02G440000.1	278	325
TraesCS5A02G440000.1	16	62
TraesCS5A02G440000.1	1226	1272
TraesCS5A02G440000.1	1021	1067
TraesCS5A02G440000.1	1073	1119
TraesCS5A02G440000.1	589	635
TraesCS5A02G440000.1	576	621
TraesCS5A02G440000.1	1023	1068
TraesCS5A02G440000.1	1055	1100
TraesCS5A02G440000.1	1072	1117
TraesCS5A02G440000.1	1191	1236
TraesCS5A02G440000.1	1211	1256
TraesCS5A02G440000.1	1567	1612
TraesCS5A02G440000.1	576	621
TraesCS5A02G440000.1	1023	1068
TraesCS5A02G440000.1	1055	1100
TraesCS5A02G440000.1	1072	1117
TraesCS5A02G440000.1	1191	1236

TraesCS5A02G440000.1	1211	1256
TraesCS5A02G440000.1	1567	1612
TraesCS5A02G440000.1	750	796
TraesCS5B02G011200.1	56	103
TraesCS5B02G011200.1	108	157
TraesCS5B02G011200.1	709	755
TraesCS5B02G011200.1	815	863
TraesCS5B02G011200.1	858	904
TraesCS5B02G011200.1	1064	1113
TraesCS5B02G011200.1	1097	1142
TraesCS5B02G011200.1	1099	1144
TraesCS5B02G011200.1	1138	1185
TraesCS5B02G011200.1	1539	1590
TraesCS5B02G011200.1	1730	1775
TraesCS5B02G011200.1	1752	1797
TraesCS5B02G011200.1	1810	1855
TraesCS5B02G011200.1	1912	1957
TraesCS5B02G011200.1	1957	2003
TraesCS5B02G066300.1	1432	1477
TraesCS5B02G066300.1	1463	1508
TraesCS5B02G066300.1	1431	1477
TraesCS5B02G066300.1	1462	1508
TraesCS5B02G066300.1	1639	1685
TraesCS5B02G066300.1	1730	1776
TraesCS5B02G066300.1	645	693
TraesCS5B02G066300.1	110	155
TraesCS5B02G066300.1	110	155
TraesCS5B02G066300.1	294	343
TraesCS5B02G066300.1	1118	1168
TraesCS5B02G066300.1	1459	1508
TraesCS5B02G181500.1	1476	1521
TraesCS5B02G181500.1	1620	1665
TraesCS5B02G181500.1	1779	1828
TraesCS5B02G181500.1	1365	1411
TraesCS5B02G181500.1	1510	1556
TraesCS5B02G181500.1	382	429
TraesCS5B02G181500.1	1476	1522
TraesCS5B02G181500.1	1619	1665
TraesCS5B02G181500.1	366	413
TraesCS5B02G181500.1	1657	1704
TraesCS5B02G181500.1	291	339
TraesCS5B02G181500.1	1278	1324
TraesCS5B02G181500.1	1670	1717
TraesCS5B02G181500.1	1671	1717
TraesCS5B02G181500.1	177	222
TraesCS5B02G181500.1	202	247
TraesCS5B02G181500.1	205	250
TraesCS5B02G181500.1	1469	1514
TraesCS5B02G181500.1	177	222
TraesCS5B02G181500.1	202	247
TraesCS5B02G181500.1	205	250
TraesCS5B02G181500.1	1469	1514
TraesCS5B02G181500.1	741	790
TraesCS5B02G442600.1	741	786
TraesCS5B02G442600.1	1320	1369
TraesCS5B02G442600.1	1342	1389
TraesCS5B02G442600.1	682	731
TraesCS5B02G442600.1	741	787

TraesCS5B02G442600.1	117	163
TraesCS5B02G442600.1	1795	1842
TraesCS5B02G442600.1	1368	1414
TraesCS5B02G442600.1	1672	1719
TraesCS5B02G442600.1	1877	1924
TraesCS5B02G442600.1	187	233
TraesCS5B02G442600.1	368	414
TraesCS5B02G442600.1	708	754
TraesCS5B02G442600.1	1059	1105
TraesCS5B02G442600.1	-17	28
TraesCS5B02G442600.1	200	245
TraesCS5B02G442600.1	257	302
TraesCS5B02G442600.1	381	426
TraesCS5B02G442600.1	603	648
TraesCS5B02G442600.1	632	677
TraesCS5B02G442600.1	1423	1468
TraesCS5B02G442600.1	1552	1597
TraesCS5B02G442600.1	-17	28
TraesCS5B02G442600.1	200	245
TraesCS5B02G442600.1	257	302
TraesCS5B02G442600.1	381	426
TraesCS5B02G442600.1	603	648
TraesCS5B02G442600.1	632	677
TraesCS5B02G442600.1	1423	1468
TraesCS5B02G442600.1	1552	1597
TraesCS5B02G442600.1	502	548
TraesCS5B02G442600.1	1399	1448
TraesCS5B02G442700.1	162	207
TraesCS5B02G442700.1	222	267
TraesCS5B02G442700.1	624	669
TraesCS5B02G442700.1	1206	1255
TraesCS5B02G442700.1	1322	1367
TraesCS5B02G442700.1	1377	1422
TraesCS5B02G442700.1	379	425
TraesCS5B02G442700.1	1236	1285
TraesCS5B02G442700.1	161	207
TraesCS5B02G442700.1	623	669
TraesCS5B02G442700.1	1322	1368
TraesCS5B02G442700.1	221	267
TraesCS5B02G442700.1	236	282
TraesCS5B02G442700.1	1377	1423
TraesCS5B02G442700.1	1412	1458
TraesCS5B02G442700.1	1852	1899
TraesCS5B02G442700.1	1614	1661
TraesCS5B02G442700.1	647	693
TraesCS5B02G442700.1	157	202
TraesCS5B02G442700.1	225	270
TraesCS5B02G442700.1	333	378
TraesCS5B02G442700.1	741	786
TraesCS5B02G442700.1	829	874
TraesCS5B02G442700.1	966	1011
TraesCS5B02G442700.1	1011	1056
TraesCS5B02G442700.1	157	202
TraesCS5B02G442700.1	225	270
TraesCS5B02G442700.1	333	378
TraesCS5B02G442700.1	741	786
TraesCS5B02G442700.1	829	874
TraesCS5B02G442700.1	966	1011

TraesCS5B02G442700.1	1011	1056
TraesCS5B02G442700.1	1304	1350
TraesCS5B02G442800.1	138	187
TraesCS5B02G442800.1	1348	1393
TraesCS5B02G442800.1	1541	1586
TraesCS5B02G442800.1	1626	1675
TraesCS5B02G442800.1	1628	1673
TraesCS5B02G442800.1	1694	1740
TraesCS5B02G442800.1	1695	1740
TraesCS5B02G442800.1	1551	1597
TraesCS5B02G442800.1	1458	1507
TraesCS5B02G442800.1	272	318
TraesCS5B02G442800.1	387	433
TraesCS5B02G442800.1	939	985
TraesCS5B02G442800.1	16	65
TraesCS5B02G442800.1	1538	1587
TraesCS5B02G442800.1	1541	1587
TraesCS5B02G442800.1	1628	1674
TraesCS5B02G442800.1	1694	1740
TraesCS5B02G442800.1	1348	1394
TraesCS5B02G442800.1	1694	1740
TraesCS5B02G442800.1	1462	1508
TraesCS5B02G442800.1	174	224
TraesCS5B02G442800.1	644	690
TraesCS5B02G442800.1	744	789
TraesCS5B02G442800.1	1647	1692
TraesCS5B02G442800.1	744	789
TraesCS5B02G442800.1	1647	1692
TraesCS5B02G442800.1	1274	1320
TraesCS5B02G442800.1	1056	1105
TraesCS5B02G442900.1	877	922
TraesCS5B02G442900.1	1074	1119
TraesCS5B02G442900.1	1507	1552
TraesCS5B02G442900.1	1583	1628
TraesCS5B02G442900.1	1624	1669
TraesCS5B02G442900.1	1731	1777
TraesCS5B02G442900.1	1732	1777
TraesCS5B02G442900.1	1739	1785
TraesCS5B02G442900.1	1740	1785
TraesCS5B02G442900.1	1783	1832
TraesCS5B02G442900.1	1785	1831
TraesCS5B02G442900.1	1786	1831
TraesCS5B02G442900.1	1476	1522
TraesCS5B02G442900.1	178	224
TraesCS5B02G442900.1	718	765
TraesCS5B02G442900.1	1506	1552
TraesCS5B02G442900.1	1583	1631
TraesCS5B02G442900.1	1623	1669
TraesCS5B02G442900.1	1731	1777
TraesCS5B02G442900.1	1739	1785
TraesCS5B02G442900.1	1785	1831
TraesCS5B02G442900.1	877	923
TraesCS5B02G442900.1	1074	1120
TraesCS5B02G442900.1	1583	1629
TraesCS5B02G442900.1	1731	1777
TraesCS5B02G442900.1	1739	1785
TraesCS5B02G442900.1	1785	1831
TraesCS5B02G442900.1	120	167

TraesCS5B02G442900.1	271	317
TraesCS5B02G442900.1	804	856
TraesCS5B02G442900.1	225	271
TraesCS5B02G442900.1	424	470
TraesCS5B02G442900.1	1503	1549
TraesCS5B02G442900.1	22	71
TraesCS5B02G442900.1	146	195
TraesCS5B02G442900.1	184	231
TraesCS5B02G442900.1	1132	1182
TraesCS5B02G442900.1	418	469
TraesCS5B02G442900.1	1160	1206
TraesCS5B02G442900.1	1056	1102
TraesCS5B02G443000.1	12	57
TraesCS5B02G443000.1	315	360
TraesCS5B02G443000.1	388	437
TraesCS5B02G443000.1	784	829
TraesCS5B02G443000.1	836	881
TraesCS5B02G443000.1	1083	1128
TraesCS5B02G443000.1	1596	1641
TraesCS5B02G443000.1	1637	1682
TraesCS5B02G443000.1	1783	1832
TraesCS5B02G443000.1	1785	1831
TraesCS5B02G443000.1	1786	1831
TraesCS5B02G443000.1	1489	1535
TraesCS5B02G443000.1	740	789
TraesCS5B02G443000.1	11	57
TraesCS5B02G443000.1	315	361
TraesCS5B02G443000.1	1083	1129
TraesCS5B02G443000.1	1596	1642
TraesCS5B02G443000.1	1785	1831
TraesCS5B02G443000.1	784	830
TraesCS5B02G443000.1	835	881
TraesCS5B02G443000.1	1636	1682
TraesCS5B02G443000.1	1785	1831
TraesCS5B02G443000.1	1141	1191
TraesCS5B02G443000.1	1516	1562
TraesCS5B02G443000.1	345	395
TraesCS5B02G443000.1	1347	1397
TraesCS5B02G443000.1	1446	1499
TraesCS5B02G443000.1	1702	1750
TraesCS5B02G443000.1	54	100
TraesCS5B02G443000.1	1169	1215
TraesCS5B02G443000.1	546	592
TraesCS5B02G443000.1	1750	1796
TraesCS5B02G443000.1	1697	1742
TraesCS5B02G443000.1	1697	1742
TraesCS5B02G443100.1	-14	31
TraesCS5B02G443100.1	59	108
TraesCS5B02G443100.1	480	525
TraesCS5B02G443100.1	532	577
TraesCS5B02G443100.1	779	824
TraesCS5B02G443100.1	1216	1261
TraesCS5B02G443100.1	1292	1337
TraesCS5B02G443100.1	1333	1378
TraesCS5B02G443100.1	1783	1832
TraesCS5B02G443100.1	1785	1831
TraesCS5B02G443100.1	1786	1831
TraesCS5B02G443100.1	1185	1231

TraesCS5B02G443100.1	436	485
TraesCS5B02G443100.1	480	526
TraesCS5B02G443100.1	531	577
TraesCS5B02G443100.1	1215	1261
TraesCS5B02G443100.1	1332	1378
TraesCS5B02G443100.1	1785	1831
TraesCS5B02G443100.1	-14	32
TraesCS5B02G443100.1	779	825
TraesCS5B02G443100.1	1292	1338
TraesCS5B02G443100.1	1785	1831
TraesCS5B02G443100.1	1010	1058
TraesCS5B02G443100.1	1600	1650
TraesCS5B02G443100.1	837	887
TraesCS5B02G443100.1	1212	1258
TraesCS5B02G443100.1	1142	1195
TraesCS5B02G443100.1	242	288
TraesCS5B02G443100.1	1750	1796
TraesCS5B02G443100.1	865	911
TraesCS5B02G443100.1	1696	1741
TraesCS5B02G443100.1	1696	1741
TraesCS5B02G443100.1	1601	1650
TraesCS5B02G443200.1	929	974
TraesCS5B02G443200.1	1367	1412
TraesCS5B02G443200.1	1445	1490
TraesCS5B02G443200.1	1611	1656
TraesCS5B02G443200.1	579	626
TraesCS5B02G443200.1	88	135
TraesCS5B02G443200.1	1366	1412
TraesCS5B02G443200.1	1610	1656
TraesCS5B02G443200.1	929	975
TraesCS5B02G443200.1	1445	1491
TraesCS5B02G443200.1	1192	1242
TraesCS5B02G443200.1	987	1037
TraesCS5B02G443200.1	-2	44
TraesCS5B02G443200.1	283	329
TraesCS5B02G443200.1	1363	1409
TraesCS5B02G443200.1	277	327
TraesCS5B02G443200.1	1015	1061
TraesCS5B02G443200.1	1691	1736
TraesCS5B02G443200.1	1691	1736
TraesCS5B02G443200.1	1290	1336
TraesCS5B02G443300.1	1054	1099
TraesCS5B02G443300.1	1492	1537
TraesCS5B02G443300.1	1570	1615
TraesCS5B02G443300.1	1611	1656
TraesCS5B02G443300.1	705	752
TraesCS5B02G443300.1	71	118
TraesCS5B02G443300.1	1491	1537
TraesCS5B02G443300.1	1610	1656
TraesCS5B02G443300.1	1054	1100
TraesCS5B02G443300.1	1570	1616
TraesCS5B02G443300.1	1112	1162
TraesCS5B02G443300.1	408	454
TraesCS5B02G443300.1	1488	1534
TraesCS5B02G443300.1	402	452
TraesCS5B02G443300.1	1140	1186
TraesCS5B02G443300.1	1691	1736
TraesCS5B02G443300.1	1691	1736

TraesCS5B02G443300.1	1294	1343
TraesCS5B02G443400.1	1053	1098
TraesCS5B02G443400.1	1570	1615
TraesCS5B02G443400.1	1611	1656
TraesCS5B02G443400.1	414	464
TraesCS5B02G443400.1	703	750
TraesCS5B02G443400.1	69	116
TraesCS5B02G443400.1	1610	1656
TraesCS5B02G443400.1	1053	1099
TraesCS5B02G443400.1	1570	1616
TraesCS5B02G443400.1	1317	1367
TraesCS5B02G443400.1	406	452
TraesCS5B02G443400.1	1111	1161
TraesCS5B02G443400.1	400	450
TraesCS5B02G443400.1	1139	1185
TraesCS5B02G443400.1	1691	1736
TraesCS5B02G443400.1	1691	1736
TraesCS5B02G443400.1	1415	1461
TraesCS5B02G443400.1	1245	1294
TraesCS5B02G443400.1	1293	1342
TraesCS5B02G443500.1	1053	1098
TraesCS5B02G443500.1	1570	1615
TraesCS5B02G443500.1	1611	1656
TraesCS5B02G443500.1	703	750
TraesCS5B02G443500.1	69	116
TraesCS5B02G443500.1	1610	1656
TraesCS5B02G443500.1	1053	1099
TraesCS5B02G443500.1	1570	1616
TraesCS5B02G443500.1	1317	1367
TraesCS5B02G443500.1	1111	1161
TraesCS5B02G443500.1	406	452
TraesCS5B02G443500.1	400	450
TraesCS5B02G443500.1	1139	1185
TraesCS5B02G443500.1	1691	1736
TraesCS5B02G443500.1	1691	1736
TraesCS5B02G443500.1	1415	1461
TraesCS5B02G443500.1	1245	1294
TraesCS5B02G443500.1	1293	1342
TraesCS5B02G443600.1	1124	1169
TraesCS5B02G443600.1	1561	1606
TraesCS5B02G443600.1	1639	1684
TraesCS5B02G443600.1	1680	1725
TraesCS5B02G443600.1	775	822
TraesCS5B02G443600.1	140	187
TraesCS5B02G443600.1	205	252
TraesCS5B02G443600.1	1124	1170
TraesCS5B02G443600.1	1639	1685
TraesCS5B02G443600.1	1560	1606
TraesCS5B02G443600.1	1679	1725
TraesCS5B02G443600.1	478	524
TraesCS5B02G443600.1	1557	1603
TraesCS5B02G443600.1	1182	1232
TraesCS5B02G443600.1	1209	1255
TraesCS5B02G443600.1	472	522
TraesCS5B02G443600.1	1760	1805
TraesCS5B02G443600.1	1760	1805
TraesCS5B02G443600.1	1363	1412
TraesCS5B02G443700.1	1053	1098

TraesCS5B02G443700.1	1570	1615
TraesCS5B02G443700.1	1611	1656
TraesCS5B02G443700.1	703	750
TraesCS5B02G443700.1	68	115
TraesCS5B02G443700.1	1053	1099
TraesCS5B02G443700.1	1570	1616
TraesCS5B02G443700.1	1610	1656
TraesCS5B02G443700.1	406	452
TraesCS5B02G443700.1	1111	1161
TraesCS5B02G443700.1	1317	1367
TraesCS5B02G443700.1	1139	1185
TraesCS5B02G443700.1	400	450
TraesCS5B02G443700.1	1691	1736
TraesCS5B02G443700.1	1691	1736
TraesCS5B02G443700.1	1415	1461
TraesCS5B02G443700.1	1245	1294
TraesCS5B02G443700.1	1293	1342
TraesCS5B02G443800.1	1077	1122
TraesCS5B02G443800.1	1516	1561
TraesCS5B02G443800.1	1594	1639
TraesCS5B02G443800.1	1635	1680
TraesCS5B02G443800.1	727	774
TraesCS5B02G443800.1	93	140
TraesCS5B02G443800.1	1077	1123
TraesCS5B02G443800.1	1594	1640
TraesCS5B02G443800.1	1515	1561
TraesCS5B02G443800.1	1634	1680
TraesCS5B02G443800.1	1135	1185
TraesCS5B02G443800.1	430	476
TraesCS5B02G443800.1	1512	1558
TraesCS5B02G443800.1	1163	1209
TraesCS5B02G443800.1	424	474
TraesCS5B02G443800.1	1695	1740
TraesCS5B02G443800.1	1695	1740
TraesCS5B02G443800.1	1438	1484
TraesCS5B02G443800.1	1317	1366
TraesCS5D02G446800.1	1336	1381
TraesCS5D02G446800.1	1505	1550
TraesCS5D02G446800.1	552	601
TraesCS5D02G446800.1	905	951
TraesCS5D02G446800.1	967	1013
TraesCS5D02G446800.1	1470	1516
TraesCS5D02G446800.1	458	504
TraesCS5D02G446800.1	1290	1336
TraesCS5D02G446800.1	1505	1551
TraesCS5D02G446800.1	1336	1382
TraesCS5D02G446800.1	1073	1120
TraesCS5D02G446800.1	1794	1841
TraesCS5D02G446800.1	556	602
TraesCS5D02G446800.1	1644	1691
TraesCS5D02G446800.1	860	910
TraesCS5D02G446800.1	802	849
TraesCS5D02G446800.1	867	912
TraesCS5D02G446800.1	962	1007
TraesCS5D02G446800.1	1035	1080
TraesCS5D02G446800.1	1278	1323
TraesCS5D02G446800.1	1436	1481
TraesCS5D02G446800.1	867	912

TraesCS5D02G446800.1	962	1007
TraesCS5D02G446800.1	1035	1080
TraesCS5D02G446800.1	1278	1323
TraesCS5D02G446800.1	1436	1481
TraesCS5D02G446800.1	1421	1470
TraesCS5D02G446900.1	260	307
TraesCS5D02G446900.1	1339	1384
TraesCS5D02G446900.1	1478	1527
TraesCS5D02G446900.1	1173	1222
TraesCS5D02G446900.1	1434	1480
TraesCS5D02G446900.1	1339	1385
TraesCS5D02G446900.1	256	306
TraesCS5D02G446900.1	1654	1701
TraesCS5D02G446900.1	119	165
TraesCS5D02G446900.1	188	234
TraesCS5D02G446900.1	526	572
TraesCS5D02G446900.1	357	402
TraesCS5D02G446900.1	467	512
TraesCS5D02G446900.1	1289	1334
TraesCS5D02G446900.1	357	402
TraesCS5D02G446900.1	467	512
TraesCS5D02G446900.1	1289	1334
TraesCS5D02G446900.1	1249	1298
TraesCS5D02G446900.1	485	534
TraesCS5D02G446900.1	1794	1842
TraesCS5D02G447000.1	-6	39
TraesCS5D02G447000.1	195	240
TraesCS5D02G447000.1	1232	1277
TraesCS5D02G447000.1	1518	1563
TraesCS5D02G447000.1	1671	1717
TraesCS5D02G447000.1	1672	1717
TraesCS5D02G447000.1	526	573
TraesCS5D02G447000.1	1528	1574
TraesCS5D02G447000.1	1500	1546
TraesCS5D02G447000.1	194	240
TraesCS5D02G447000.1	1175	1221
TraesCS5D02G447000.1	1231	1277
TraesCS5D02G447000.1	1515	1564
TraesCS5D02G447000.1	1518	1564
TraesCS5D02G447000.1	1671	1717
TraesCS5D02G447000.1	-6	40
TraesCS5D02G447000.1	1671	1717
TraesCS5D02G447000.1	1289	1338
TraesCS5D02G447000.1	1168	1214
TraesCS5D02G447000.1	1081	1127
TraesCS5D02G447000.1	1064	1111
TraesCS5D02G447000.1	839	884
TraesCS5D02G447000.1	1624	1669
TraesCS5D02G447000.1	839	884
TraesCS5D02G447000.1	1624	1669
TraesCS5D02G447000.1	1122	1168
TraesCS5D02G447000.1	1435	1481
TraesCS5D02G447000.1	162	212
TraesCS5D02G447000.1	116	165
TraesCS5D02G447000.1	556	605
TraesCS5D02G447100.1	877	925
TraesCS5D02G447100.1	879	924
TraesCS5D02G447100.1	24	71

TraesCS5D02G447100.1	42	89
TraesCS5D02G447100.1	1442	1488
TraesCS5D02G447100.1	1857	1906
TraesCS5D02G447100.1	831	877
TraesCS5D02G447100.1	16	63
TraesCS5D02G447100.1	1495	1542
TraesCS5D02G447100.1	879	925
TraesCS5D02G447100.1	1029	1075
TraesCS5D02G447100.1	203	250
TraesCS5D02G447100.1	167	213
TraesCS5D02G447100.1	171	217
TraesCS5D02G447100.1	175	221
TraesCS5D02G447100.1	189	235
TraesCS5D02G447100.1	230	276
TraesCS5D02G447100.1	245	291
TraesCS5D02G447100.1	266	312
TraesCS5D02G447100.1	271	317
TraesCS5D02G447100.1	703	749
TraesCS5D02G447100.1	716	762
TraesCS5D02G447100.1	721	767
TraesCS5D02G447100.1	741	787
TraesCS5D02G447100.1	890	936
TraesCS5D02G447100.1	980	1026
TraesCS5D02G447100.1	1065	1110
TraesCS5D02G447100.1	1383	1428
TraesCS5D02G447100.1	1539	1584
TraesCS5D02G447100.1	1065	1110
TraesCS5D02G447100.1	1383	1428
TraesCS5D02G447100.1	1539	1584
TraesCS5D02G447100.1	36	85
TraesCS5D02G447100.1	1235	1285
TraesCS5D02G447100.1	1631	1680
TraesCS6A02G345000.1	411	460
TraesCS6A02G345000.1	947	992
TraesCS6A02G345000.1	1230	1275
TraesCS6A02G345000.1	1930	1975
TraesCS6A02G345000.1	738	785
TraesCS6A02G345000.1	1734	1781
TraesCS6A02G345000.1	424	470
TraesCS6A02G345000.1	946	992
TraesCS6A02G345000.1	1229	1275
TraesCS6A02G345000.1	1930	1976
TraesCS6A02G345000.1	1029	1078
TraesCS6A02G345000.1	334	380
TraesCS6A02G345000.1	218	266
TraesCS6A02G345000.1	803	849
TraesCS6A02G345000.1	1318	1364
TraesCS6A02G345000.1	684	731
TraesCS6A02G345000.1	1478	1526
TraesCS6A02G345000.1	669	715
TraesCS6A02G345000.1	81	126
TraesCS6A02G345000.1	417	462
TraesCS6A02G345000.1	1228	1273
TraesCS6A02G345000.1	1653	1698
TraesCS6A02G345000.1	1907	1952
TraesCS6A02G345000.1	81	126
TraesCS6A02G345000.1	417	462
TraesCS6A02G345000.1	1228	1273

TraesCS6A02G345000.1	1653	1698
TraesCS6A02G345000.1	1907	1952
TraesCS6A02G345100.1	590	639
TraesCS6A02G345100.1	737	782
TraesCS6A02G345100.1	763	808
TraesCS6A02G345100.1	1642	1687
TraesCS6A02G345100.1	480	528
TraesCS6A02G345100.1	1864	1910
TraesCS6A02G345100.1	1578	1624
TraesCS6A02G345100.1	1009	1056
TraesCS6A02G345100.1	92	138
TraesCS6A02G345100.1	761	810
TraesCS6A02G345100.1	762	808
TraesCS6A02G345100.1	808	854
TraesCS6A02G345100.1	1642	1688
TraesCS6A02G345100.1	737	783
TraesCS6A02G345100.1	1137	1184
TraesCS6A02G345100.1	290	338
TraesCS6A02G345100.1	-11	35
TraesCS6A02G345100.1	484	530
TraesCS6A02G345100.1	162	211
TraesCS6A02G345100.1	1749	1795
TraesCS6A02G345100.1	375	420
TraesCS6A02G345100.1	480	525
TraesCS6A02G345100.1	375	420
TraesCS6A02G345100.1	480	525
TraesCS6A02G345100.1	685	734
TraesCS6A02G345200.1	320	365
TraesCS6A02G345200.1	923	971
TraesCS6A02G345200.1	925	970
TraesCS6A02G345200.1	1018	1067
TraesCS6A02G345200.1	1020	1065
TraesCS6A02G345200.1	1075	1120
TraesCS6A02G345200.1	1100	1145
TraesCS6A02G345200.1	1483	1528
TraesCS6A02G345200.1	1637	1683
TraesCS6A02G345200.1	1736	1782
TraesCS6A02G345200.1	435	482
TraesCS6A02G345200.1	317	366
TraesCS6A02G345200.1	1018	1067
TraesCS6A02G345200.1	319	365
TraesCS6A02G345200.1	925	971
TraesCS6A02G345200.1	1020	1066
TraesCS6A02G345200.1	1075	1121
TraesCS6A02G345200.1	1100	1146
TraesCS6A02G345200.1	1482	1528
TraesCS6A02G345200.1	1581	1627
TraesCS6A02G345200.1	1711	1757
TraesCS6A02G345200.1	1350	1396
TraesCS6A02G345200.1	39	84
TraesCS6A02G345200.1	323	368
TraesCS6A02G345200.1	1077	1122
TraesCS6A02G345200.1	39	84
TraesCS6A02G345200.1	323	368
TraesCS6A02G345200.1	1077	1122
TraesCS6A02G345200.1	1604	1650
TraesCS6A02G345200.1	1357	1406
TraesCS6A02G346300.1	386	431

TraesCS6A02G346300.1	1079	1124
TraesCS6A02G346300.1	1666	1715
TraesCS6A02G346300.1	1687	1732
TraesCS6A02G346300.1	242	289
TraesCS6A02G346300.1	728	775
TraesCS6A02G346300.1	807	854
TraesCS6A02G346300.1	565	611
TraesCS6A02G346300.1	601	647
TraesCS6A02G346300.1	1818	1864
TraesCS6A02G346300.1	1079	1125
TraesCS6A02G346300.1	385	431
TraesCS6A02G346300.1	1686	1732
TraesCS6A02G346300.1	1825	1871
TraesCS6A02G346300.1	329	376
TraesCS6A02G346300.1	625	675
TraesCS6A02G346300.1	131	178
TraesCS6A02G346300.1	587	633
TraesCS6A02G346300.1	1656	1702
TraesCS6A02G346300.1	1914	1961
TraesCS6A02G346300.1	1915	1961
TraesCS6A02G346300.1	988	1033
TraesCS6A02G346300.1	1024	1069
TraesCS6A02G346300.1	988	1033
TraesCS6A02G346300.1	1024	1069
TraesCS6A02G346300.1	682	728
TraesCS6A02G346300.1	971	1020
TraesCS6B02G377700.1	807	853
TraesCS6B02G377700.1	1453	1499
TraesCS6B02G377700.1	350	396
TraesCS6B02G377700.1	479	525
TraesCS6B02G377700.1	1739	1786
TraesCS6B02G377700.1	224	270
TraesCS6B02G377700.1	286	332
TraesCS6B02G377700.1	151	198
TraesCS6B02G377700.1	587	633
TraesCS6B02G377700.1	1528	1576
TraesCS6B02G377700.1	1167	1212
TraesCS6B02G377700.1	1658	1703
TraesCS6B02G377700.1	1909	1954
TraesCS6B02G377700.1	1167	1212
TraesCS6B02G377700.1	1658	1703
TraesCS6B02G377700.1	1909	1954
TraesCS6B02G377800.1	924	969
TraesCS6B02G377800.1	1376	1421
TraesCS6B02G377800.1	1649	1694
TraesCS6B02G377800.1	1661	1706
TraesCS6B02G377800.1	1003	1049
TraesCS6B02G377800.1	1634	1680
TraesCS6B02G377800.1	1873	1919
TraesCS6B02G377800.1	1186	1235
TraesCS6B02G377800.1	1588	1634
TraesCS6B02G377800.1	1794	1840
TraesCS6B02G377800.1	1312	1359
TraesCS6B02G377800.1	1660	1706
TraesCS6B02G377800.1	923	969
TraesCS6B02G377800.1	1375	1421
TraesCS6B02G377800.1	1649	1695
TraesCS6B02G377800.1	1598	1644

TraesCS6B02G377800.1	910	959
TraesCS6B02G377800.1	911	961
TraesCS6B02G377800.1	1569	1616
TraesCS6B02G377800.1	1570	1616
TraesCS6B02G377800.1	602	648
TraesCS6B02G377800.1	1758	1804
TraesCS6B02G377800.1	922	967
TraesCS6B02G377800.1	1369	1414
TraesCS6B02G377800.1	1374	1419
TraesCS6B02G377800.1	922	967
TraesCS6B02G377800.1	1369	1414
TraesCS6B02G377800.1	1374	1419
TraesCS6B02G377800.1	12	61
TraesCS6B02G377800.1	849	898
TraesCS6B02G378000.1	1102	1150
TraesCS6B02G378000.1	1104	1149
TraesCS6B02G378000.1	1198	1247
TraesCS6B02G378000.1	1200	1245
TraesCS6B02G378000.1	1258	1303
TraesCS6B02G378000.1	1283	1328
TraesCS6B02G378000.1	1665	1711
TraesCS6B02G378000.1	182	229
TraesCS6B02G378000.1	1198	1247
TraesCS6B02G378000.1	1104	1150
TraesCS6B02G378000.1	1200	1246
TraesCS6B02G378000.1	1258	1304
TraesCS6B02G378000.1	1283	1329
TraesCS6B02G378000.1	1713	1759
TraesCS6B02G378000.1	973	1020
TraesCS6B02G378000.1	386	436
TraesCS6B02G378000.1	388	436
TraesCS6B02G378000.1	1511	1557
TraesCS6B02G378000.1	674	721
TraesCS6B02G378000.1	1153	1199
TraesCS6B02G378000.1	98	143
TraesCS6B02G378000.1	125	170
TraesCS6B02G378000.1	1260	1305
TraesCS6B02G378000.1	1367	1412
TraesCS6B02G378000.1	98	143
TraesCS6B02G378000.1	125	170
TraesCS6B02G378000.1	1260	1305
TraesCS6B02G378000.1	1367	1412
TraesCS6B02G378000.1	1140	1186
TraesCS6B02G378000.1	556	605
TraesCS6B02G378000.1	63	112
TraesCS6B02G378000.1	1518	1567
TraesCS6B02G379800.1	425	470
TraesCS6B02G379800.1	573	618
TraesCS6B02G379800.1	1602	1647
TraesCS6B02G379800.1	1668	1717
TraesCS6B02G379800.1	456	502
TraesCS6B02G379800.1	341	390
TraesCS6B02G379800.1	425	471
TraesCS6B02G379800.1	1601	1647
TraesCS6B02G379800.1	572	618
TraesCS6B02G379800.1	1810	1856
TraesCS6B02G379800.1	1863	1909
TraesCS6B02G379800.1	612	658

TraesCS6B02G379800.1	238	283
TraesCS6B02G379800.1	830	875
TraesCS6B02G379800.1	238	283
TraesCS6B02G379800.1	830	875
TraesCS6B02G379800.1	996	1042
TraesCS6D02G327500.1	92	137
TraesCS6D02G327500.1	136	181
TraesCS6D02G327500.1	859	904
TraesCS6D02G327500.1	908	953
TraesCS6D02G327500.1	1006	1051
TraesCS6D02G327500.1	1033	1079
TraesCS6D02G327500.1	1034	1079
TraesCS6D02G327500.1	917	964
TraesCS6D02G327500.1	1588	1637
TraesCS6D02G327500.1	429	475
TraesCS6D02G327500.1	481	527
TraesCS6D02G327500.1	511	557
TraesCS6D02G327500.1	601	647
TraesCS6D02G327500.1	651	697
TraesCS6D02G327500.1	679	725
TraesCS6D02G327500.1	806	852
TraesCS6D02G327500.1	1740	1787
TraesCS6D02G327500.1	1005	1051
TraesCS6D02G327500.1	1033	1079
TraesCS6D02G327500.1	92	138
TraesCS6D02G327500.1	136	182
TraesCS6D02G327500.1	858	904
TraesCS6D02G327500.1	907	953
TraesCS6D02G327500.1	1032	1080
TraesCS6D02G327500.1	1033	1079
TraesCS6D02G327500.1	1059	1109
TraesCS6D02G327500.1	1211	1259
TraesCS6D02G327500.1	1326	1372
TraesCS6D02G327500.1	1659	1704
TraesCS6D02G327500.1	1909	1954
TraesCS6D02G327500.1	1659	1704
TraesCS6D02G327500.1	1909	1954
TraesCS6D02G327500.1	1243	1292
TraesCS6D02G327500.1	1801	1850
TraesCS6D02G327600.1	613	658
TraesCS6D02G327600.1	832	877
TraesCS6D02G327600.1	855	900
TraesCS6D02G327600.1	1051	1096
TraesCS6D02G327600.1	1373	1418
TraesCS6D02G327600.1	1641	1686
TraesCS6D02G327600.1	1418	1464
TraesCS6D02G327600.1	1859	1905
TraesCS6D02G327600.1	844	890
TraesCS6D02G327600.1	1780	1826
TraesCS6D02G327600.1	1265	1312
TraesCS6D02G327600.1	12	58
TraesCS6D02G327600.1	173	219
TraesCS6D02G327600.1	613	659
TraesCS6D02G327600.1	663	709
TraesCS6D02G327600.1	853	902
TraesCS6D02G327600.1	854	900
TraesCS6D02G327600.1	900	946
TraesCS6D02G327600.1	1051	1097

TraesCS6D02G327600.1	1372	1418
TraesCS6D02G327600.1	832	878
TraesCS6D02G327600.1	1640	1686
TraesCS6D02G327600.1	199	245
TraesCS6D02G327600.1	304	350
TraesCS6D02G327600.1	1741	1787
TraesCS6D02G327600.1	46	91
TraesCS6D02G327600.1	365	410
TraesCS6D02G327600.1	1366	1411
TraesCS6D02G327600.1	1371	1416
TraesCS6D02G327600.1	46	91
TraesCS6D02G327600.1	365	410
TraesCS6D02G327600.1	1366	1411
TraesCS6D02G327600.1	1371	1416
TraesCS6D02G327600.1	123	169
TraesCS6D02G327600.1	612	661
TraesCS6D02G327600.1	768	817
TraesCS6D02G327700.1	1037	1085
TraesCS6D02G327700.1	1039	1084
TraesCS6D02G327700.1	1093	1141
TraesCS6D02G327700.1	1095	1140
TraesCS6D02G327700.1	1149	1194
TraesCS6D02G327700.1	1179	1224
TraesCS6D02G327700.1	1541	1588
TraesCS6D02G327700.1	1562	1608
TraesCS6D02G327700.1	70	119
TraesCS6D02G327700.1	1686	1735
TraesCS6D02G327700.1	157	203
TraesCS6D02G327700.1	1039	1085
TraesCS6D02G327700.1	1095	1141
TraesCS6D02G327700.1	1149	1195
TraesCS6D02G327700.1	1179	1225
TraesCS6D02G327700.1	1737	1783
TraesCS6D02G327700.1	1769	1815
TraesCS6D02G327700.1	1394	1440
TraesCS6D02G327700.1	1657	1703
TraesCS6D02G327700.1	3	54
TraesCS6D02G327700.1	912	958
TraesCS6D02G327700.1	707	752
TraesCS6D02G327700.1	820	865
TraesCS6D02G327700.1	1151	1196
TraesCS6D02G327700.1	1257	1302
TraesCS6D02G327700.1	707	752
TraesCS6D02G327700.1	820	865
TraesCS6D02G327700.1	1151	1196
TraesCS6D02G327700.1	1257	1302
TraesCS6D02G329200.1	438	484
TraesCS6D02G329200.1	439	484
TraesCS6D02G329200.1	586	631
TraesCS6D02G329200.1	780	829
TraesCS6D02G329200.1	782	827
TraesCS6D02G329200.1	1669	1718
TraesCS6D02G329200.1	768	814
TraesCS6D02G329200.1	354	403
TraesCS6D02G329200.1	438	484
TraesCS6D02G329200.1	782	828
TraesCS6D02G329200.1	438	484
TraesCS6D02G329200.1	585	631

TraesCS6D02G329200.1	750	796
TraesCS6D02G329200.1	1829	1875
TraesCS6D02G329200.1	246	294
TraesCS6D02G329200.1	610	657
TraesCS6D02G329200.1	625	671
TraesCS6D02G329200.1	658	704
TraesCS6D02G329200.1	258	303
TraesCS6D02G329200.1	575	620
TraesCS6D02G329200.1	258	303
TraesCS6D02G329200.1	575	620
TraesCS7A02G152200.1	178	223
TraesCS7A02G152200.1	908	953
TraesCS7A02G152200.1	1849	1894
TraesCS7A02G152200.1	1928	1973
TraesCS7A02G152200.1	1971	2018
TraesCS7A02G152200.1	93	140
TraesCS7A02G152200.1	1189	1238
TraesCS7A02G152200.1	1250	1299
TraesCS7A02G152200.1	1721	1767
TraesCS7A02G152200.1	908	954
TraesCS7A02G152200.1	1848	1894
TraesCS7A02G152200.1	1928	1974
TraesCS7A02G152200.1	178	224
TraesCS7A02G152200.1	1256	1305
TraesCS7A02G152200.1	53	101
TraesCS7A02G152200.1	1844	1890
TraesCS7A02G152200.1	207	254
TraesCS7A02G152200.1	1822	1869
TraesCS7A02G152200.1	926	979
TraesCS7A02G152200.1	1443	1494
TraesCS7A02G152200.1	1930	1975
TraesCS7A02G152200.1	1930	1975
TraesCS7A02G198800.1	291	340
TraesCS7A02G198800.1	477	523
TraesCS7A02G198800.1	1671	1717
TraesCS7A02G198800.1	102	150
TraesCS7A02G198800.1	1659	1705
TraesCS7A02G198800.1	856	904
TraesCS7A02G198800.1	1597	1643
TraesCS7A02G198800.1	1629	1675
TraesCS7A02G198800.1	536	582
TraesCS7A02G198800.1	429	478
TraesCS7A02G198900.1	355	404
TraesCS7A02G198900.1	757	803
TraesCS7A02G198900.1	1220	1266
TraesCS7A02G198900.1	1908	1954
TraesCS7A02G198900.1	1083	1131
TraesCS7A02G198900.1	45	91
TraesCS7B02G056100.1	462	510
TraesCS7B02G056100.1	1213	1261
TraesCS7B02G056100.1	216	262
TraesCS7B02G056100.1	210	259
TraesCS7B02G104900.1	55	101
TraesCS7B02G104900.1	56	101
TraesCS7B02G104900.1	978	1025
TraesCS7B02G104900.1	1143	1188
TraesCS7B02G104900.1	1905	1950
TraesCS7B02G104900.1	1530	1576

TraesCS7B02G104900.1	1669	1715
TraesCS7B02G104900.1	896	943
TraesCS7B02G104900.1	55	101
TraesCS7B02G104900.1	1143	1189
TraesCS7B02G104900.1	55	101
TraesCS7B02G104900.1	301	347
TraesCS7B02G104900.1	1905	1951
TraesCS7B02G104900.1	202	249
TraesCS7B02G104900.1	264	310
TraesCS7B02G104900.1	751	797
TraesCS7B02G104900.1	1656	1702
TraesCS7B02G104900.1	502	550
TraesCS7B02G104900.1	251	300
TraesCS7B02G104900.1	1513	1562
TraesCS7B02G104900.1	369	417
TraesCS7B02G104900.1	1367	1415
TraesCS7B02G104900.1	1594	1640
TraesCS7B02G104900.1	1626	1672
TraesCS7B02G104900.1	1175	1220
TraesCS7B02G104900.1	1175	1220
TraesCS7B02G104900.1	1881	1930
TraesCS7B02G104900.1	713	762
TraesCS7B02G105000.1	1450	1495
TraesCS7B02G105000.1	1738	1783
TraesCS7B02G105000.1	1878	1923
TraesCS7B02G105000.1	234	280
TraesCS7B02G105000.1	1449	1495
TraesCS7B02G105000.1	1535	1581
TraesCS7B02G105000.1	1737	1783
TraesCS7B02G105000.1	1878	1924
TraesCS7B02G105000.1	11	57
TraesCS7B02G105000.1	221	267
TraesCS7B02G105000.1	78	127
TraesCS7B02G105000.1	1703	1751
TraesCS7B02G105000.1	159	205
TraesCS7B02G105000.1	191	237
TraesCS7B02G105000.1	1755	1801
TraesCS7B02G105000.1	1471	1516
TraesCS7B02G105000.1	1569	1614
TraesCS7B02G105000.1	1471	1516
TraesCS7B02G105000.1	1569	1614
TraesCS7B02G105000.1	1690	1736
TraesCS7B02G105000.1	1854	1903
TraesCS7B02G105100.1	256	301
TraesCS7B02G105100.1	1905	1950
TraesCS7B02G105100.1	1747	1796
TraesCS7B02G105100.1	1474	1520
TraesCS7B02G105100.1	256	302
TraesCS7B02G105100.1	1173	1219
TraesCS7B02G105100.1	1905	1951
TraesCS7B02G105100.1	1664	1710
TraesCS7B02G105100.1	343	389
TraesCS7B02G105100.1	1602	1648
TraesCS7B02G105100.1	5	50
TraesCS7B02G105100.1	140	185
TraesCS7B02G105100.1	174	219
TraesCS7B02G105100.1	1090	1135
TraesCS7B02G105100.1	5	50

TraesCS7B02G105100.1	140	185
TraesCS7B02G105100.1	174	219
TraesCS7B02G105100.1	1090	1135
TraesCS7B02G105100.1	1881	1931
TraesCS7B02G105200.1	111	157
TraesCS7B02G105200.1	112	157
TraesCS7B02G105200.1	251	296
TraesCS7B02G105200.1	1905	1950
TraesCS7B02G105200.1	1748	1797
TraesCS7B02G105200.1	1474	1520
TraesCS7B02G105200.1	111	157
TraesCS7B02G105200.1	251	297
TraesCS7B02G105200.1	1173	1219
TraesCS7B02G105200.1	1905	1951
TraesCS7B02G105200.1	111	157
TraesCS7B02G105200.1	1410	1457
TraesCS7B02G105200.1	1016	1062
TraesCS7B02G105200.1	1665	1711
TraesCS7B02G105200.1	1545	1591
TraesCS7B02G105200.1	338	384
TraesCS7B02G105200.1	1603	1649
TraesCS7B02G105200.1	1412	1463
TraesCS7B02G105200.1	0	45
TraesCS7B02G105200.1	135	180
TraesCS7B02G105200.1	169	214
TraesCS7B02G105200.1	451	496
TraesCS7B02G105200.1	474	519
TraesCS7B02G105200.1	901	946
TraesCS7B02G105200.1	1089	1134
TraesCS7B02G105200.1	0	45
TraesCS7B02G105200.1	135	180
TraesCS7B02G105200.1	169	214
TraesCS7B02G105200.1	451	496
TraesCS7B02G105200.1	474	519
TraesCS7B02G105200.1	901	946
TraesCS7B02G105200.1	1089	1134
TraesCS7B02G105200.1	1881	1931
TraesCS7B02G105300.1	498	543
TraesCS7B02G105300.1	685	730
TraesCS7B02G105300.1	1908	1953
TraesCS7B02G105300.1	746	792
TraesCS7B02G105300.1	122	168
TraesCS7B02G105300.1	1679	1725
TraesCS7B02G105300.1	498	544
TraesCS7B02G105300.1	685	731
TraesCS7B02G105300.1	1908	1954
TraesCS7B02G105300.1	163	209
TraesCS7B02G105300.1	1301	1347
TraesCS7B02G105300.1	1666	1712
TraesCS7B02G105300.1	709	758
TraesCS7B02G105300.1	541	589
TraesCS7B02G105300.1	1603	1649
TraesCS7B02G105300.1	1636	1682
TraesCS7B02G105300.1	227	272
TraesCS7B02G105300.1	397	442
TraesCS7B02G105300.1	1492	1537
TraesCS7B02G105300.1	227	272
TraesCS7B02G105300.1	397	442

TraesCS7B02G105300.1	1492	1537
TraesCS7B02G105300.1	232	278
TraesCS7B02G105300.1	1280	1329
TraesCS7B02G105300.1	8	57
TraesCS7B02G105300.1	1285	1334
TraesCS7D02G099600.1	536	581
TraesCS7D02G099600.1	850	897
TraesCS7D02G099600.1	1333	1378
TraesCS7D02G099600.1	1658	1708
TraesCS7D02G099600.1	1660	1705
TraesCS7D02G099600.1	1866	1911
TraesCS7D02G099600.1	1445	1491
TraesCS7D02G099600.1	1240	1286
TraesCS7D02G099600.1	285	332
TraesCS7D02G099600.1	536	582
TraesCS7D02G099600.1	1332	1378
TraesCS7D02G099600.1	1660	1706
TraesCS7D02G099600.1	1866	1912
TraesCS7D02G099600.1	693	742
TraesCS7D02G099600.1	791	840
TraesCS7D02G099600.1	652	698
TraesCS7D02G099600.1	1124	1170
TraesCS7D02G099600.1	1447	1496
TraesCS7D02G099600.1	282	329
TraesCS7D02G099600.1	1349	1397
TraesCS7D02G099600.1	7	52
TraesCS7D02G099600.1	908	953
TraesCS7D02G099600.1	1605	1650
TraesCS7D02G099600.1	1769	1814
TraesCS7D02G099600.1	7	52
TraesCS7D02G099600.1	908	953
TraesCS7D02G099600.1	1605	1650
TraesCS7D02G099600.1	1769	1814
TraesCS7D02G099600.1	1052	1098
TraesCS7D02G099600.1	1946	1995
TraesCS7D02G153900.1	295	340
TraesCS7D02G153900.1	438	483
TraesCS7D02G153900.1	1048	1095
TraesCS7D02G153900.1	1427	1473
TraesCS7D02G153900.1	171	217
TraesCS7D02G153900.1	320	366
TraesCS7D02G153900.1	1357	1403
TraesCS7D02G153900.1	1733	1779
TraesCS7D02G153900.1	294	340
TraesCS7D02G153900.1	438	484
TraesCS7D02G153900.1	522	568
TraesCS7D02G153900.1	769	815
TraesCS7D02G153900.1	57	106
TraesCS7D02G153900.1	85	131
TraesCS7D02G153900.1	-12	36
TraesCS7D02G153900.1	199	248
TraesCS7D02G153900.1	124	170
TraesCS7D02G153900.1	1470	1521
TraesCS7D02G153900.1	1063	1109
TraesCS7D02G153900.1	1084	1130
TraesCS7D02G153900.1	1283	1329
TraesCS7D02G153900.1	1120	1165
TraesCS7D02G153900.1	1120	1165

TraesCS7D02G153900.1	403	449
TraesCS7D02G153900.1	1031	1080
TraesCS7D02G161200.1	1131	1176
TraesCS7D02G161200.1	1341	1386
TraesCS7D02G161200.1	1378	1423
TraesCS7D02G161200.1	1537	1582
TraesCS7D02G161200.1	81	127
TraesCS7D02G161200.1	1464	1510
TraesCS7D02G161200.1	1830	1876
TraesCS7D02G161200.1	1095	1141
TraesCS7D02G161200.1	1536	1582
TraesCS7D02G161200.1	1131	1177
TraesCS7D02G161200.1	1341	1387
TraesCS7D02G161200.1	1378	1424
TraesCS7D02G161200.1	689	737
TraesCS7D02G161200.1	1300	1346
TraesCS7D02G161200.1	1713	1760
TraesCS7D02G161200.1	1271	1320
TraesCS7D02G161200.1	269	315
TraesCS7D02G161200.1	11	56
TraesCS7D02G161200.1	489	534
TraesCS7D02G161200.1	1535	1580
TraesCS7D02G161200.1	1687	1732
TraesCS7D02G161200.1	11	56
TraesCS7D02G161200.1	489	534
TraesCS7D02G161200.1	1535	1580
TraesCS7D02G161200.1	1687	1732
TraesCS7D02G161200.1	509	557
TraesCS7D02G201300.1	572	618
TraesCS7D02G201300.1	573	618
TraesCS7D02G201300.1	1130	1175
TraesCS7D02G201300.1	209	255
TraesCS7D02G201300.1	1676	1722
TraesCS7D02G201300.1	1213	1260
TraesCS7D02G201300.1	878	925
TraesCS7D02G201300.1	332	378
TraesCS7D02G201300.1	572	618
TraesCS7D02G201300.1	572	618
TraesCS7D02G201300.1	1130	1176
TraesCS7D02G201300.1	1520	1569
TraesCS7D02G201300.1	555	601
TraesCS7D02G201300.1	44	93.5
TraesCS7D02G201300.1	62	110
TraesCS7D02G201300.1	1357	1405
TraesCS7D02G201300.1	1601	1647
TraesCS7D02G201300.1	1633	1679
TraesCS7D02G201300.1	740	787
TraesCS7D02G201300.1	741	787
TraesCS7D02G201300.1	1163	1208
TraesCS7D02G201300.1	1163	1208
TraesCS7D02G201300.1	1109	1155
TraesCS7D02G201300.1	699	748
TraesCS7D02G201300.1	1221	1270
TraesCS7D02G201400.1	25	74
TraesCS7D02G201400.1	27	73
TraesCS7D02G201400.1	28	73
TraesCS7D02G201400.1	144	189
TraesCS7D02G201400.1	303	348

TraesCS7D02G201400.1	1038	1083
TraesCS7D02G201400.1	1908	1953
TraesCS7D02G201400.1	1675	1721
TraesCS7D02G201400.1	27	73
TraesCS7D02G201400.1	303	349
TraesCS7D02G201400.1	1908	1954
TraesCS7D02G201400.1	27	73
TraesCS7D02G201400.1	143	189
TraesCS7D02G201400.1	1038	1084
TraesCS7D02G201400.1	705	751
TraesCS7D02G201400.1	1662	1708
TraesCS7D02G201400.1	397	446
TraesCS7D02G201400.1	397	446
TraesCS7D02G201400.1	1249	1298
TraesCS7D02G201400.1	1081	1129
TraesCS7D02G201400.1	1599	1645
TraesCS7D02G201400.1	1632	1678
TraesCS7D02G201400.1	1931	1976
TraesCS7D02G201400.1	1931	1976
TraesCS7D02G201400.1	120	169
TraesCS7D02G201400.1	1884	1933
TraesCS7D02G201400.1	636	685
TraesCSU02G076600.1	483	528
TraesCSU02G076600.1	706	756
TraesCSU02G076600.1	710	755
TraesCSU02G076600.1	780	825
TraesCSU02G076600.1	935	985
TraesCSU02G076600.1	95	142
TraesCSU02G076600.1	576	622
TraesCSU02G076600.1	673	720
TraesCSU02G076600.1	1249	1296
TraesCSU02G076600.1	78	127
TraesCSU02G076600.1	235	284
TraesCSU02G076600.1	831	881
TraesCSU02G076600.1	483	529
TraesCSU02G076600.1	709	755
TraesCSU02G076600.1	779	825
TraesCSU02G076600.1	301	350
TraesCSU02G076600.1	1069	1118
TraesCSU02G076600.1	113	162
TraesCSU02G076600.1	253	299
TraesCSU02G076600.1	370	416
TraesCSU02G076600.1	1562	1610
TraesCSU02G076600.1	419	465
TraesCSU02G076600.1	1750	1796
TraesCSU02G076600.1	877	923
TraesCSU02G076600.1	272	317
TraesCSU02G076600.1	1696	1741
TraesCSU02G076600.1	272	317
TraesCSU02G076600.1	1696	1741
TraesCSU02G076600.1	893	939
TraesCSU02G076600.1	293	342
TraesCSU02G076600.1	528	577
TraesCSU02G076600.1	1709	1758
TraesCSU02G095300.1	1037	1082
TraesCSU02G095300.1	1532	1581
TraesCSU02G095300.1	1651	1701
TraesCSU02G095300.1	1653	1698

TraesCSU02G095300.1	1863	1908
TraesCSU02G095300.1	1423	1469
TraesCSU02G095300.1	1221	1267
TraesCSU02G095300.1	1037	1083
TraesCSU02G095300.1	1653	1699
TraesCSU02G095300.1	1863	1909
TraesCSU02G095300.1	978	1024
TraesCSU02G095300.1	1685	1733
TraesCSU02G095300.1	1322	1369
TraesCSU02G095300.1	1326	1374
TraesCSU02G095300.1	558	604
TraesCSU02G095300.1	-10	35
TraesCSU02G095300.1	1587	1632
TraesCSU02G095300.1	-10	35
TraesCSU02G095300.1	1587	1632
TraesCSU02G095300.1	223	273
TraesCSU02G095300.1	1946	1994
TraesCSU02G202900.1	667	713
TraesCSU02G202900.1	668	713
TraesCSU02G202900.1	678	724
TraesCSU02G202900.1	679	724
TraesCSU02G202900.1	689	735
TraesCSU02G202900.1	690	735
TraesCSU02G202900.1	990	1039
TraesCSU02G202900.1	1051	1100
TraesCSU02G202900.1	1478	1523
TraesCSU02G202900.1	1494	1539
TraesCSU02G202900.1	1029	1075
TraesCSU02G202900.1	404	453
TraesCSU02G202900.1	851	898
TraesCSU02G202900.1	667	713
TraesCSU02G202900.1	678	724
TraesCSU02G202900.1	689	735
TraesCSU02G202900.1	1391	1437
TraesCSU02G202900.1	1475	1523
TraesCSU02G202900.1	1491	1539
TraesCSU02G202900.1	667	713
TraesCSU02G202900.1	678	724
TraesCSU02G202900.1	689	735
TraesCSU02G202900.1	1477	1523
TraesCSU02G202900.1	1493	1539
TraesCSU02G202900.1	1463	1509
TraesCSU02G202900.1	1435	1481
TraesCSU02G202900.1	342	390
TraesCSU02G202900.1	1232	1278
TraesCSU02G202900.1	1263	1309
TraesCSU02G202900.1	1274	1320
TraesCSU02G202900.1	1750	1796
TraesCSU02G202900.1	820	866
TraesCSU02G202900.1	750	795
TraesCSU02G202900.1	967	1012
TraesCSU02G202900.1	1696	1741
TraesCSU02G202900.1	750	795
TraesCSU02G202900.1	967	1012
TraesCSU02G202900.1	1696	1741
TraesCSU02G202900.1	501	547
TraesCSU02G202900.1	1042	1088
TraesCSU02G202900.1	1256	1305

TraesCSU02G202900.1	1709	1758
TraesCSU02G226400.1	1102	1147
TraesCSU02G226400.1	1565	1610
TraesCSU02G226400.1	1457	1503
TraesCSU02G226400.1	1102	1148
TraesCSU02G226400.1	1565	1611
TraesCSU02G226400.1	1534	1580
TraesCSU02G226400.1	1314	1364
TraesCSU02G226400.1	780	832
TraesCSU02G226400.1	1738	1786
TraesCSU02G226400.1	1137	1183
TraesCSU02G226400.1	1750	1796
TraesCSU02G226400.1	1696	1741
TraesCSU02G226400.1	1696	1741
TraesCSU02G226400.1	1412	1458
TraesCSU02G226400.1	99	148
TraesCSU02G226400.1	1709	1758
TraesCSU02G233000.1	113	160
TraesCSU02G233000.1	1572	1618
TraesCSU02G233000.1	16	63
TraesCSU02G233000.1	190	237
TraesCSU02G233000.1	266	315
TraesCSU02G233000.1	1142	1192
TraesCSU02G233000.1	267	317
TraesCSU02G233000.1	1750	1796
TraesCSU02G233000.1	363	413
TraesCSU02G233000.1	682	727
TraesCSU02G233000.1	1696	1741
TraesCSU02G233000.1	682	727
TraesCSU02G233000.1	1696	1741
TraesCSU02G233000.1	1709	1758

Wheat PR1 gene family
Cis-acting element
light responsive
light responsive
defense and stress responsive
light responsive
light responsive
light responsive
light responsive
light responsive
light responsive
MeJA responsive
light responsive
light responsive
abscisic acid responsive
MeJA responsive
defense and stress responsive
abscisic acid responsive
light responsive
light responsive
abscisic acid responsive
MeJA responsive
abscisic acid responsive
zein metabolism regulation
light responsive
gibberellin responsive
defense and stress responsive
defense and stress responsive
MeJA responsive
gibberellin responsive
light responsive
light responsive
MeJA responsive
light responsive
light responsive
gibberellin responsive
zein metabolism regulation
light responsive
light responsive
light responsive
light responsive
light responsive
light responsive
MYBHv1 binding site
light responsive
light responsive
light responsive
abscisic acid responsive
light responsive
MeJA responsive
auxin responsive
light responsive
gibberellin responsive
light responsive
light responsive
abscisic acid responsive
MeJA responsive
defense and stress responsive

abscisic acid responsive
abscisic acid responsive
light responsive
light responsive
abscisic acid responsive
abscisic acid responsive
defense and stress responsive
light responsive
defense and stress responsive
zein metabolism regulation
abscisic acid responsive
MeJA responsive
MYBHv1 binding site
light responsive
auxin responsive
light responsive
light responsive
light responsive
defense and stress responsive
abscisic acid responsive
light responsive
MeJA responsive
abscisic acid responsive
light responsive
abscisic acid responsive
gibberellin responsive
MeJA responsive
light responsive
light responsive
zein metabolism regulation
light responsive
light responsive
light responsive
defense and stress responsive
abscisic acid responsive
light responsive
abscisic acid responsive
auxin responsive
light responsive
light responsive
light responsive
auxin responsive
light responsive
gibberellin responsive
gibberellin responsive
MYBHv1 binding site
light responsive
abscisic acid responsive
AT-rich DNA binding protein (ATBP-1)
light responsive
abscisic acid responsive
light responsive
light responsive
light responsive
light responsive
light responsive
light responsive
abscisic acid responsive

defense and stress responsive
light responsive
defense and stress responsive
light responsive
MeJA responsive
zein metabolism regulation
abscisic acid responsive
MeJA responsive
defense and stress responsive
gibberellin responsive
defense and stress responsive
salicylic acid responsive
defense and stress responsive
light responsive
light responsive
light responsive
MeJA responsive
MeJA responsive
light responsive
abscisic acid responsive
light responsive
light responsive
gibberellin responsive
light responsive
light responsive
MeJA responsive
light responsive
gibberellin responsive
MeJA responsive
zein metabolism regulation
abscisic acid responsive
light responsive
MeJA responsive
light responsive
abscisic acid responsive
light responsive
zein metabolism regulation
MYBHv1 binding site
defense and stress responsive
abscisic acid responsive
MYBHv1 binding site
light responsive
light responsive
zein metabolism regulation
zein metabolism regulation
MeJA responsive
light responsive
MeJA responsive
MeJA responsive
MYBHv1 binding site
light responsive
MeJA responsive
light responsive
light responsive
MeJA responsive
defense and stress responsive
light responsive
light responsive

defense and stress responsive
light responsive
MeJA responsive
light responsive
MeJA responsive
light responsive
light responsive
MeJA responsive
light responsive
abscisic acid responsive
defense and stress responsive
zein metabolism regulation
MeJA responsive
light responsive
abscisic acid responsive
light responsive
light responsive
abscisic acid responsive
salicylic acid responsive
auxin responsive
light responsive
abscisic acid responsive
defense and stress responsive
zein metabolism regulation
MeJA responsive
light responsive
abscisic acid responsive
light responsive
light responsive
abscisic acid responsive
salicylic acid responsive
auxin responsive
zein metabolism regulation
MeJA responsive
MeJA responsive
MeJA responsive
light responsive
abscisic acid responsive
light responsive
light responsive
light responsive
light responsive
light responsive
abscisic acid responsive
light responsive
MYBHv1 binding site
light responsive
light responsive
abscisic acid responsive
abscisic acid responsive
light responsive
light responsive
light responsive
MeJA responsive
light responsive
abscisic acid responsive
light responsive
abscisic acid responsive

abscisic acid responsive
light responsive
light responsive
light responsive
light responsive
AT-rich DNA binding protein (ATBP-1)
light responsive
light responsive
gibberellin responsive
light responsive
light responsive
light responsive
abscisic acid responsive
defense and stress responsive
light responsive
light responsive
abscisic acid responsive
MeJA responsive
light responsive
abscisic acid responsive
MeJA responsive
defense and stress responsive
gibberellin responsive
light responsive
auxin responsive
gibberellin responsive
light responsive
AT-rich DNA binding protein (ATBP-1)
light responsive
light responsive
light responsive
abscisic acid responsive
light responsive
gibberellin responsive
light responsive
abscisic acid responsive
light responsive
gibberellin responsive
abscisic acid responsive
abscisic acid responsive
light responsive
MeJA responsive
light responsive
abscisic acid responsive
MeJA responsive
defense and stress responsive
abscisic acid responsive
abscisic acid responsive
auxin responsive
defense and stress responsive
defense and stress responsive
gibberellin responsive
light responsive
light responsive
light responsive
light responsive
light responsive
light responsive

light responsive
light responsive
light responsive
MeJA responsive
MeJA responsive
MeJA responsive
MeJA responsive
MYBHv1 binding site
MYBHv1 binding site
zein metabolism regulation
abscisic acid responsive
abscisic acid responsive
abscisic acid responsive
abscisic acid responsive
abscisic acid responsive
abscisic acid responsive
abscisic acid responsive
abscisic acid responsive
abscisic acid responsive
abscisic acid responsive
abscisic acid responsive
auxin responsive
defense and stress responsive
light responsive
light responsive
light responsive
light responsive
light responsive
light responsive
light responsive
light responsive
light responsive
light responsive
light responsive
light responsive
light responsive
light responsive
light responsive
light responsive
light responsive
light responsive
light responsive
light responsive
light responsive
MeJA responsive
MeJA responsive
MeJA responsive
MeJA responsive
MeJA responsive
MeJA responsive
MeJA responsive
MeJA responsive
MYBHv1 binding site
MYBHv1 binding site
zein metabolism regulation
abscisic acid responsive
abscisic acid responsive
abscisic acid responsive

abscisic acid responsive
defense and stress responsive
defense and stress responsive
gibberellin responsive
light responsive
light responsive
light responsive
light responsive
light responsive
light responsive
light responsive
light responsive
light responsive
light responsive
light responsive
light responsive
light responsive
MeJA responsive
MeJA responsive
MeJA responsive
MeJA responsive
MeJA responsive
MeJA responsive
MeJA responsive
MeJA responsive
MeJA responsive
MeJA responsive
MeJA responsive
MeJA responsive
MeJA responsive
MeJA responsive
MeJA responsive
MeJA responsive
abscisic acid responsive
abscisic acid responsive
abscisic acid responsive
abscisic acid responsive
defense and stress responsive
gibberellin responsive
light responsive
light responsive
light responsive
light responsive
light responsive
light responsive
light responsive
light responsive
light responsive
light responsive
light responsive
light responsive
light responsive
light responsive
MeJA responsive
MeJA responsive
MeJA responsive
MeJA responsive
MeJA responsive

MeJA responsive
zein metabolism regulation
abscisic acid responsive
abscisic acid responsive
abscisic acid responsive
defense and stress responsive
gibberellin responsive
light responsive
light responsive
light responsive
light responsive
light responsive
light responsive
light responsive
light responsive
light responsive
light responsive
light responsive
MeJA responsive
MeJA responsive
MeJA responsive
MeJA responsive
MeJA responsive
MeJA responsive
MYBHv1 binding site
zein metabolism regulation
zein metabolism regulation
abscisic acid responsive
abscisic acid responsive
abscisic acid responsive
auxin responsive
defense and stress responsive
gibberellin responsive
gibberellin responsive
gibberellin responsive
light responsive
light responsive
light responsive
light responsive
light responsive
light responsive
light responsive
light responsive
light responsive
light responsive
light responsive
MeJA responsive
MeJA responsive
MeJA responsive
MeJA responsive
salicylic acid responsive
abscisic acid responsive
abscisic acid responsive
abscisic acid responsive
abscisic acid responsive
abscisic acid responsive
defense and stress responsive
defense and stress responsive

light responsive
light responsive
light responsive
light responsive
light responsive
light responsive
light responsive
light responsive
light responsive
light responsive
light responsive
light responsive
light responsive
MeJA responsive
MeJA responsive
MeJA responsive
MeJA responsive
MeJA responsive
MeJA responsive
MeJA responsive
MeJA responsive
MYBHv1 binding site
MYBHv1 binding site
salicylic acid responsive
zein metabolism regulation
zein metabolism regulation
abscisic acid responsive
abscisic acid responsive
abscisic acid responsive
abscisic acid responsive
abscisic acid responsive
abscisic acid responsive
defense and stress responsive
gibberellin responsive
gibberellin responsive
light responsive
light responsive
light responsive
light responsive
light responsive
light responsive
light responsive
light responsive
light responsive
light responsive
light responsive
light responsive
light responsive
light responsive
light responsive
light responsive
MeJA responsive
MeJA responsive
MeJA responsive
MeJA responsive
MeJA responsive
MeJA responsive
light responsive

light responsive
MeJA responsive
light responsive
abscisic acid responsive
MYBHv1 binding site
light responsive
abscisic acid responsive
light responsive
MeJA responsive
MeJA responsive
abscisic acid responsive
light responsive
MeJA responsive
abscisic acid responsive
abscisic acid responsive
abscisic acid responsive
MeJA responsive
abscisic acid responsive
auxin responsive
light responsive
light responsive
light responsive
light responsive
light responsive
light responsive
light responsive
light responsive
light responsive
light responsive
light responsive
light responsive
light responsive
light responsive
MeJA responsive
MeJA responsive
MeJA responsive
MeJA responsive
MeJA responsive
MeJA responsive
MeJA responsive
MeJA responsive
MeJA responsive
MeJA responsive
defense and stress responsive
defense and stress responsive
MYBHv1 binding site
light responsive
light responsive
MeJA responsive
light responsive
light responsive
light responsive
abscisic acid responsive
abscisic acid responsive
salicylic acid responsive
abscisic acid responsive
auxin responsive
auxin responsive

defense and stress responsive
light responsive
light responsive
light responsive
light responsive
light responsive
light responsive
light responsive
light responsive
MeJA responsive
MeJA responsive
MeJA responsive
MeJA responsive
MYBHv1 binding site
abscisic acid responsive
abscisic acid responsive
abscisic acid responsive
gibberellin responsive
light responsive
light responsive
light responsive
MeJA responsive
MeJA responsive
MeJA responsive
MeJA responsive
salicylic acid responsive
zein metabolism regulation
abscisic acid responsive
abscisic acid responsive
abscisic acid responsive
abscisic acid responsive
abscisic acid responsive
auxin responsive
auxin responsive
light responsive
light responsive
light responsive
light responsive
light responsive
light responsive
light responsive
light responsive
MeJA responsive
MeJA responsive
MeJA responsive
MeJA responsive
MeJA responsive
MeJA responsive
MeJA responsive
MeJA responsive
MeJA responsive
MeJA responsive
MeJA responsive
MeJA responsive
abscisic acid responsive
abscisic acid responsive
abscisic acid responsive
abscisic acid responsive

abscisic acid responsive
gibberellin responsive
light responsive
light responsive
light responsive
light responsive
light responsive
light responsive
MeJA responsive
MeJA responsive
MeJA responsive
MeJA responsive
MYBHv1 binding site
light responsive
light responsive
abscisic acid responsive
defense and stress responsive
MeJA responsive
abscisic acid responsive
MeJA responsive
abscisic acid responsive
gibberellin responsive
AT-rich DNA binding protein (ATBP-1)
abscisic acid responsive
light responsive
light responsive
abscisic acid responsive
light responsive
light responsive
light responsive
light responsive
light responsive
light responsive
light responsive
light responsive
MeJA responsive
MeJA responsive
zein metabolism regulation
zein metabolism regulation
abscisic acid responsive
defense and stress responsive
defense and stress responsive
defense and stress responsive
defense and stress responsive
defense and stress responsive
gibberellin responsive
light responsive
light responsive
light responsive
light responsive
light responsive
light responsive
light responsive
MeJA responsive
MeJA responsive
MeJA responsive
MeJA responsive
MeJA responsive

MeJA responsive
MeJA responsive
MeJA responsive
zein metabolism regulation
zein metabolism regulation
zein metabolism regulation
abscisic acid responsive
auxin responsive
auxin responsive
auxin responsive
defense and stress responsive
defense and stress responsive
light responsive
light responsive
light responsive
light responsive
light responsive
light responsive
MeJA responsive
MeJA responsive
MeJA responsive
MeJA responsive
MeJA responsive
MeJA responsive
MeJA responsive
MeJA responsive
MeJA responsive
MeJA responsive
MYBHv1 binding site
salicylic acid responsive
zein metabolism regulation
abscisic acid responsive
auxin responsive
light responsive
light responsive
light responsive
light responsive
light responsive
light responsive
light responsive
light responsive
MeJA responsive
MeJA responsive
MeJA responsive
MeJA responsive
MeJA responsive
MeJA responsive
MeJA responsive
MeJA responsive
MeJA responsive
MeJA responsive
MeJA responsive
MeJA responsive
MeJA responsive
MeJA responsive
zein metabolism regulation
zein metabolism regulation
abscisic acid responsive

abscisic acid responsive
abscisic acid responsive
abscisic acid responsive
abscisic acid responsive
abscisic acid responsive
abscisic acid responsive
abscisic acid responsive
abscisic acid responsive
auxin responsive
auxin responsive
defense and stress responsive
gibberellin responsive
light responsive
light responsive
light responsive
light responsive
light responsive
light responsive
light responsive
light responsive
light responsive
light responsive
light responsive
light responsive
light responsive
light responsive
light responsive
light responsive
MeJA responsive
MeJA responsive
MeJA responsive
MeJA responsive
MeJA responsive
MeJA responsive
MYBHv1 binding site
salicylic acid responsive
zein metabolism regulation
abscisic acid responsive
abscisic acid responsive
abscisic acid responsive
auxin responsive
auxin responsive
light responsive
light responsive
light responsive
light responsive
MeJA responsive
MeJA responsive
MeJA responsive
MeJA responsive
MeJA responsive
MeJA responsive
MeJA responsive
MeJA responsive
MeJA responsive
MeJA responsive
MeJA responsive
MeJA responsive

MeJA responsive
MeJA responsive
MYBHv1 binding site
light responsive
zein metabolism regulation
light responsive
light responsive
light responsive
zein metabolism regulation
MeJA responsive
abscisic acid responsive
gibberellin responsive
AT-rich DNA binding protein (ATBP-1)
abscisic acid responsive
abscisic acid responsive
MeJA responsive
abscisic acid responsive
light responsive
abscisic acid responsive
abscisic acid responsive
light responsive
light responsive
light responsive
light responsive
light responsive
MeJA responsive
MeJA responsive
zein metabolism regulation
zein metabolism regulation
zein metabolism regulation
abscisic acid responsive
abscisic acid responsive
defense and stress responsive
defense and stress responsive
defense and stress responsive
gibberellin responsive
light responsive
light responsive
light responsive
light responsive
light responsive
light responsive
light responsive
light responsive
MeJA responsive
MeJA responsive
MeJA responsive
MeJA responsive
MeJA responsive
MeJA responsive
MeJA responsive
MeJA responsive
zein metabolism regulation
abscisic acid responsive
defense and stress responsive
gibberellin responsive
light responsive
light responsive

light responsive
light responsive
light responsive
light responsive
light responsive
light responsive
light responsive
light responsive
light responsive
MeJA responsive
MeJA responsive
MeJA responsive
MeJA responsive
MeJA responsive
MeJA responsive
MeJA responsive
MeJA responsive
MeJA responsive
MeJA responsive
MeJA responsive
MeJA responsive
MeJA responsive
MeJA responsive
MeJA responsive
MeJA responsive
MYBHv1 binding site
zein metabolism regulation
abscisic acid responsive
abscisic acid responsive
abscisic acid responsive
abscisic acid responsive
abscisic acid responsive
abscisic acid responsive
defense and stress responsive
light responsive
light responsive
light responsive
light responsive
light responsive
light responsive
light responsive
light responsive
light responsive
light responsive
light responsive
MeJA responsive
MeJA responsive
MeJA responsive
MeJA responsive
MeJA responsive
MeJA responsive
MeJA responsive
MeJA responsive
MeJA responsive
MeJA responsive
MeJA responsive
MeJA responsive
MeJA responsive

MeJA responsive
MYBHv1 binding site
abscisic acid responsive
abscisic acid responsive
abscisic acid responsive
abscisic acid responsive
abscisic acid responsive
abscisic acid responsive
abscisic acid responsive
auxin responsive
defense and stress responsive
defense and stress responsive
defense and stress responsive
defense and stress responsive
light responsive
light responsive
light responsive
light responsive
light responsive
light responsive
light responsive
light responsive
light responsive
light responsive
MeJA responsive
MeJA responsive
MeJA responsive
MeJA responsive
MYBHv1 binding site
salicylic acid responsive
abscisic acid responsive
abscisic acid responsive
abscisic acid responsive
abscisic acid responsive
abscisic acid responsive
abscisic acid responsive
abscisic acid responsive
abscisic acid responsive
abscisic acid responsive
abscisic acid responsive
abscisic acid responsive
abscisic acid responsive
auxin responsive
defense and stress responsive
gibberellin responsive
light responsive
light responsive
light responsive
light responsive
light responsive
light responsive
light responsive
light responsive
light responsive
light responsive
light responsive
light responsive
light responsive

light responsive
light responsive
light responsive
light responsive
light responsive
light responsive
light responsive
light responsive
light responsive
light responsive
light responsive
MYBHv1 binding site
abscisic acid responsive
abscisic acid responsive
abscisic acid responsive
abscisic acid responsive
abscisic acid responsive
abscisic acid responsive
abscisic acid responsive
abscisic acid responsive
abscisic acid responsive
abscisic acid responsive
abscisic acid responsive
auxin responsive
defense and stress responsive
light responsive
light responsive
light responsive
light responsive
light responsive
light responsive
light responsive
light responsive
light responsive
light responsive
light responsive
light responsive
light responsive
light responsive
light responsive
light responsive
light responsive
light responsive
light responsive
MeJA responsive
MeJA responsive
abscisic acid responsive
abscisic acid responsive
abscisic acid responsive
abscisic acid responsive
abscisic acid responsive
abscisic acid responsive
abscisic acid responsive
abscisic acid responsive
abscisic acid responsive
abscisic acid responsive
abscisic acid responsive
auxin responsive

defense and stress responsive
light responsive
light responsive
light responsive
light responsive
light responsive
light responsive
light responsive
light responsive
light responsive
light responsive
light responsive
light responsive
light responsive
light responsive
light responsive
light responsive
light responsive
MeJA responsive
MeJA responsive
zein metabolism regulation
abscisic acid responsive
abscisic acid responsive
abscisic acid responsive
abscisic acid responsive
gibberellin responsive
gibberellin responsive
light responsive
light responsive
light responsive
light responsive
light responsive
light responsive
light responsive
light responsive
light responsive
light responsive
light responsive
MeJA responsive
MeJA responsive
MYBHv1 binding site
abscisic acid responsive
abscisic acid responsive
abscisic acid responsive
abscisic acid responsive
gibberellin responsive
gibberellin responsive
light responsive
light responsive
light responsive
light responsive
light responsive
light responsive
light responsive
light responsive
light responsive
MeJA responsive
MeJA responsive

salicylic acid responsive
abscisic acid responsive
abscisic acid responsive
abscisic acid responsive
AT-rich DNA binding protein (ATBP-1)
gibberellin responsive
gibberellin responsive
light responsive
light responsive
light responsive
light responsive
light responsive
light responsive
light responsive
light responsive
MeJA responsive
MeJA responsive
MYBHv1 binding site
salicylic acid responsive
salicylic acid responsive
abscisic acid responsive
abscisic acid responsive
abscisic acid responsive
gibberellin responsive
gibberellin responsive
light responsive
light responsive
light responsive
light responsive
light responsive
light responsive
light responsive
light responsive
MeJA responsive
MeJA responsive
MYBHv1 binding site
salicylic acid responsive
salicylic acid responsive
abscisic acid responsive
abscisic acid responsive
abscisic acid responsive
abscisic acid responsive
gibberellin responsive
gibberellin responsive
gibberellin responsive
light responsive
light responsive
light responsive
light responsive
light responsive
light responsive
light responsive
light responsive
light responsive
MeJA responsive
MeJA responsive
salicylic acid responsive
abscisic acid responsive

abscisic acid responsive
abscisic acid responsive
gibberellin responsive
gibberellin responsive
light responsive
light responsive
light responsive
light responsive
light responsive
light responsive
light responsive
light responsive
MeJA responsive
MeJA responsive
MYBHv1 binding site
salicylic acid responsive
salicylic acid responsive
abscisic acid responsive
abscisic acid responsive
abscisic acid responsive
abscisic acid responsive
gibberellin responsive
gibberellin responsive
light responsive
light responsive
light responsive
light responsive
light responsive
light responsive
light responsive
light responsive
light responsive
MeJA responsive
MeJA responsive
MYBHv1 binding site
salicylic acid responsive
abscisic acid responsive
abscisic acid responsive
defense and stress responsive
defense and stress responsive
defense and stress responsive
defense and stress responsive
light responsive
light responsive
light responsive
light responsive
light responsive
light responsive
light responsive
light responsive
light responsive
light responsive
MeJA responsive
MeJA responsive
MeJA responsive
MeJA responsive
MeJA responsive
MeJA responsive

MeJA responsive
MeJA responsive
MeJA responsive
MeJA responsive
zein metabolism regulation
abscisic acid responsive
abscisic acid responsive
abscisic acid responsive
light responsive
light responsive
light responsive
light responsive
light responsive
light responsive
light responsive
light responsive
MeJA responsive
MeJA responsive
MeJA responsive
MeJA responsive
MeJA responsive
MeJA responsive
salicylic acid responsive
zein metabolism regulation
zein metabolism regulation
abscisic acid responsive
abscisic acid responsive
abscisic acid responsive
abscisic acid responsive
abscisic acid responsive
abscisic acid responsive
auxin responsive
auxin responsive
defense and stress responsive
light responsive
light responsive
light responsive
light responsive
light responsive
light responsive
light responsive
light responsive
light responsive
light responsive
light responsive
light responsive
MeJA responsive
MeJA responsive
MeJA responsive
MeJA responsive
MYBHv1 binding site
MYBHv1 binding site
salicylic acid responsive
zein metabolism regulation
zein metabolism regulation
abscisic acid responsive
abscisic acid responsive
auxin responsive

auxin responsive
auxin responsive
defense and stress responsive
defense and stress responsive
gibberellin responsive
gibberellin responsive
light responsive
light responsive
light responsive
light responsive
light responsive
light responsive
light responsive
light responsive
light responsive
light responsive
light responsive
light responsive
light responsive
light responsive
light responsive
light responsive
light responsive
MeJA responsive
MeJA responsive
MeJA responsive
MeJA responsive
MeJA responsive
MeJA responsive
salicylic acid responsive
salicylic acid responsive
salicylic acid responsive
abscisic acid responsive
abscisic acid responsive
abscisic acid responsive
abscisic acid responsive
gibberellin responsive
gibberellin responsive
light responsive
light responsive
light responsive
light responsive
light responsive
light responsive
light responsive
light responsive
light responsive
light responsive
light responsive
light responsive
MeJA responsive
MeJA responsive
MeJA responsive
MeJA responsive
MeJA responsive
MeJA responsive
MeJA responsive
MeJA responsive

MeJA responsive
MeJA responsive
abscisic acid responsive
abscisic acid responsive
abscisic acid responsive
abscisic acid responsive
auxin responsive
auxin responsive
defense and stress responsive
gibberellin responsive
light responsive
light responsive
light responsive
light responsive
light responsive
light responsive
light responsive
light responsive
light responsive
light responsive
light responsive
light responsive
MeJA responsive
MeJA responsive
MeJA responsive
MeJA responsive
zein metabolism regulation
abscisic acid responsive
abscisic acid responsive
abscisic acid responsive
abscisic acid responsive
abscisic acid responsive
abscisic acid responsive
abscisic acid responsive
abscisic acid responsive
auxin responsive
auxin responsive
gibberellin responsive
light responsive
light responsive
light responsive
light responsive
light responsive
light responsive
light responsive
light responsive
light responsive
light responsive
light responsive
MeJA responsive
MeJA responsive
MeJA responsive
MeJA responsive
MeJA responsive
MeJA responsive
MYBHv1 binding site
zein metabolism regulation
abscisic acid responsive

abscisic acid responsive
abscisic acid responsive
abscisic acid responsive
auxin responsive
auxin responsive
auxin responsive
auxin responsive
auxin responsive
defense and stress responsive
light responsive
light responsive
light responsive
light responsive
light responsive
light responsive
light responsive
light responsive
light responsive
light responsive
light responsive
MeJA responsive
MeJA responsive
MeJA responsive
MeJA responsive
MYBHv1 binding site
zein metabolism regulation
auxin responsive
auxin responsive
defense and stress responsive
defense and stress responsive
gibberellin responsive
light responsive
light responsive
light responsive
light responsive
light responsive
MeJA responsive
MeJA responsive
MeJA responsive
MeJA responsive
MeJA responsive
MeJA responsive
abscisic acid responsive
abscisic acid responsive
abscisic acid responsive
abscisic acid responsive
auxin responsive
auxin responsive
auxin responsive
defense and stress responsive
defense and stress responsive
defense and stress responsive
gibberellin responsive
light responsive
light responsive
light responsive
light responsive
light responsive

light responsive
light responsive
light responsive
light responsive
light responsive
light responsive
MeJA responsive
MeJA responsive
MeJA responsive
MeJA responsive
MeJA responsive
MeJA responsive
zein metabolism regulation
zein metabolism regulation
abscisic acid responsive
abscisic acid responsive
abscisic acid responsive
abscisic acid responsive
abscisic acid responsive
abscisic acid responsive
auxin responsive
gibberellin responsive
light responsive
light responsive
light responsive
light responsive
light responsive
light responsive
light responsive
light responsive
light responsive
light responsive
light responsive
light responsive
MeJA responsive
MeJA responsive
MeJA responsive
MeJA responsive
MeJA responsive
MeJA responsive
MeJA responsive
MeJA responsive
MYBHv1 binding site
salicylic acid responsive
zein metabolism regulation
zein metabolism regulation
abscisic acid responsive
abscisic acid responsive
abscisic acid responsive
abscisic acid responsive
auxin responsive
defense and stress responsive
light responsive
light responsive
light responsive
light responsive
light responsive
light responsive

MeJA responsive
MeJA responsive
MeJA responsive
MeJA responsive
MYBHv1 binding site
abscisic acid responsive
abscisic acid responsive
abscisic acid responsive
abscisic acid responsive
abscisic acid responsive
abscisic acid responsive
abscisic acid responsive
auxin responsive
defense and stress responsive
defense and stress responsive
defense and stress responsive
defense and stress responsive
defense and stress responsive
defense and stress responsive
defense and stress responsive
defense and stress responsive
gibberellin responsive
light responsive
light responsive
light responsive
light responsive
light responsive
light responsive
light responsive
light responsive
light responsive
light responsive
light responsive
MeJA responsive
MeJA responsive
MeJA responsive
MeJA responsive
zein metabolism regulation
zein metabolism regulation
abscisic acid responsive
abscisic acid responsive
abscisic acid responsive
abscisic acid responsive
abscisic acid responsive
abscisic acid responsive
auxin responsive
auxin responsive
defense and stress responsive
defense and stress responsive
gibberellin responsive
light responsive
light responsive
light responsive
light responsive
light responsive
light responsive
light responsive
light responsive

light responsive
light responsive
light responsive
light responsive
light responsive
light responsive
MeJA responsive
MeJA responsive
MeJA responsive
MeJA responsive
MeJA responsive
MeJA responsive
MeJA responsive
MeJA responsive
MYBHv1 binding site
zein metabolism regulation
zein metabolism regulation
abscisic acid responsive
abscisic acid responsive
abscisic acid responsive
abscisic acid responsive
abscisic acid responsive
abscisic acid responsive
abscisic acid responsive
auxin responsive
defense and stress responsive
defense and stress responsive
defense and stress responsive
light responsive
light responsive
light responsive
light responsive
light responsive
light responsive
light responsive
light responsive
light responsive
light responsive
MeJA responsive
MeJA responsive
MeJA responsive
MeJA responsive
MeJA responsive
MeJA responsive
MeJA responsive
MeJA responsive
abscisic acid responsive
abscisic acid responsive
abscisic acid responsive
abscisic acid responsive
abscisic acid responsive
abscisic acid responsive
auxin responsive
defense and stress responsive
light responsive
light responsive
light responsive
light responsive

light responsive
light responsive
light responsive
light responsive
light responsive
light responsive
MeJA responsive
MeJA responsive
MeJA responsive
MeJA responsive
abscisic acid responsive
abscisic acid responsive
abscisic acid responsive
abscisic acid responsive
abscisic acid responsive
auxin responsive
defense and stress responsive
defense and stress responsive
defense and stress responsive
light responsive
light responsive
light responsive
light responsive
light responsive
light responsive
light responsive
light responsive
light responsive
light responsive
light responsive
MeJA responsive
MeJA responsive
abscisic acid responsive
defense and stress responsive
defense and stress responsive
light responsive
light responsive
light responsive
light responsive
light responsive
light responsive
salicylic acid responsive
defense and stress responsive
light responsive
light responsive
light responsive
light responsive
MYBHv1 binding site
light responsive
light responsive
MYBHv1 binding site
zein metabolism regulation
abscisic acid responsive
abscisic acid responsive
abscisic acid responsive
abscisic acid responsive
abscisic acid responsive
defense and stress responsive

defense and stress responsive
gibberellin responsive
light responsive
light responsive
light responsive
light responsive
light responsive
light responsive
light responsive
light responsive
light responsive
light responsive
light responsive
light responsive
light responsive
light responsive
light responsive
light responsive
MeJA responsive
MeJA responsive
salicylic acid responsive
zein metabolism regulation
abscisic acid responsive
abscisic acid responsive
abscisic acid responsive
defense and stress responsive
light responsive
light responsive
light responsive
light responsive
light responsive
light responsive
light responsive
light responsive
light responsive
light responsive
light responsive
MeJA responsive
MeJA responsive
MeJA responsive
MeJA responsive
MYBHv1 binding site
salicylic acid responsive
abscisic acid responsive
abscisic acid responsive
defense and stress responsive
defense and stress responsive
light responsive
light responsive
light responsive
light responsive
light responsive
light responsive
MeJA responsive
MeJA responsive
MeJA responsive
MeJA responsive
MeJA responsive

MeJA responsive
MeJA responsive
MeJA responsive
salicylic acid responsive
abscisic acid responsive
abscisic acid responsive
abscisic acid responsive
abscisic acid responsive
defense and stress responsive
defense and stress responsive
light responsive
light responsive
light responsive
light responsive
light responsive
light responsive
light responsive
light responsive
light responsive
light responsive
light responsive
light responsive
MeJA responsive
MeJA responsive
MeJA responsive
MeJA responsive
MeJA responsive
MeJA responsive
MeJA responsive
MeJA responsive
MeJA responsive
MeJA responsive
MeJA responsive
MeJA responsive
MeJA responsive
MeJA responsive
salicylic acid responsive
abscisic acid responsive
abscisic acid responsive
abscisic acid responsive
auxin responsive
defense and stress responsive
defense and stress responsive
light responsive
light responsive
light responsive
light responsive
light responsive
light responsive
light responsive
light responsive
light responsive
light responsive
MeJA responsive
MeJA responsive
MeJA responsive
MeJA responsive
MeJA responsive

MeJA responsive
MYBHv1 binding site
salicylic acid responsive
zein metabolism regulation
zein metabolism regulation
abscisic acid responsive
abscisic acid responsive
abscisic acid responsive
abscisic acid responsive
abscisic acid responsive
abscisic acid responsive
auxin responsive
defense and stress responsive
gibberellin responsive
light responsive
light responsive
light responsive
light responsive
light responsive
light responsive
light responsive
light responsive
light responsive
light responsive
light responsive
MeJA responsive
MeJA responsive
MeJA responsive
MeJA responsive
MeJA responsive
MeJA responsive
MeJA responsive
MeJA responsive
MYBHv1 binding site
zein metabolism regulation
abscisic acid responsive
abscisic acid responsive
abscisic acid responsive
auxin responsive
defense and stress responsive
defense and stress responsive
defense and stress responsive
defense and stress responsive
light responsive
light responsive
light responsive
light responsive
light responsive
light responsive
light responsive
light responsive
light responsive
light responsive
light responsive
light responsive
light responsive
MeJA responsive
MeJA responsive

MYBHv1 binding site
zein metabolism regulation
abscisic acid responsive
abscisic acid responsive
abscisic acid responsive
abscisic acid responsive
defense and stress responsive
defense and stress responsive
defense and stress responsive
light responsive
light responsive
light responsive
light responsive
light responsive
light responsive
light responsive
light responsive
light responsive
light responsive
MeJA responsive
MeJA responsive
MeJA responsive
MeJA responsive
MeJA responsive
MeJA responsive
MeJA responsive
MeJA responsive
zein metabolism regulation
abscisic acid responsive
abscisic acid responsive
abscisic acid responsive
defense and stress responsive
defense and stress responsive
gibberellin responsive
gibberellin responsive
light responsive
light responsive
light responsive
light responsive
light responsive
light responsive
light responsive
light responsive
light responsive
light responsive
light responsive
light responsive
light responsive
MeJA responsive
MeJA responsive
MYBHv1 binding site
zein metabolism regulation
zein metabolism regulation
abscisic acid responsive
abscisic acid responsive
abscisic acid responsive
abscisic acid responsive
abscisic acid responsive

abscisic acid responsive
abscisic acid responsive
defense and stress responsive
light responsive
light responsive
light responsive
light responsive
light responsive
light responsive
light responsive
light responsive
light responsive
light responsive
light responsive
light responsive
light responsive
light responsive
MeJA responsive
MeJA responsive
salicylic acid responsive
salicylic acid responsive
zein metabolism regulation
abscisic acid responsive
abscisic acid responsive
abscisic acid responsive
abscisic acid responsive
AT-rich DNA binding protein (ATBP-1)
auxin responsive
defense and stress responsive
gibberellin responsive
gibberellin responsive
light responsive
light responsive
light responsive
light responsive
light responsive
light responsive
light responsive
light responsive
light responsive
light responsive
light responsive
light responsive
light responsive
light responsive
light responsive
MeJA responsive
MeJA responsive
MeJA responsive
MeJA responsive
MYBHv1 binding site
zein metabolism regulation
zein metabolism regulation
zein metabolism regulation
abscisic acid responsive
abscisic acid responsive
abscisic acid responsive
abscisic acid responsive

abscisic acid responsive
auxin responsive
defense and stress responsive
light responsive
light responsive
light responsive
light responsive
light responsive
light responsive
light responsive
light responsive
MeJA responsive
MeJA responsive
MeJA responsive
MeJA responsive
zein metabolism regulation
zein metabolism regulation
abscisic acid responsive
abscisic acid responsive
abscisic acid responsive
abscisic acid responsive
abscisic acid responsive
abscisic acid responsive
abscisic acid responsive
abscisic acid responsive
abscisic acid responsive
abscisic acid responsive
auxin responsive
defense and stress responsive
gibberellin responsive
light responsive
light responsive
light responsive
light responsive
light responsive
light responsive
light responsive
light responsive
light responsive
light responsive
light responsive
light responsive
light responsive
light responsive
light responsive
light responsive
light responsive
light responsive
light responsive
MeJA responsive
MeJA responsive
MeJA responsive
MeJA responsive
MeJA responsive
MeJA responsive
MYBHv1 binding site
MYBHv1 binding site
zein metabolism regulation

zein metabolism regulation
abscisic acid responsive
abscisic acid responsive
auxin responsive
light responsive
light responsive
light responsive
light responsive
light responsive
light responsive
light responsive
light responsive
MeJA responsive
MeJA responsive
MYBHv1 binding site
zein metabolism regulation
zein metabolism regulation
abscisic acid responsive
auxin responsive
gibberellin responsive
light responsive
light responsive
light responsive
light responsive
light responsive
light responsive
MeJA responsive
MeJA responsive
MeJA responsive
MeJA responsive
zein metabolism regulation
