# Supplementary material for: Characterization and functional analyses of wheat TaPR1 genes in response to stripe rust fungal infection
Source: Sci Rep. 2023 Feb 27;13:3362. doi: 10.1038/s41598-023-30456-8 (PMC9971213; doi:10.1038/s41598-023-30456-8)
Supplement: Supplementary file 5 — Supplementary Information 5. [file 41598_2023_30456_MOESM5_ESM.pdf]

Additional file 5. Prediction results of secondary structure of PR1 protein in wheat

Gene-ID	Hh (Alpha helix)	Ee (Extended strand)	Tt (Beta turn)
TraesCS1A02G443800.1	21.09	11.9	6.46
TraesCS1A02G444000.1	38.11	9.02	6.15
TraesCS1B02G478300.1	25.69	12.84	5.05
TraesCS1B02G478500.1	36.73	10.61	5.31
TraesCS1D02G452000.1	33.05	12.24	4.49
TraesCS2A02G439600.1	29.3	12.1	3.18
TraesCS2A02G439700.1	33.15	11.05	7.73
TraesCS2A02G441400.1	25.14	18.03	7.1
TraesCS2B02G403600.1	36.65	13.09	2.09
TraesCS2B02G403700.1	28.41	14.77	6.82
TraesCS2B02G459500.1	31.28	13.41	3.35
TraesCS2B02G459600.1	33.15	14.36	8.29
TraesCS2B02G459700.1	27.87	17.49	7.1
TraesCS2D02G382900.1	28.35	17.01	4.12
TraesCS2D02G436900.1	28.3	15.72	7.55
TraesCS2D02G437000.1	30.05	14.75	3.83
TraesCS2D02G437100.1	28.42	14.21	4.37
TraesCS2D02G437200.1	37.02	12.15	7.73
TraesCS2D02G437300.1	23.76	19.34	4.97
TraesCS2D02G437400.1	26.49	12.97	5.41
TraesCS3A02G477300.1	31.59	19.95	8.97
TraesCS3A02G525700.1	35.15	15.76	4.24
TraesCS3D02G472000.1	31.1	20.38	8.85
TraesCS3D02G530800.1	34.55	16.97	6.06
TraesCS4A02G251300.1	35.68	15.14	3.24
TraesCS4B02G063600.1	37.84	15.68	4.86
TraesCS4D02G062500.1	38.8	14.21	4.92
TraesCS5A02G012900.1	21.15	11.22	8.01
TraesCS5A02G059000.1	33.54	16.46	6.71
TraesCS5A02G183300.1	34.76	16.46	3.66
TraesCS5A02G439700.1	34.34	16.27	3.01
TraesCS5A02G439800.1	35.93	16.17	5.39
TraesCS5A02G439900.1	37.95	15.06	5.42
TraesCS5A02G440000.1	37.35	16.87	3.01
TraesCS5B02G011200.1	24.01	10.86	6.25
TraesCS5B02G066300.1	37.2	16.46	4.27
TraesCS5B02G181500.1	34.15	15.24	3.66
TraesCS5B02G442600.1	37.95	16.87	4.22
TraesCS5B02G442700.1	40	14.59	4.86
TraesCS5B02G442800.1	33.33	15.15	3.64
TraesCS5B02G442900.1	33.33	16.07	2.98
TraesCS5B02G443000.1	35.44	17.72	4.43
TraesCS5B02G443100.1	38.69	11.9	5.95
TraesCS5B02G443200.1	38.79	13.94	3.03
TraesCS5B02G443300.1	38.79	15.15	3.64
TraesCS5B02G443400.1	36.97	17.58	6.06
TraesCS5B02G443500.1	38.79	15.15	3.64
TraesCS5B02G443600.1	40.96	19.15	4.26
TraesCS5B02G443700.1	36.97	17.58	6.06
TraesCS5B02G443800.1	39.39	15.15	6.06
TraesCS5D02G446800.1	34.94	16.87	6.02
TraesCS5D02G446900.1	34.73	16.17	4.19
TraesCS5D02G447000.1	35.71	13.69	6.55
TraesCS5D02G447100.1	37.5	16.67	3.57
TraesCS6A02G345000.1	37.5	15.22	10.33
TraesCS6A02G345100.1	33.91	18.97	11.49

TraesCS6A02G345200.1	38.97	15.49	6.1
TraesCS6A02G346300.1	37.99	14.53	6.15
TraesCS6B02G377700.1	35.11	13.3	7.98
TraesCS6B02G377800.1	32.2	18.08	6.78
TraesCS6B02G378000.1	37.09	16.43	5.63
TraesCS6B02G379800.1	37.43	16.2	5.59
TraesCS6D02G327500.1	35.64	13.83	6.38
TraesCS6D02G327600.1	33.33	16.95	3.95
TraesCS6D02G327700.1	39.91	10.33	4.23
TraesCS6D02G329200.1	38.55	15.64	5.03
TraesCS7A02G152200.1	40.23	10.92	7.47
TraesCS7A02G198800.1	36.63	19.19	5.81
TraesCS7A02G198900.1	33.91	16.09	6.32
TraesCS7A02G565100.1	32.93	14.97	7.19
TraesCS7B02G056100.1	41.86	11.05	8.14
TraesCS7B02G104900.1	32.18	16.67	6.32
TraesCS7B02G105000.1	42.94	10.43	3.07
TraesCS7B02G105100.1	31.61	17.24	4.6
TraesCS7B02G105200.1	35.63	16.67	2.87
TraesCS7B02G105300.1	34.48	18.97	3.45
TraesCS7D02G099600.1	36.09	15.98	3.55
TraesCS7D02G153900.1	44.77	12.21	5.81
TraesCS7D02G161200.1	35.98	15.85	4.27
TraesCS7D02G201300.1	39.53	14.53	4.65
TraesCS7D02G201400.1	38.73	15.61	5.2
TraesCSU02G076600.1	33.33	18.45	4.17
TraesCSU02G095300.1	32.94	17.65	4.12
TraesCSU02G202900.1	33.33	18.45	4.17
TraesCSU02G226400.1	37.5	13.1	5.95
TraesCSU02G233000.1	33.33	18.45	4.17

t genome.

Gene-ID	Cc (Random coil)
TraesCS1A02G443800.1	60.54
TraesCS1A02G444000.1	46.72
TraesCS1B02G478300.1	56.42
TraesCS1B02G478500.1	47.35
TraesCS1D02G452000.1	50.2
TraesCS2A02G439600.1	55.41
TraesCS2A02G439700.1	48.07
TraesCS2A02G441400.1	49.73
TraesCS2B02G403600.1	48.17
TraesCS2B02G403700.1	50
TraesCS2B02G459500.1	51.96
TraesCS2B02G459600.1	44.2
TraesCS2B02G459700.1	47.54
TraesCS2D02G382900.1	50.52
TraesCS2D02G436900.1	48.43
TraesCS2D02G437000.1	51.37
TraesCS2D02G437100.1	53.01
TraesCS2D02G437200.1	43.09
TraesCS2D02G437300.1	51.93
TraesCS2D02G437400.1	55.14
TraesCS3A02G477300.1	39.49
TraesCS3A02G525700.1	44.85
TraesCS3D02G472000.1	39.68
TraesCS3D02G530800.1	42.42
TraesCS4A02G251300.1	45.95
TraesCS4B02G063600.1	41.62
TraesCS4D02G062500.1	42.08
TraesCS5A02G012900.1	59.62
TraesCS5A02G059000.1	43.29
TraesCS5A02G183300.1	45.12
TraesCS5A02G439700.1	46.39
TraesCS5A02G439800.1	42.51
TraesCS5A02G439900.1	41.57
TraesCS5A02G440000.1	42.77
TraesCS5B02G011200.1	58.88
TraesCS5B02G066300.1	42.07
TraesCS5B02G181500.1	46.95
TraesCS5B02G442600.1	40.96
TraesCS5B02G442700.1	40.54
TraesCS5B02G442800.1	47.88
TraesCS5B02G442900.1	47.62
TraesCS5B02G443000.1	42.41
TraesCS5B02G443100.1	43.45
TraesCS5B02G443200.1	44.24
TraesCS5B02G443300.1	42.42
TraesCS5B02G443400.1	39.39
TraesCS5B02G443500.1	42.42
TraesCS5B02G443600.1	35.64
TraesCS5B02G443700.1	39.39
TraesCS5B02G443800.1	39.39
TraesCS5D02G446800.1	42.17
TraesCS5D02G446900.1	44.91
TraesCS5D02G447000.1	44.05
TraesCS5D02G447100.1	42.26
TraesCS6A02G345000.1	36.96
TraesCS6A02G345100.1	35.63

TraesCS6A02G345200.1	39.44
TraesCS6A02G346300.1	41.34
TraesCS6B02G377700.1	43.62
TraesCS6B02G377800.1	42.94
TraesCS6B02G378000.1	40.85
TraesCS6B02G379800.1	40.78
TraesCS6D02G327500.1	44.15
TraesCS6D02G327600.1	45.76
TraesCS6D02G327700.1	45.54
TraesCS6D02G329200.1	40.78
TraesCS7A02G152200.1	41.38
TraesCS7A02G198800.1	38.37
TraesCS7A02G198900.1	43.68
TraesCS7A02G565100.1	44.91
TraesCS7B02G056100.1	38.95
TraesCS7B02G104900.1	44.83
TraesCS7B02G105000.1	43.56
TraesCS7B02G105100.1	46.55
TraesCS7B02G105200.1	44.83
TraesCS7B02G105300.1	43.1
TraesCS7D02G099600.1	44.38
TraesCS7D02G153900.1	37.21
TraesCS7D02G161200.1	43.9
TraesCS7D02G201300.1	41.28
TraesCS7D02G201400.1	40.46
TraesCSU02G076600.1	44.05
TraesCSU02G095300.1	45.29
TraesCSU02G202900.1	44.05
TraesCSU02G226400.1	43.45
TraesCSU02G233000.1	44.05
