# Supplementary material for: Characterization and functional analyses of wheat TaPR1 genes in response to stripe rust fungal infection
Source: Sci Rep. 2023 Feb 27;13:3362. doi: 10.1038/s41598-023-30456-8 (PMC9971213; doi:10.1038/s41598-023-30456-8)

**The original image of the gel used in this article.** **The first hole of the gel is 2K marker that from top to bottom is 2000bp, 1000bp, 750bp, 500bp, 250bp, and 100bp, respectively.**


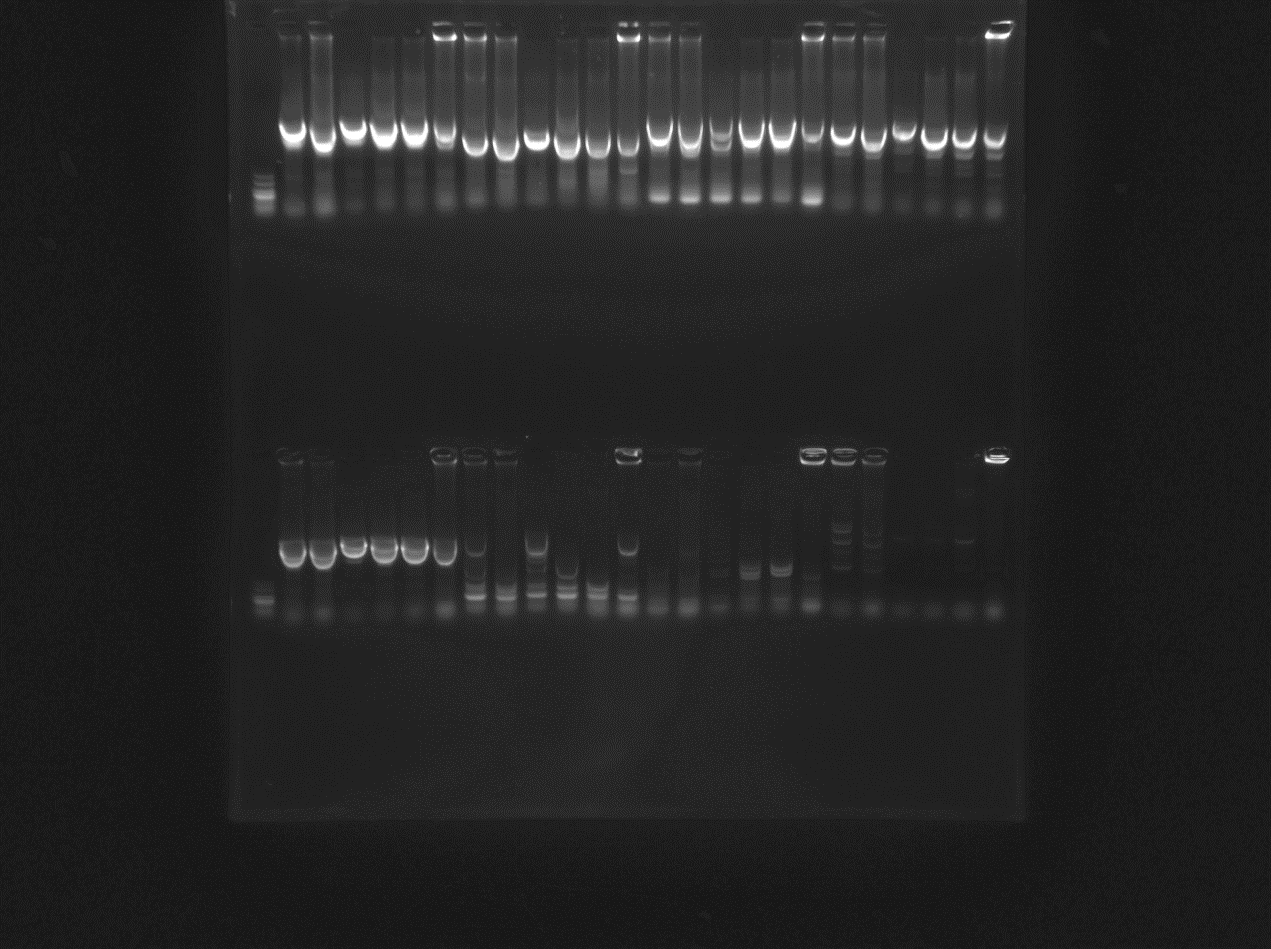


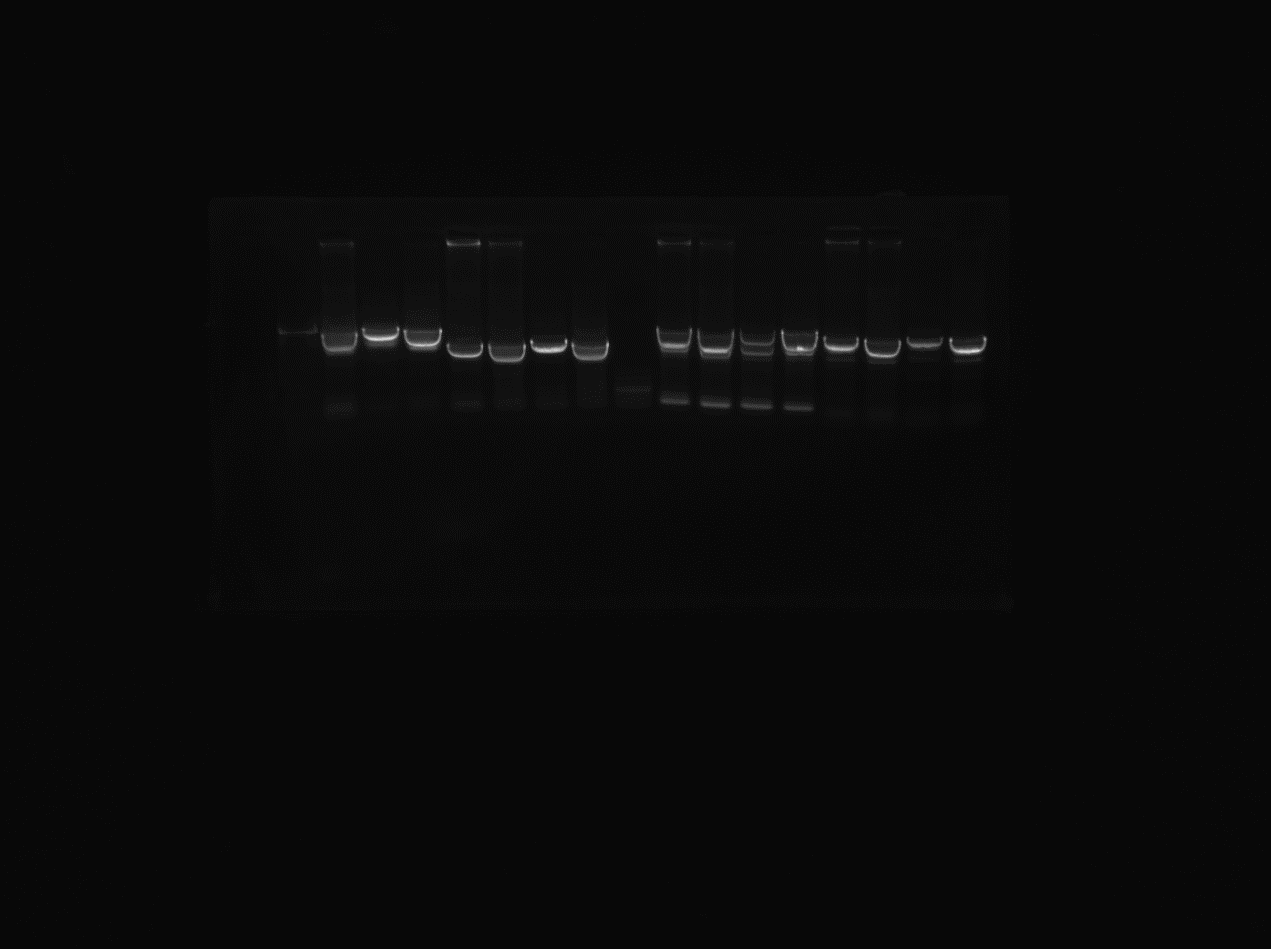


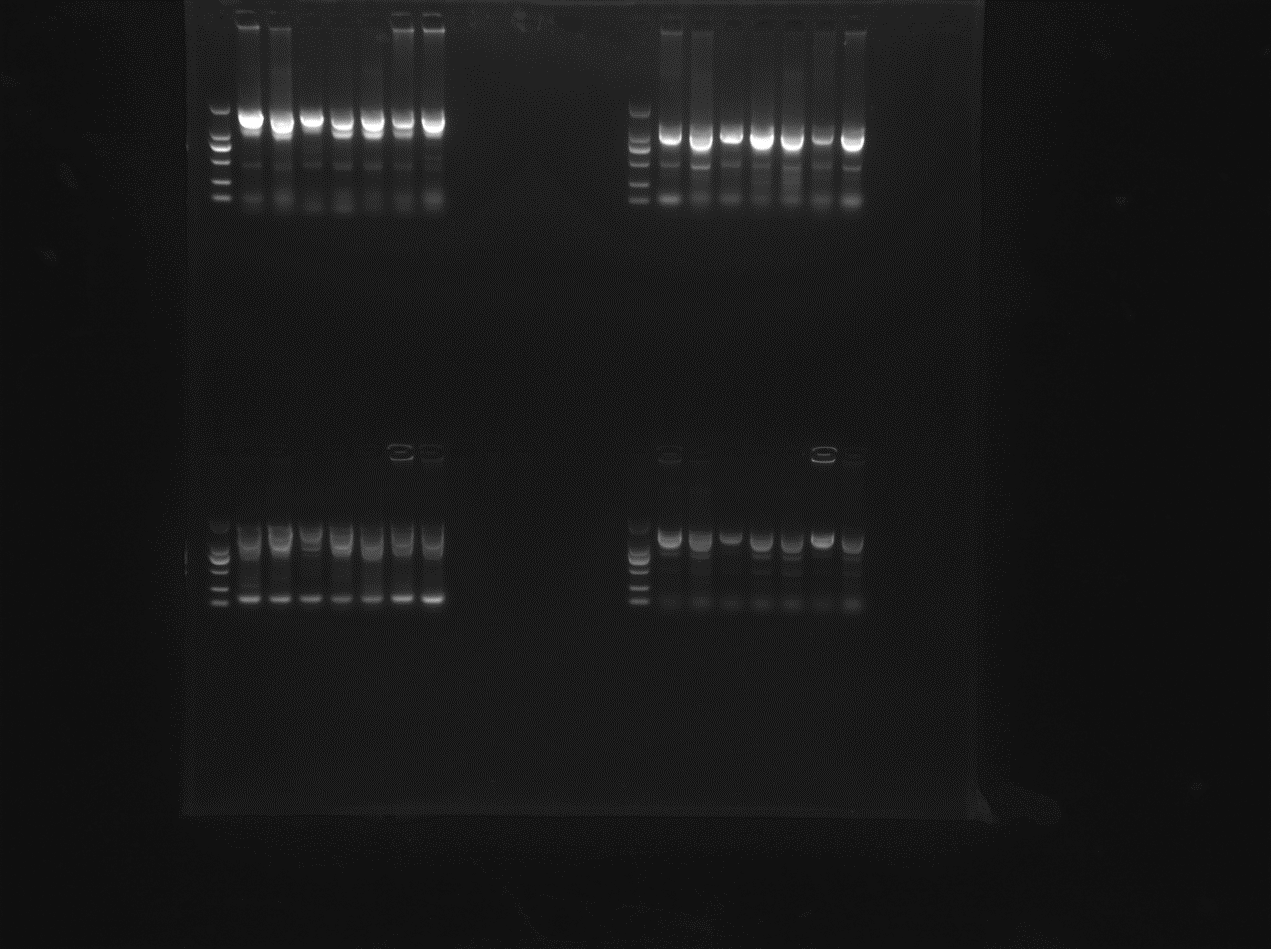


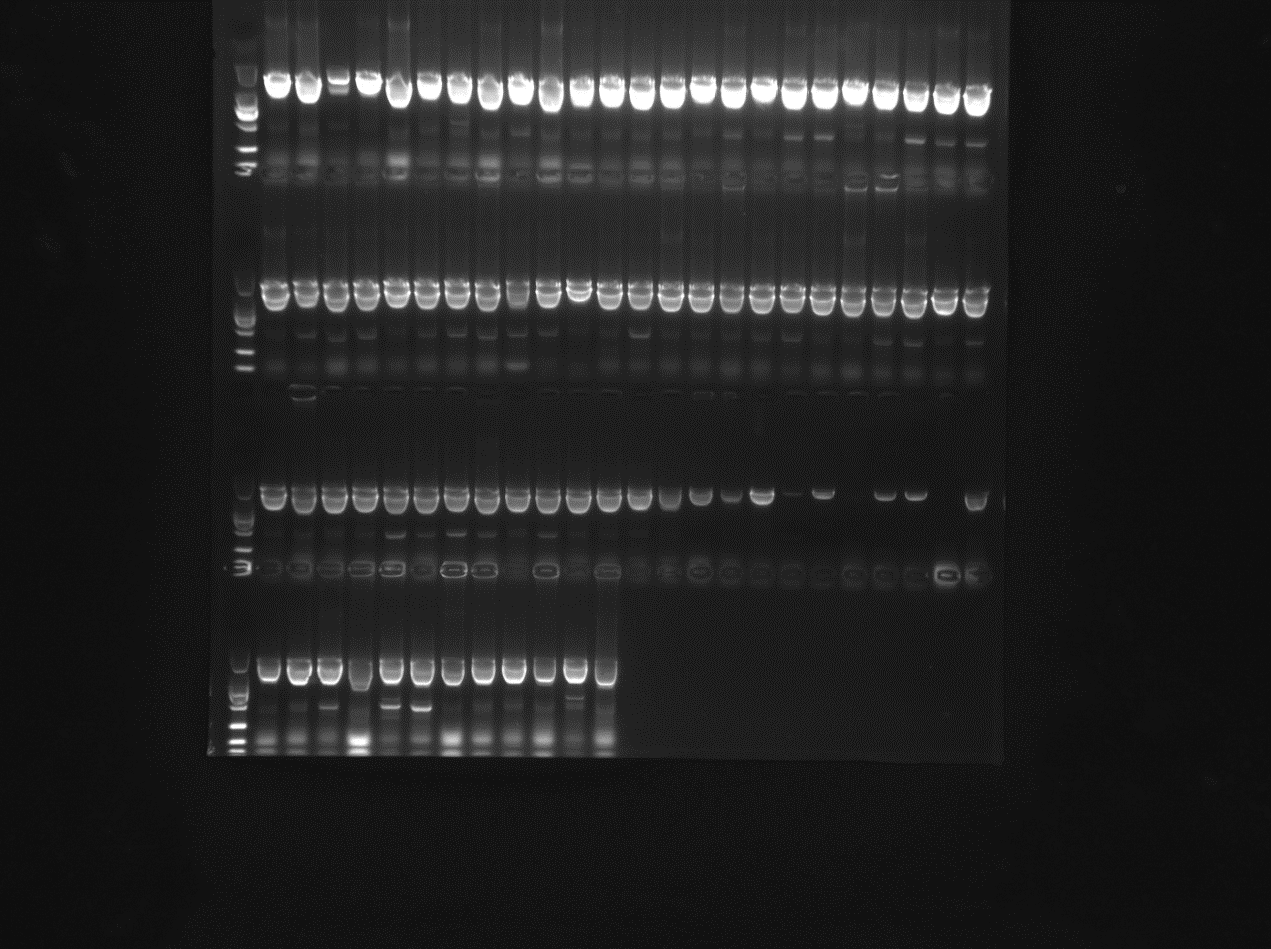


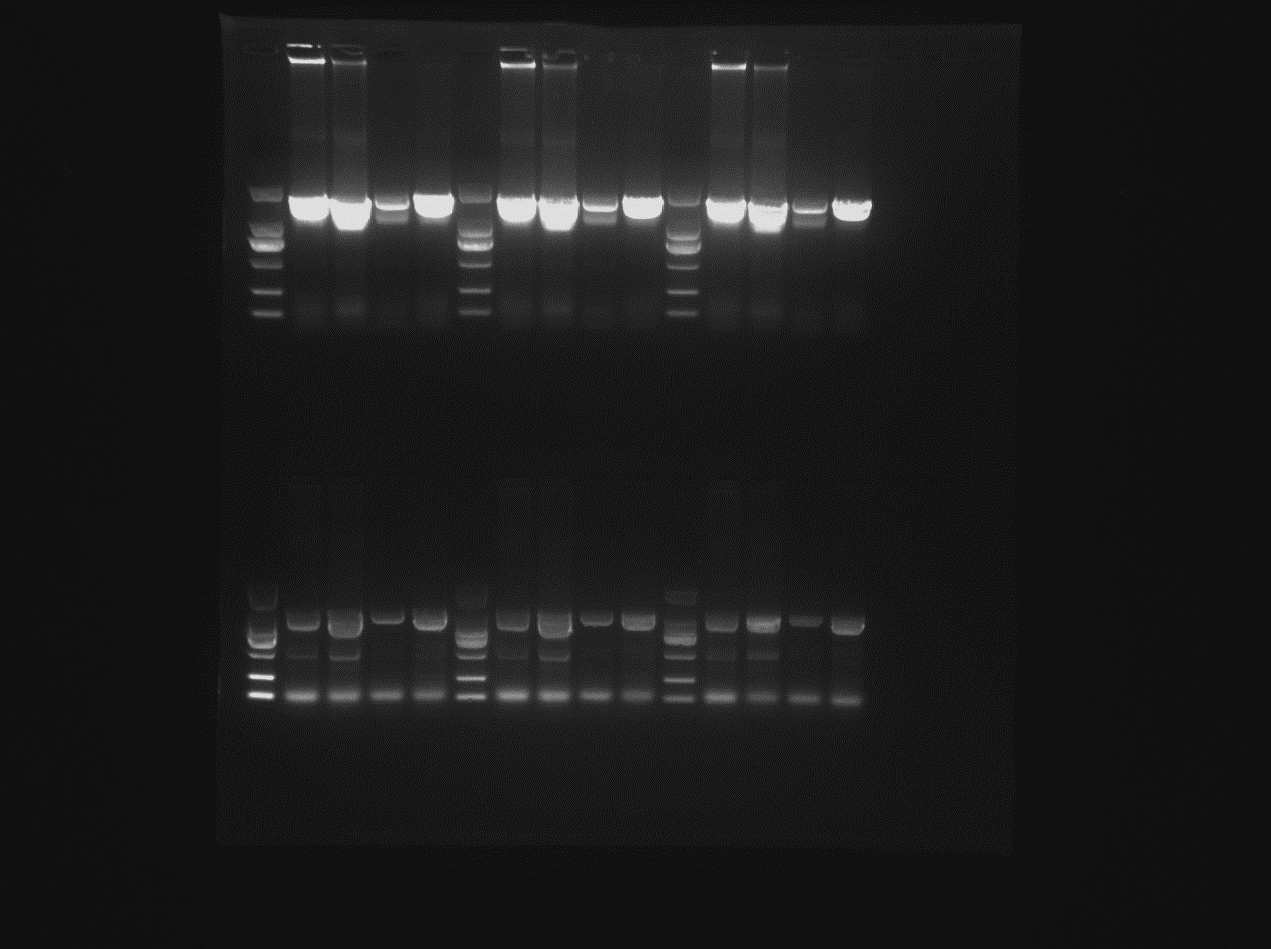

Supplement: Supplementary file 6 — Supplementary Information 6. [file 41598_2023_30456_MOESM6_ESM.docx]
