# Supplementary material for: Characterization and functional analyses of wheat TaPR1 genes in response to stripe rust fungal infection
Source: Sci Rep. 2023 Feb 27;13:3362. doi: 10.1038/s41598-023-30456-8 (PMC9971213; doi:10.1038/s41598-023-30456-8)
Supplement: Supplementary file 7 — Supplementary Information 7. [file 41598_2023_30456_MOESM7_ESM.pdf]

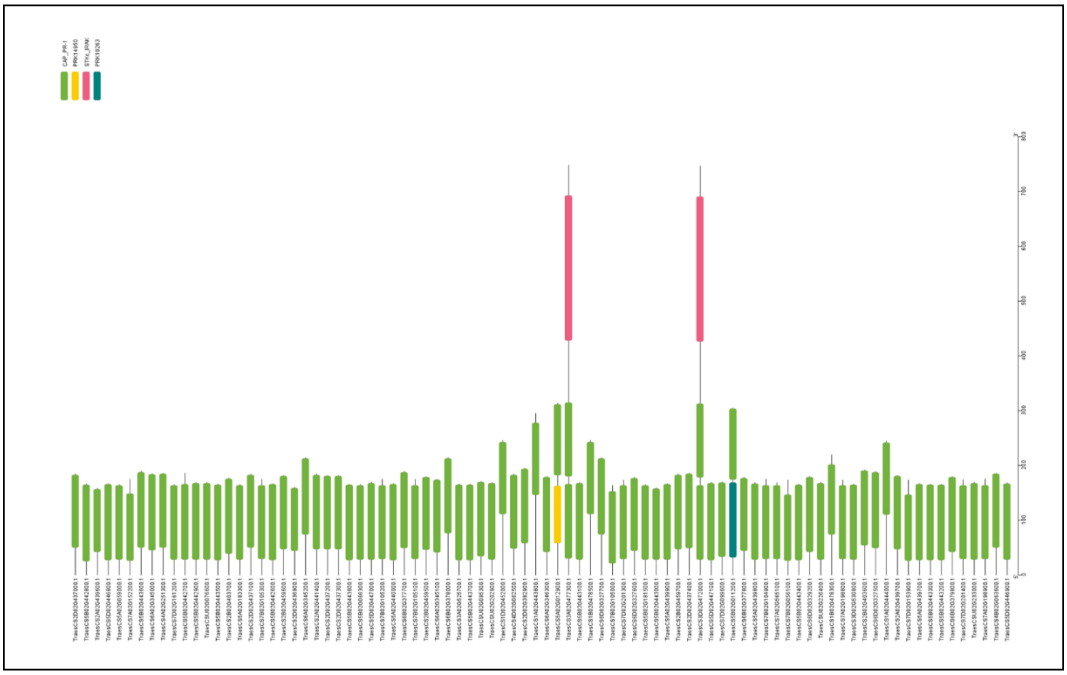

Figure S1 Functional domains of PR1 genes in wheat. This figure was created by using TBtools.

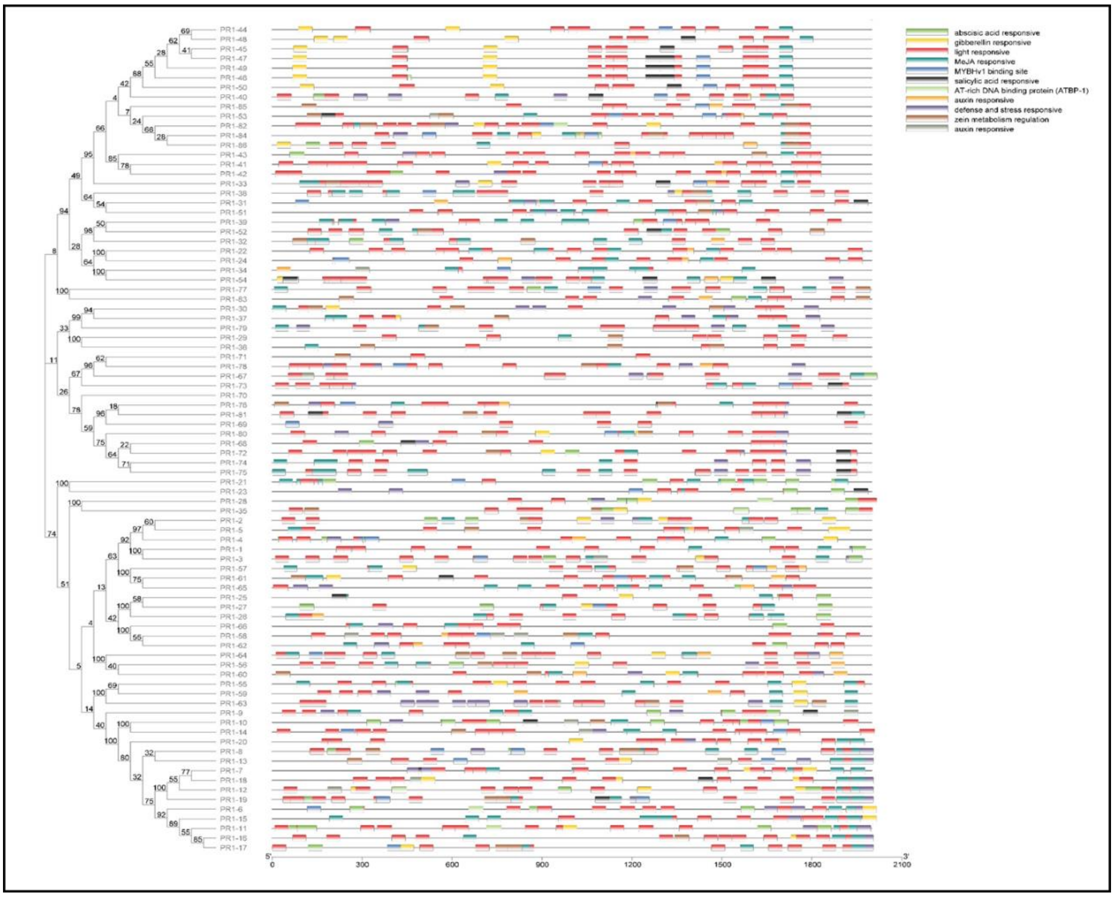

Figure S2 Predicted cis-elements in the promoter regions of the wheat PR1 genes. The different colors the box indicated different promoter elements in PR1 genes. This figure was created by using TBtools.

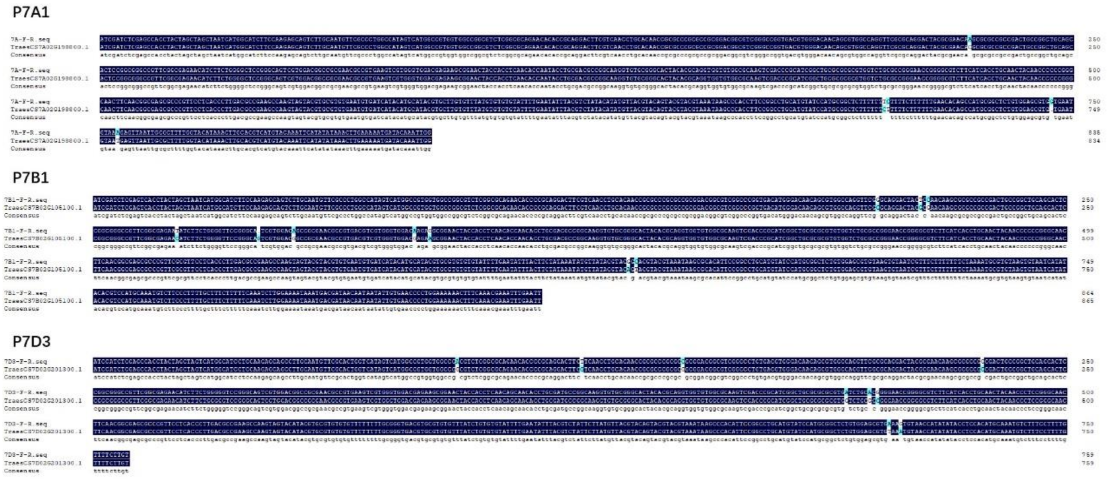

Figure S3 Three homologous genes (TaPR1-7A/7B/7D) sequence alignment. P7A1, P7B1, and P7D3 were primer names of TaPR1-7A, TaPR1-7B, and TaPR1-7D genes, respectively. This figure was created by using TBtools.

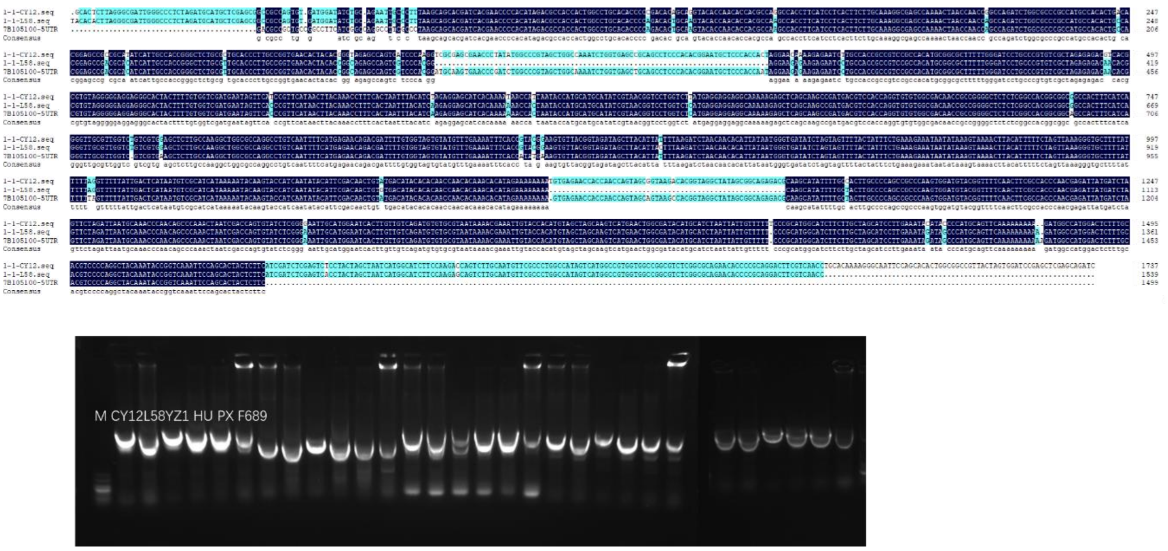

Figure S4 Sequence alignment of non-coding region in resistant and susceptible wheat, and polymorphism of *TaPR1-7B* gene functional markers (*TaPR1-7B1-cibM1*) in different wheat

population lines (YZ1/NX188 RILs). This figure was created by using TBtools.
