# Supplementary material for: Characterization and functional analyses of wheat TaPR1 genes in response to stripe rust fungal infection
Source: Sci Rep. 2023 Feb 27;13:3362. doi: 10.1038/s41598-023-30456-8 (PMC9971213; doi:10.1038/s41598-023-30456-8)
Supplement: Supplementary file 8 — Supplementary Information 8. [file 41598_2023_30456_MOESM8_ESM.docx]

Table S1The list information of the putative PR1 genes in wheat

| **Name** | **Gene ID** | **Genomic Location** | **Exons** | **Transcript length** | **pI** | **MW (kDa)** | **aa** |
| --- | --- | --- | --- | --- | --- | --- | --- |
| TaPR1-1 | TraesCS1A02G443800 | 1A:591956462-591957346 | 1 | 885 | 5.73 | 30.47 | 294 |
| TaPR1-2 | TraesCS1A02G444000 | 1A:592091804-592092538 | 1 | 735 | 8.82 | 26.92 | 244 |
| TaPR1-3 | TraesCS1B02G478300 | 1B:686757667-686758515 | 3 | 657 | 6.05 | 23.69 | 218 |
| TaPR1-4 | TraesCS1B02G478500 | 1B:686845803-686846540 | 1 | 738 | 8.70 | 26.92 | 245 |
| TaPR1-5 | TraesCS1D02G452000 | 1D:493772114-493773131 | 1 | 1018 | 8.96 | 26.85 | 245 |
| TaPR1-6 | TraesCS2A02G439600 | 2A:690747509-690748036 | 2 | 474 | 6.05 | 16.88 | 157 |
| TaPR1-7 | TraesCS2A02G439700 | 2A:690805422-690806078 | 1 | 657 | 8.79 | 19.75 | 181 |
| TaPR1-8 | TraesCS2A02G441400 | 2A:691391435-691391986 | 1 | 552 | 8.66 | 19.92 | 183 |
| TaPR1-9 | TraesCS2B02G403600 | 2B:572078859-572079434 | 1 | 576 | 8.11 | 20.67 | 191 |
| TaPR1-10 | TraesCS2B02G403700 | 2B:572079793-572080361 | 1 | 569 | 8.73 | 19.10 | 176 |
| TaPR1-11 | TraesCS2B02G459500 | 2B:653788395-653788980 | 1 | 586 | 5.23 | 19.43 | 179 |
| TaPR1-12 | TraesCS2B02G459600 | 2B:653796283-653796828 | 1 | 546 | 8.80 | 19.55 | 181 |
| TaPR1-13 | TraesCS2B02G459700 | 2B:653808218-653809010 | 1 | 793 | 9.03 | 20.03 | 183 |
| TaPR1-14 | TraesCS2D02G382900 | 2D:487315889-487316473 | 1 | 585 | 8.73 | 21.01 | 194 |
| TaPR1-15 | TraesCS2D02G436900 | 2D:547278373-547278906 | 2 | 480 | 6.04 | 17.03 | 159 |
| TaPR1-16 | TraesCS2D02G437000 | 2D:547286497-547287080 | 1 | 548 | 5.26 | 19.75 | 183 |
| TaPR1-17 | TraesCS2D02G437100 | 2D:547427179-547427730 | 1 | 552 | 5.86 | 19.89 | 183 |
| TaPR1-18 | TraesCS2D02G437200 | 2D:547450810-547451355 | 1 | 546 | 8.41 | 19.75 | 181 |
| TaPR1-19 | TraesCS2D02G437300 | 2D:547489256-547490027 | 1 | 772 | 9.08 | 19.64 | 181 |
| TaPR1-20 | TraesCS2D02G437400 | 2D:547559490-547560047 | 1 | 558 | 9.17 | 20.22 | 185 |
| TaPR1-21 | TraesCS3A02G477300 | 3A:709123866-709129930 | 8 | 2244 | 6.17 | 80.78 | 747 |
| TaPR1-22 | TraesCS3A02G525700 | 3A:739640193-739640917 | 1 | 725 | 4.85 | 17.65 | 165 |
| TaPR1-23 | TraesCS3D02G472000 | 3D:574118271-574125655 | 8 | 2603 | 5.82 | 80.84 | 746 |
| TaPR1-24 | TraesCS3D02G530800 | 3D:607168074-607168571 | 1 | 498 | 4.85 | 17.60 | 165 |
| TaPR1-25 | TraesCS4A02G251300 | 4A:562457963-562459338 | 1 | 1376 | 9.36 | 19.23 | 185 |
| TaPR1-26 | TraesCS4B02G063600 | 4B:55726240-55727285 | 1 | 1406 | 9.10 | 19.06 | 185 |
| TaPR1-27 | TraesCS4D02G062500 | 4D:38344234-38345364 | 1 | 1131 | 9.24 | 19.03 | 183 |
| TaPR1-28 | TraesCS5A02G012900 | 5A:8656997-8658492 | 2 | 1407 | 5.14 | 33.63 | 312 |
| TaPR1-29 | TraesCS5A02G059000 | 5A:59565139-59565633 | 1 | 495 | 7.61 | 17.75 | 164 |
| TaPR1-30 | TraesCS5A02G183300 | 5A:382777720-382778535 | 1 | 816 | 8.52 | 17.57 | 164 |
| TaPR1-31 | TraesCS5A02G439700 | 5A:621023309-621024091 | 1 | 783 | 4.40 | 17.83 | 166 |
| TaPR1-32 | TraesCS5A02G439800 | 5A:621177195-621177698 | 1 | 504 | 4.82 | 17.80 | 167 |
| TaPR1-33 | TraesCS5A02G439900 | 5A:621444299-621449202 | 2 | 501 | 4.47 | 17.56 | 166 |
| TaPR1-34 | TraesCS5A02G440000 | 5A:621528283-621528783 | 1 | 501 | 5.85 | 17.80 | 166 |
| TaPR1-35 | TraesCS5B02G011200 | 5B:11074579-11076006 | 2 | 1322 | 5.25 | 32.95 | 304 |
| TaPR1-36 | TraesCS5B02G066300 | 5B:74508631-74509125 | 1 | 495 | 8.52 | 17.63 | 164 |
| TaPR1-37 | TraesCS5B02G181500 | 5B:331247621-331248115 | 1 | 495 | 8.74 | 17.63 | 164 |
| TaPR1-38 | TraesCS5B02G442600 | 5B:615736560-615737060 | 1 | 501 | 4.47 | 17.70 | 166 |
| TaPR1-39 | TraesCS5B02G442700 | 5B:615741711-615742268 | 1 | 558 | 5.18 | 19.87 | 185 |
| TaPR1-40 | TraesCS5B02G442800 | 5B:615951078-615951575 | 1 | 498 | 4.28 | 17.55 | 165 |
| TaPR1-41 | TraesCS5B02G442900 | 5B:616026070-616027135 | 1 | 870 | 4.28 | 17.92 | 168 |
| TaPR1-42 | TraesCS5B02G443000 | 5B:616104269-616106253 | 2 | 477 | 4.38 | 16.89 | 158 |
| TaPR1-43 | TraesCS5B02G443100 | 5B:616160236-616164321 | 1 | 742 | 4.28 | 17.88 | 168 |
| TaPR1-44 | TraesCS5B02G443200 | 5B:616223411-616223908 | 1 | 498 | 4.28 | 17.76 | 165 |
| TaPR1-45 | TraesCS5B02G443300 | 5B:616245567-616246481 | 1 | 678 | 4.28 | 17.80 | 165 |
| TaPR1-46 | TraesCS5B02G443400 | 5B:616265360-616266511 | 1 | 915 | 4.28 | 17.77 | 165 |
| TaPR1-47 | TraesCS5B02G443500 | 5B:616276669-616277166 | 1 | 498 | 4.28 | 17.80 | 165 |
| TaPR1-48 | TraesCS5B02G443600 | 5B:616299596-616300162 | 1 | 567 | 4.26 | 20.10 | 188 |
| TaPR1-49 | TraesCS5B02G443700 | 5B:616322233-616322730 | 1 | 498 | 4.28 | 17.77 | 165 |
| TaPR1-50 | TraesCS5B02G443800 | 5B:616332941-616333438 | 1 | 498 | 4.42 | 17.81 | 165 |
| TaPR1-51 | TraesCS5D02G446800 | 5D:496956859-496957660 | 1 | 802 | 4.47 | 17.73 | 166 |
| TaPR1-52 | TraesCS5D02G446900 | 5D:496978545-496979616 | 1 | 977 | 4.58 | 17.82 | 167 |
| TaPR1-53 | TraesCS5D02G447000 | 5D:497038493-497039114 | 1 | 622 | 4.26 | 17.87 | 168 |
| TaPR1-54 | TraesCS5D02G447100 | 5D:497303413-497304148 | 1 | 736 | 6.26 | 18.10 | 168 |
| TaPR1-55 | TraesCS6A02G345000 | 6A:578941981-578943067 | 1 | 1087 | 10.41 | 19.33 | 184 |
| TaPR1-56 | TraesCS6A02G345100 | 6A:578954695-578955570 | 1 | 876 | 8.83 | 18.92 | 174 |
| TaPR1-57 | TraesCS6A02G345200 | 6A:578983107-578983748 | 1 | 642 | 7.05 | 22.79 | 213 |
| TaPR1-58 | TraesCS6A02G346300 | 6A:579504726-579505715 | 1 | 990 | 8.92 | 19.05 | 179 |
| TaPR1-59 | TraesCS6B02G377700 | 6B:652945270-652946131 | 1 | 862 | 10.37 | 19.82 | 188 |
| TaPR1-60 | TraesCS6B02G377800 | 6B:652983633-652984166 | 1 | 534 | 8.83 | 19.16 | 177 |
| TaPR1-61 | TraesCS6B02G378000 | 6B:653014981-653015923 | 1 | 943 | 8.03 | 22.84 | 213 |
| TaPR1-62 | TraesCS6B02G379800 | 6B:654775115-654775965 | 1 | 851 | 9.05 | 19.08 | 179 |
| TaPR1-63 | TraesCS6D02G327500 | 6D:432823804-432824996 | 1 | 1106 | 10.29 | 19.83 | 188 |
| TaPR1-64 | TraesCS6D02G327600 | 6D:432852692-432853601 | 1 | 910 | 8.83 | 19.17 | 177 |
| TaPR1-65 | TraesCS6D02G327700 | 6D:432884524-432885165 | 1 | 642 | 7.11 | 22.85 | 213 |
| TaPR1-66 | TraesCS6D02G329200 | 6D:433490754-433491722 | 1 | 969 | 8.73 | 19.15 | 179 |
| TaPR1-67 | TraesCS7A02G152200 | 7A:105625827-105627785 | 2 | 833 | 6.93 | 18.78 | 174 |
| TaPR1-68 | TraesCS7A02G198800 | 7A:161279855-161280689 | 1 | 835 | 9.02 | 18.62 | 172 |
| TaPR1-69 | TraesCS7A02G198900 | 7A:161650540-161651403 | 1 | 864 | 7.63 | 18.90 | 174 |
| TaPR1-70 | TraesCS7A02G565100 | 7A:734538573-734539170 | 1 | 598 | 9.86 | 18.37 | 167 |
| TaPR1-71 | TraesCS7B02G056100 | 7B:59094983-59096599 | 2 | 519 | 8.46 | 18.62 | 172 |
| TaPR1-72 | TraesCS7B02G104900 | 7B:121053481-121054324 | 1 | 844 | 9.17 | 18.84 | 174 |
| TaPR1-73 | TraesCS7B02G105000 | 7B:121115520-121116985 | 1 | 763 | 11.62 | 17.94 | 163 |
| TaPR1-74 | TraesCS7B02G105100 | 7B:121242521-121243386 | 1 | 866 | 9.02 | 18.84 | 174 |
| TaPR1-75 | TraesCS7B02G105200 | 7B:121320794-121321318 | 1 | 525 | 9.02 | 18.85 | 174 |
| TaPR1-76 | TraesCS7B02G105300 | 7B:121496003-121496779 | 1 | 777 | 7.63 | 18.80 | 174 |
| TaPR1-77 | TraesCS7D02G099600 | 7D:59873070-59873910 | 1 | 841 | 7.53 | 18.16 | 169 |
| TaPR1-78 | TraesCS7D02G153900 | 7D:101759384-101761767 | 2 | 519 | 8.14 | 18.61 | 172 |
| TaPR1-79 | TraesCS7D02G161200 | 7D:111114487-111115253 | 1 | 767 | 8.75 | 17.54 | 164 |
| TaPR1-80 | TraesCS7D02G201300 | 7D:160487672-160488431 | 1 | 760 | 9.17 | 18.66 | 172 |
| TaPR1-81 | TraesCS7D02G201400 | 7D:160534128-160535018 | 1 | 891 | 6.88 | 18.80 | 173 |
| TaPR1-82 | TraesCSU02G076600 | Un:68273551-68274057 | 1 | 507 | 4.28 | 17.88 | 168 |
| TaPR1-83 | TraesCSU02G095300 | Un:84034643-84035380 | 1 | 738 | 7.53 | 18.45 | 170 |
| TaPR1-84 | TraesCSU02G202900 | Un:302517563-302518069 | 1 | 507 | 4.28 | 17.88 | 168 |
| TaPR1-85 | TraesCSU02G226400 | Un:335555235-335555741 | 1 | 507 | 4.40 | 17.89 | 168 |
| TaPR1-86 | TraesCSU02G233000 | Un:345125707-345126213 | 1 | 507 | 4.28 | 17.88 | 168 |
